# *De novo* genome assembly and Hi-C analysis reveal an association between chromatin architecture alterations and sex differentiation in the woody plant *Jatropha curcas*

**DOI:** 10.1093/gigascience/giaa009

**Published:** 2020-02-12

**Authors:** Mao-Sheng Chen, Longjian Niu, Mei-Li Zhao, Chuanjia Xu, Bang-Zhen Pan, Qiantang Fu, Yan-Bin Tao, Huiying He, Chunhui Hou, Zeng-Fu Xu

**Affiliations:** 1 CAS Key Laboratory of Tropical Plant Resources and Sustainable Use, Xishuangbanna Tropical Botanical Garden, The Innovative Academy of Seed Design, Chinese Academy of Sciences, Menglun, Mengla, Yunnan 666303, China; 2 Center of Economic Botany, Core Botanical Gardens, Chinese Academy of Sciences, Menglun, Mengla, Yunnan 666303, China; 3 Department of Biology, Southern University of Science and Technology, 1088 Xueyuan Rd., Shenzhen, Guangdong 518055, China; 4 Department of Biology, Nankai University, 94 Weijing Rd., Tianjin 660885, China; 5 College of Life Sciences, University of Chinese Academy of Sciences, 19(A) Yuquan Rd., Beijing 100049, China

**Keywords:** high-quality genome, Hi-C, sex determination, chromatin architecture, *Jatropha*

## Abstract

**Background:**

Chromatin architecture is an essential factor regulating gene transcription in different cell types and developmental phases. However, studies on chromatin architecture in perennial woody plants and on the function of chromatin organization in sex determination have not been reported.

**Results:**

Here, we produced a chromosome-scale *de novo* genome assembly of the woody plant *Jatropha curcas* with a total length of 379.5 Mb and a scaffold N50 of 30.7 Mb using Pacific Biosciences long reads combined with genome-wide chromosome conformation capture (Hi-C) technology. Based on this high-quality reference genome, we detected chromatin architecture differences between monoecious and gynoecious inflorescence buds of *Jatropha*. Differentially expressed genes were significantly enriched in the changed A/B compartments and topologically associated domain regions and occurred preferentially in differential contact regions between monoecious and gynoecious inflorescence buds. Twelve differentially expressed genes related to flower development or hormone synthesis displayed significantly different genomic interaction patterns in monoecious and gynoecious inflorescence buds. These results demonstrate that chromatin organization participates in the regulation of gene transcription during the process of sex differentiation in *Jatropha*.

**Conclusions:**

We have revealed the features of chromatin architecture in perennial woody plants and investigated the possible function of chromatin organization in *Jatropha* sex differentiation. These findings will facilitate understanding of the regulatory mechanisms of sex determination in higher plants.

## Introduction

Flowering plants have extremely diverse reproductive systems that are controlled by both genetic factors and environmental cues [1]. For optimal outcrossing and efficient resource allocation, ∼10% of angiosperm species have evolved reproductive systems with unisexual flowers, in which the male and female reproductive organs are physically separated; these plant taxa are termed dioecious or monoecious [2, 3]. Sex determination has evolved independently multiple times, and various regulatory mechanisms control this process [4–6]. During sex determination in cucumber, the *femaleness* (*F*) locus controls the degree of femaleness, the *androecious* (*A*) locus promotes maleness, and the *andromonoecious* (*M*) locus is responsible for the selective abortion of stamens [7]. The *F* locus has been linked to the 1-aminocyclopropane-1 carboxylic acid synthase (*CsACS1*) gene, which occurs as a single copy in monoecious lines but is duplicated in gynoecious lines [8, 9], while the *M* locus has been linked to the *CsACS2* gene, and a conserved residue conversion (Gly33Cys) in *CsACS2* causes the generation of bisexual flowers in cucumber [10]. The *andromonoecious* (*a*) and *gynoecious* (*g*) loci control sex determination in melon [11]. The *a* locus has been linked to the *GmACS-7* gene, and loss of function of *GmACS-7* causes male organ development, generating andromonoecious plants [12]; the *g* locus encodes a repressor of carpel development, CmWIP1, activation of which causes a transition from male to female flowers in gynoecious plants [13]. In addition, CmACS-11 inhibits the expression of *CmWIP1*, and loss of function of *CmACS-11* results in a transition from monoecious to androecious individuals [14]. In *Diospyros*, an autosomal *MeGI* gene regulates anther fertility, and a Y-chromosome *OGI* gene encodes a small RNA that suppresses the expression of *MeGI*, resulting in the generation of androecious individuals [15]. In maize, the *tasselseed1* (*ts1*) gene encodes a lipoxygenase involved in jasmonic acid (JA) biosynthesis, and the *ts1* mutant has defective stamen development because of a low JA concentration [16]. *tasselseed2* (*ts2*) encodes a short-chain alcohol dehydrogenase and is required for the arrest of pistil primordium development [17]. *tasselseed4* (*ts4*) encodes a microRNA, miR172, that targets *tasselseed6* (*Ts6*)/*indeterminate spikelet1* (*ids1*), and both *ts2* and *ts4* are essential for suppression of carpel development [18]. *nana plant1* (*na1*) encodes a 5α-steroid reductase involved in brassinosteroid (BR) biosynthesis, and the *na1* mutant displays dwarf and feminized phenotypes [19]. In addition, exogenous application of auxin, BR, cytokinin (CK), ethylene (ETH), gibberellin (GA), JA, and their inhibitors also affects sexual expression in several species [7, 20–23]. Temperature, photoperiod, nutrition, drought, pH, and seasonality further influence sex differentiation, and epigenetic mechanisms are likely involved in the process [24–27]. In *Jatropha*, treatment with 6-benzyladenine (BA, a synthetic compound with CK activity) significantly increases the number of female flowers, in which a *Superman* orthologue (*JcSUP*) is upregulated, while *tasselseed2* orthologue (*JcTS2*) is downregulated [28–30]. Treatment with paclobutrazol, a GA biosynthesis inhibitor, downregulates the expression of the orthologous genes *JcHUA1, no pollen germination-related 2* (*JcNPGR2*), *male*gametophyte defective 2 (JcMGP2), and JcMGP3, and increases the number of female flowers in *Jatropha* [31]. As shown by the above findings, sex differentiation is a complicated process that is mediated by both genetic and environmental factors, and the regulatory mechanisms of sex differentiation are diverse among various species.

Eukaryotic chromatin is packed into highly ordered and hierarchical structures, which contributes to the regulation of gene expression in different cell types and developmental phases [32, 33]. This well-ordered 3D chromatin architecture is essential for gene transcription, DNA replication, and genome integrity [34–36]. According to genome-wide interaction patterns, each chromosome can be partitioned into 3 hierarchical chromatin structures: A/B compartments, topologically associated domains (TADs), and chromatin loops [36–39]. The A/B compartments are associated with euchromatic (active) and heterochromatic (inactive) chromatin regions in which genomic and epigenetic features are distinct [37]. TADs are predominant chromatin structural units, and local interactions occur with far greater frequency within TADs than at the boundary between 2 TADs [36, 40]. TADs can spatially confine the interactions between promoters and distal regulatory elements, facilitating the activation of transcription, and are well correlated with markers of chromatin activity [35, 38]. Chromatin loops bring genes and their regulatory elements, such as enhancers and promoters, into close proximity for direct interactions [33, 41]. Multiple enhancer-promoter combinations can share binding of common transcription factors to establish a chromatin environment more permissive to transcription than that created by a single enhancer-promoter combination [42]. In plants, similar chromatin architectures have been identified in the genomes of several crop species, such as rice, maize, tomato, sorghum, and foxtail millet, but they are not conserved across these species, suggesting that chromatin organizations are complex and unique in higher plants [43, 44].

*Jatropha curcas* L. (NCBI:txid180498), a perennial woody plant, is known as a potential biofuel crop because of its high seed oil content [45, 46]. At present, 4 different *Jatropha* genome assemblies have been reported [47–50], but they are insufficient to meet the requirements of chromatin architecture analysis, which requires a high-quality reference genome. *Jatropha* has 2 different ecotypes, monoecious and gynoecious. Monoecious plants bear male and female flowers separately on the same inflorescence; in contrast, gynoecious plants bear only female flowers because their male flowers are aborted at an early stage of inflorescence development [51, 52]. In this study, we produced a chromosome-scale *Jatropha* assembly using a combination of single-molecule Pacific Biosciences (PacBio) sequencing and genome-wide chromosome conformation capture (Hi-C) technology [53, 54]. Based on this high-quality reference genome, we investigated the function of chromatin architecture during sex differentiation by comparing chromatin architectures and transcriptomes between monoecious and gynoecious *Jatropha* inflorescence buds. Our results will facilitate the elucidation of sex determination in *Jatropha* and clarify the biological functions of chromatin architecture in higher plants.

## Results

### Chromosome-scale *Jatropha* genome assembly

PacBio long-read sequencing data (33.41 Gb) were used for *de novo* assembly of the *Jatropha* genome (Additional Fig. S1). The sequence coverage was ∼80× based on the genome size (416 Mb), as estimated with flow cytometry [55]. The first round of genome assembly was performed using the FALCON package (version 0.3.0) [56], and then polishing was performed using the arrow algorithm in Pacific SMRT Link (version 5.1.0). The assembly was composed of 1,265 contigs with a total length of 378.3 Mb and an N50 value of 1.0 Mb (Table 1). The 3D proximity information obtained via the Hi-C sequencing data was used to correct instances of misjoining and to order and orient the contig assembly; then, the results were integrated into a candidate chromosome-scale assembly using the 3D *de novo* assembly (3D DNA) pipeline [54]. The candidate assembly was further improved by interactive correction using Juicebox Assembly Tools [57]. The final *Jatropha* assembly (hereafter referred to as our *Jatropha* assembly) had a total length of 379.1 Mb and an N50 value of 30.7 Mb and contained 11 complete chromosomes (each chromosome >27.1 Mb) (Table 1). After masking of repetitive sequences, 25,817 protein-coding genes were predicted based on transcript and protein alignments using the MAKER annotation pipeline (version 2.31.10) [58, 59] (Table 1). The annotation of our *Jatropha* assembly had a high annotation edit distance (AED) score (Additional Fig. S2) [60], suggesting that it was a high-quality genome annotation.

**Table 1:** Statistics of our *Jatropha* genome assembly

Assembly	No.	N50 (bp)	N75 (bp)	L50 (No.)	L75 (No.)	Total length (bp)
Contigs	1,265	1,029,648	362,618	86	246	378,337,367
Scaffolds	1,196	30,651,357	27,306,515	6	10	379,507,867
Chromosomes	11	-	-	-	-	337,277,379
Coding genes	25,817	-	-	-	-	40,884,597

### Quality evaluation of the new *Jatropha* assembly

**Figure 1:**
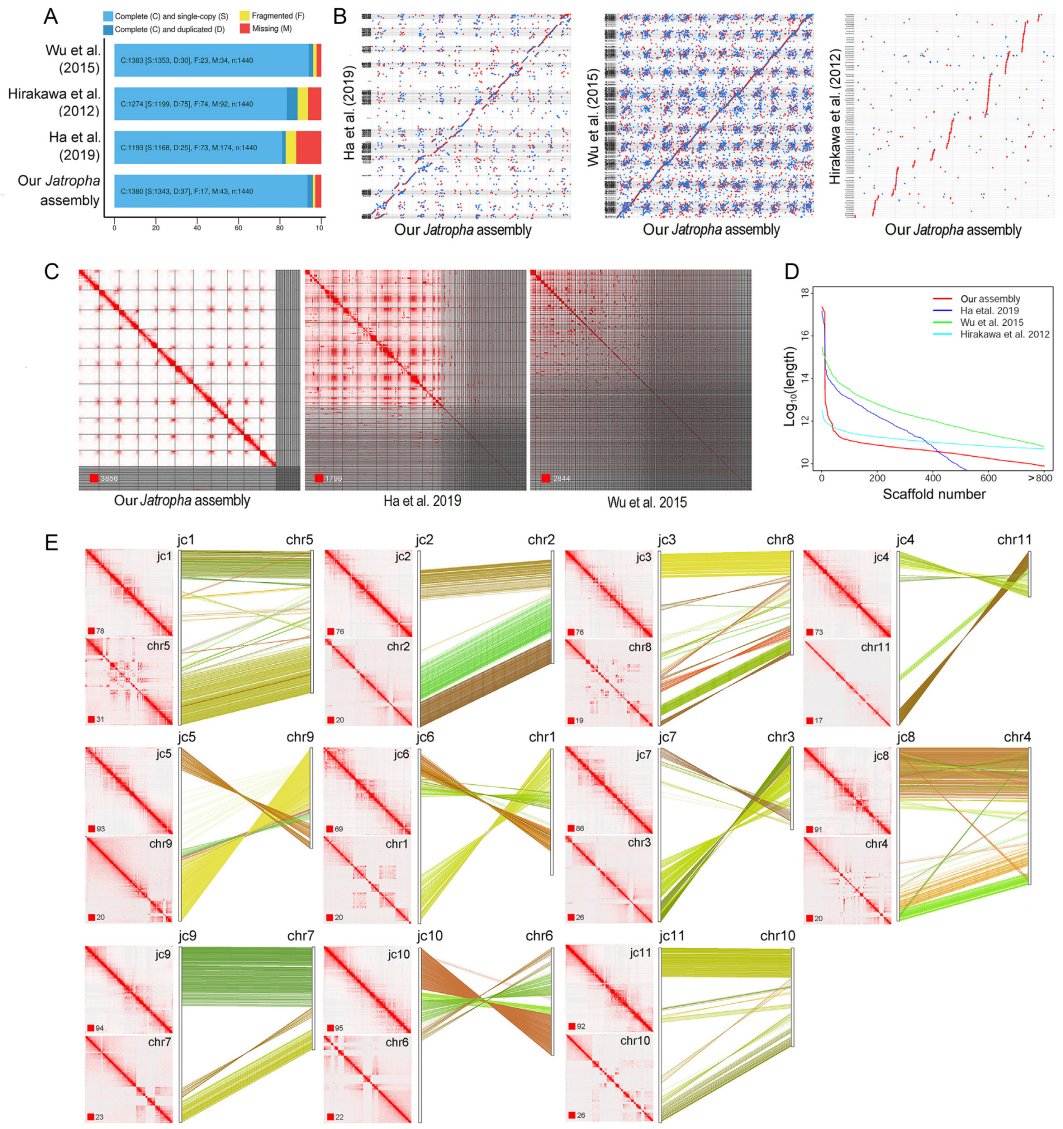
Genome comparison between our assembly and the published *Jatropha* assemblies. (A) BUSCO annotation of our assembly and the published *Jatropha* assemblies. n represents the number of single-copy orthologous genes. (B) Collinearity analysis of entire genome sequences between our assembly and the published *Jatropha* assemblies. (C) Comparison of Hi-C contact maps among our *Jatropha* genome assembly and the other 2 assemblies. The red square represents the strongest signal value. (D) Distribution of sequence length in our assembly and the published *Jatropha* assemblies. (E) Comparison of corresponding chromosomes between our assembly and the Ha et al. (2019) assembly [47–50]. “jc1-11” indicates the chromosome codes of our *Jatropha* assembly, and “chr1-11” indicates the chromosome codes of the Ha et al. (2019) *Jatropha* assembly [47–50].

We calculated small local errors in the new *Jatropha* assembly, such as single-base substitutions, short insertions, and deletions, with PacBio long-read alignments using the arrow algorithm in PacBio SMRT Link (version 5.1.0). The estimated error rate was 0.22% (substitutions, 0.17%; insertions, 0.03%; and deletions, 0.02%). However, the actual error rate should be far smaller than the estimated rate because a large number of false errors could have been introduced into the genome sequence by the heterozygosity of the *Jatropha* genome. The completeness and contiguity were assessed using the QUAST-LG, BUSCO (version 3.0), mummer (version 4.0), and MCScanX packages [61–64]. The BUSCO results showed that our assembly was more complete than the published *Jatropha* genome assemblies (Fig. 1A) [47–49]. Comparison of the genome sequences showed that our assembly and the published *Jatropha* assemblies had similar genomic structures (Fig. 1B) [47–50], but the completeness and contiguity of our *Jatropha* assembly were better than those of the other assemblies (Fig. 1A, D, and E, Additional Table S1 and Additional Fig. S3). Moreover, we compared Hi-C interaction maps across our assembly and the previous *Jatropha* assemblies by mapping Hi-C sequencing reads to the respective reference genomes, and our *Jatropha* assembly displayed perfect completeness and contiguity in this analysis (Fig. 1C and E).

### Features of chromatin architecture in the new *Jatropha* genome

**Figure 2:**
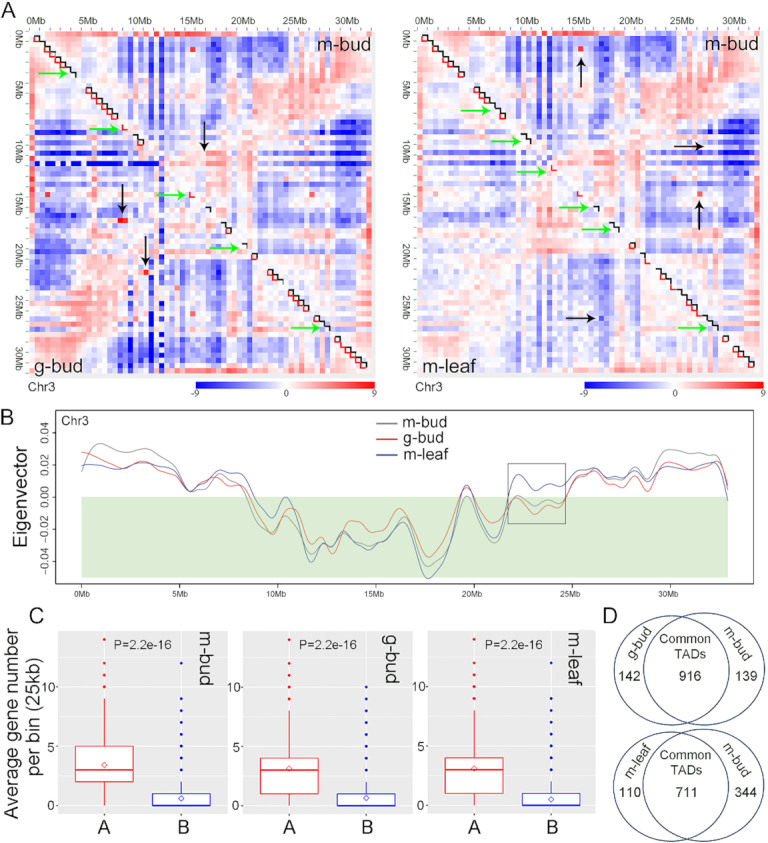
Chromatin architecture changes in the g-bud vs m-bud and m-leaf vs m-bud comparisons. (A) Comparison of the chromatin architecture of chromosome 3 in g-bud vs m-bud samples and in m-leaf vs m-bud samples. The black arrows indicate the changed A/B compartment regions, and the green arrows indicate the changed TAD regions. The legends indicate the interaction strength (observed/expected). (B) A/B compartments of chromosome 3 across m-bud, g-bud, and m-leaf samples. The black box indicates the changed regions; the shaded green area indicates the B compartment region. (C) Protein-coding gene distribution in A/B compartment regions across the m-bud, g-bud, and m-leaf samples. The diamond represents the mean value. A and B represent the A compartment and B compartment, respectively. Statistical tests were carried out using the Welch 2-sample *t*-test in R software (https://cran.r-project.org). (D) Comparison of TADs in g-bud vs m-bud samples and in m-leaf vs m-bud samples. The number represents the number of TADs. The label “m-bud” indicates monoecious inflorescence bud samples, the label “g-bud” indicates gynoecious inflorescence bud samples, and the label “m-leaf” indicates monoecious leaf samples.

We investigated the chromatin architecture of our *Jatropha* genome with the Hi-C method [37]. Three types of Hi-C libraries were constructed: “m-bud” Hi-C libraries from monoecious inflorescence buds, “m-leaf” Hi-C libraries from monoecious leaves, and “g-bud” Hi-C libraries from gynoecious inflorescence buds. Two biological replicates per sample were generated (Additional Table S2). The biological replicates had a high correlation coefficient (Additional Fig. S4). Three 2D contact maps were generated to display the chromatin architectures of the m-bud, g-bud, and m-leaf samples (Additional Fig. S5). Each chromosome region was partitioned into alternating positive and negative eigenvectors representing the A/B compartments using principal component analysis (Fig. 2B and Additional Fig. S6). The average number of protein-coding genes in the A compartment regions was significantly higher than that in the B compartment regions (Fig. 2C); the A and B compartments correspond to euchromatic and heterochromatic regions, which are the important chromatin structural units in both animals and plants [37, 39, 65]. The local differences in the A/B compartments in the g-bud vs m-bud and m-leaf vs m-bud comparisons implied that chromatin organization was varied (Fig. 2A and B and Additional Fig. S6), which may have been associated with the different phenotypes or tissues.

**Figure 3:**
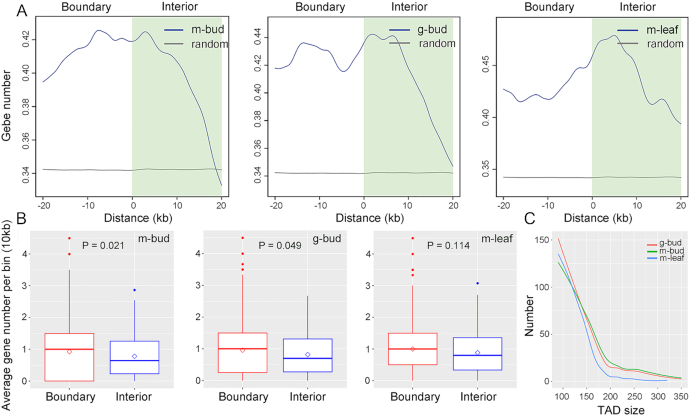
Distribution of protein-coding genes around TADs across the m-bud, g-bud, and m-leaf samples. (A) Gene distribution around TAD boundaries. The left area indicates TAD boundary regions, and the shaded green area indicates TAD interior regions. (**B)** Comparison of gene density between TAD boundary and interior regions. The box represents the middle 50% score; the upper and lower whiskers represent the scores outside the middle 50% score; the dots represent the outliers; the line inside the box represents middle quartile; the diamond inside the box represents the mean value. Statistical tests were performed using the Welch 2-sample *t*-test in R software. (C) Distribution of TAD sizes at 10 kb resolution in the m-bud, g-bud, and m-leaf samples. The label “m-bud” indicates monoecious inflorescence bud samples, the label “g-bud” indicates gynoecious inflorescence bud samples, and the label “m-leaf” indicates monoecious leaf samples.

TADs are principal chromatin structural units; notably, the frequency of chromatin interactions within TADs is higher than that within the boundary regions and reflects the presence of distinct and autonomously regulated regions of chromosomes [33, 34, 36, 40]. In *Jatropha*, we detected 1,055, 1,058, and 821 TAD-like domains at 10 kb resolution from the m-bud, g-bud, and m-leaf samples, respectively, with the arrowhead algorithm in the Juicer pipeline [66]. The median length of the TADs was 90–110 kb (Fig. 3C), and they covered ∼30.5–46.3% of the *Jatropha* chromosomes. Great differences were observed in the TAD regions in both the g-bud vs m-bud and m-leaf vs m-bud comparisons (Fig. 2A and C), implying that chromatin architecture also differs among different phenotypes or tissues in *Jatropha*. In rice, the formation of TADs may be relevant to histone modifications and gene transcription; the density of protein-coding genes is much lower in TAD interior regions than in the boundary regions [43, 44]. In *Jatropha*, gene density was significantly higher in the TAD boundary regions than in the TAD interior regions in inflorescence buds (m-bud and g-bud groups), similar to the case in rice, but no differences between leaves and buds (m-leaf and m-bud groups) were observed (Fig. 3A and B), suggesting that TAD features vary among different sexual phenotypes of *Jatropha*.

The chromatin loop is a fine chromatin structure that brings distant DNA elements and their target genes into close proximity, facilitating transcriptional activation [41]. We detected 2,221, 2,409, and 371 chromatin loops from the contact matrices of the m-bud, g-bud, and m-leaf samples, respectively (Additional Table S3), using the HiCCUPS algorithm in the Juicer pipeline [66]. These chromatin loops were confirmed using the aggregate peak analysis (APA) algorithm in the Juicer pipeline (Additional Fig. S7) [66]. Differential chromatin loops were detected in the g-bud vs m-bud and m-leaf vs m-bud comparisons (Additional Table S4), suggesting that chromatin loops are also varied; this variation may be relevant to different phenotypes or tissues.

Chromatin architecture plays important roles in the regulation of gene expression during various cellular processes [65]. We monitored obvious local chromatin architecture alterations in A/B compartments, TADs, and chromatin loops across the m-bud, g-bud, and m-leaf samples (Fig. 2, Additional Fig. S6, and Additional Table S4). The results implied that chromatin organization is intimately associated with different sexual phenotypes and organ morphologies in *Jatropha*.

### Differential contacts and differentially expressed genes between monoecious and gynoecious inflorescence buds

**Figure 4:**
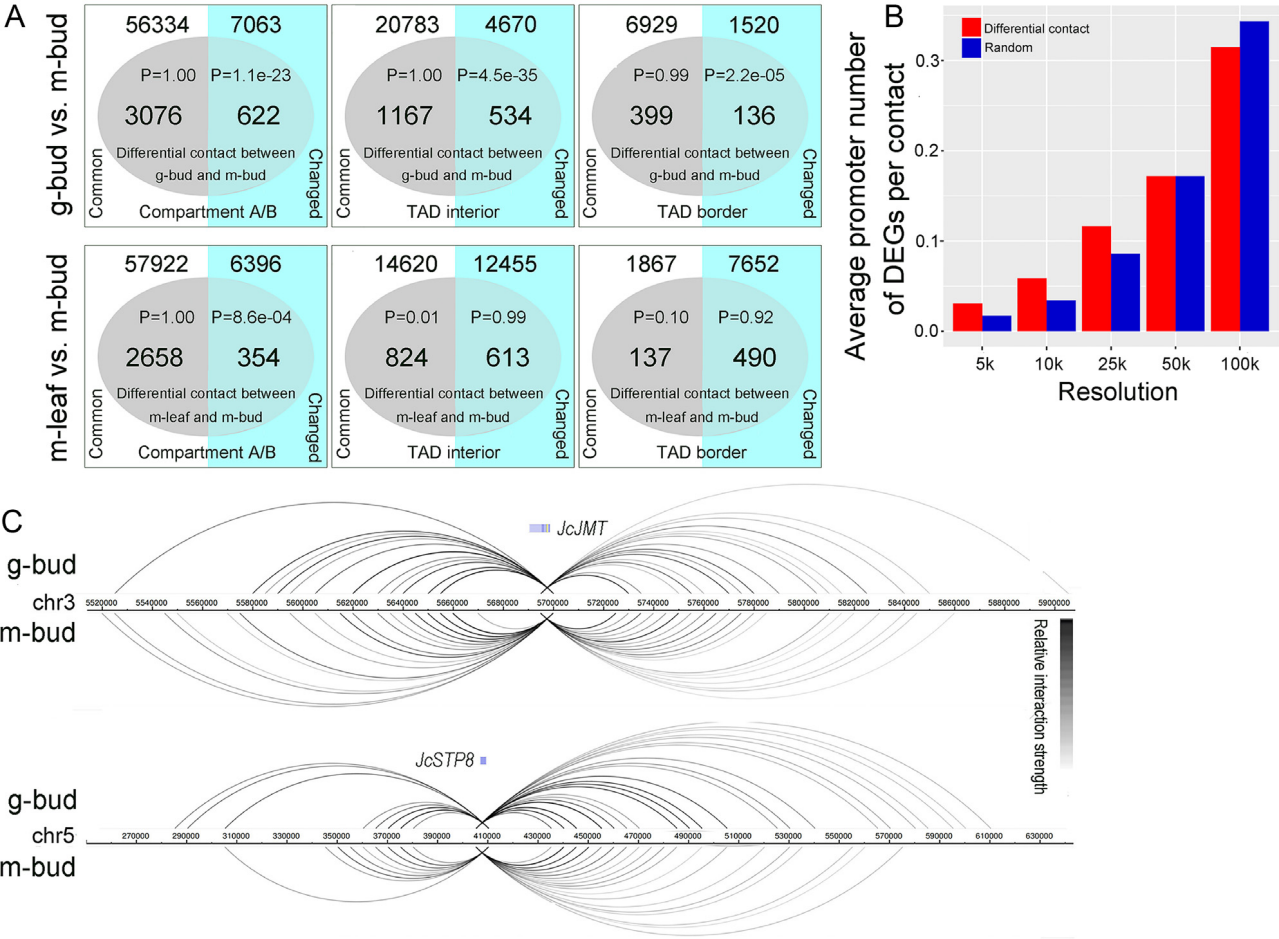
Differential contacts are relevant to gene transcription. (A) Enrichment analysis of the differential contacts in chromatin architecture regions. A hypergeometric distribution test was performed with the phyper function in R software. (B) Densities of DEG promoters in differential contact regions between the g-bud and m-bud samples. (C) Genomic interaction profiles of the *JcJMT* and *JcSTP8* genes in the m-bud and g-bud samples. The label “m-bud” indicates monoecious inflorescence bud samples, the label “g-bud” indicates gynoecious inflorescence bud samples, and the label “m-leaf” indicates monoecious leaf samples.

To further investigate the function of chromatin architecture in sex differentiation, we detected differences in chromatin interactions between gynoecious and monoecious inflorescence buds using the HiCcompare package [67]. A total of 2,425–3,036 differential contacts were identified with a false discovery rate (FDR) of ≤0.05 at 5–100 kb resolution (Additional Table S5). The differential contacts between g-bud and m-bud samples preferentially occurred in the altered chromatin architecture regions, while those between m-leaf and m-bud samples were enriched only in the changed A/B compartment regions (Fig. 4A). These findings imply that the differential contacts are relevant to chromatin architecture alterations during *Jatropha* sex differentiation.

In addition, we identified 1,165 DEGs between gynoecious and monoecious inflorescence buds with an FDR of ≤0.05 and a fold change ≥2.0 using our published transcriptome data (Additional Fig. S8, Additional Tables S6 and S7) [68]. Gene Ontology (GO) and KEGG analyses showed that the “reproductive process” (GO: 0022414) and “plant hormone signal transduction” (ath04075) functional categories were enriched for the DEGs (Additional Figs S9 and S10 and Table S8). We investigated the relationship between DEG distribution and differential contact regions and found that the promoters of 241 DEGs overlapped with 223 differential contact regions at both 5 and 10 kb resolutions, implying that these genes may be regulated by DNA regulatory elements located in corresponding differential contact regions (Additional Table S9). The promoter density of the DEGs was obviously higher in the differential contact regions than in the other regions (background) at 5, 10, and 25 kb resolutions, respectively (Fig. 3B), suggesting that gene transcription is linked to the differential contacts. These results were coincident with the findings that the differential contacts were associated with chromatin architecture alterations between gynoecious and monoecious inflorescence buds (Fig. 4A).

Moreover, we identified 12 genes from the 241 differentially expressed genes (DEGs) located in the differential contact regions that are homologous to *Arabidopsis* genes involved in flower development or biosynthesis of phytohormones associated with sex differentiation in *Jatropha*. The 12 genes included *Jatropha feronia* (*JcFER, jc003891*), *gibberellin 2-oxidase 8* (*JcGA2OX8, jc021138*), *increase in bonsai methylation 1* (*JcIBM1, jc006371*), *isopentenyltransferase 5* (*JcIPT5, jc020647*), *jasmonic acid carboxyl methyltransferase* (*JcJMT, jc008699*), *matrix metalloproteinase* (*JcMMP, jc004196*), *receptor-like kinase in flowers 1* (*RKF1, jc023149*), *sugar transport protein 8* (*JcSTP8, jc002715*), *terpene synthase 21* (*JcTPS21, jc019906*), *tRNA isopentenyltransferase 2* (*JcIPT2, jc006165*), *ubiquitin ligase complex subunit 1* (*JcULCS1, jc023230*), and *Zusammen-CA-enhanced 1* (*JcZCE1, jc021698*) (Additional Table S10). These genes displayed different genomic interaction patterns between gynoecious and monoecious inflorescence buds (Fig. 4C and Additional Fig. S11), suggesting that their expression may be regulated by corresponding regulatory elements during sex differentiation. For example, *JcSTP8* and *JcJMT* had different interaction loci at 5 kb resolution between the m-bud and g-bud samples, which may have helped promote the expression of *JcSTP8* or inhibit the expression of *JcJMT* (Fig. 4C and Additional Table S7). In *Arabidopsis*, STP8 contributes to the uptake of glucose during pollen development and pollen tube growth [69, 70], and JMT catalyses the formation of methyljasmonate from JA [71]. *JcSTP8* and *JcJMT*, together with the other genes identified in this study, could participate in *Jatropha* sex differentiation, during which chromatin organization may regulate their transcription.

### Distribution of DEGs and co-expressed genes in chromatin architecture units

**Figure 5:**
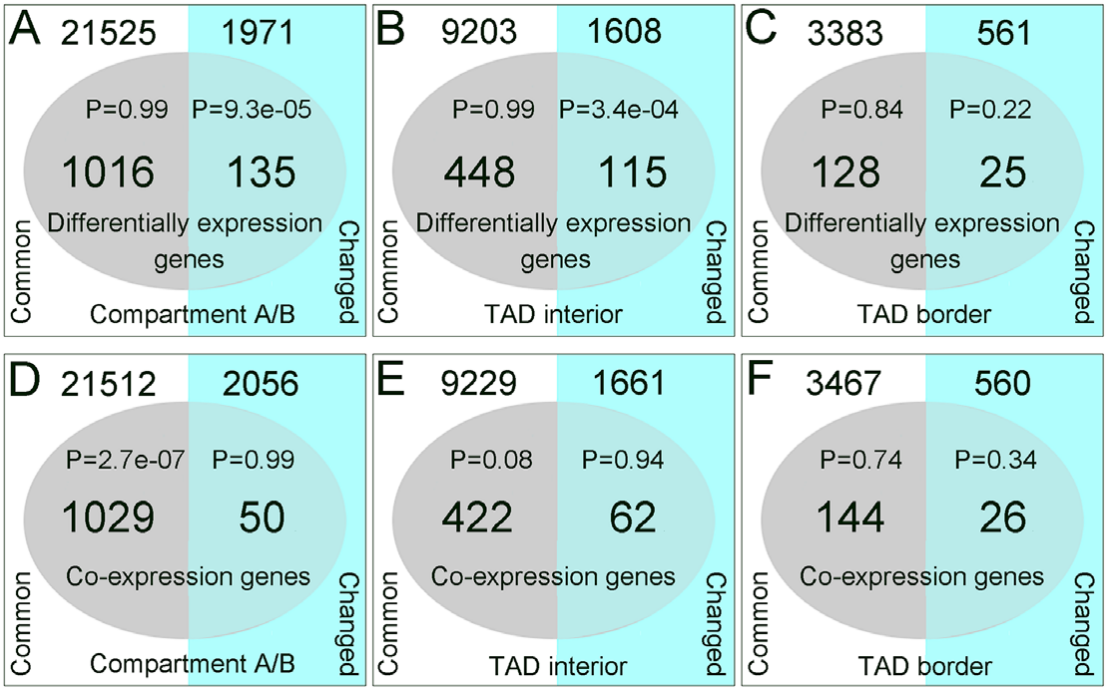
Enrichment analysis of the DEGs and the co-expressed genes in chromatin architecture regions. (A–C) Enrichment analysis of the DEGs in A/B compartments, TAD interiors, and TAD boundaries, respectively, in common and changed regions. (D–F) Enrichment analysis of the co-expressed genes in A/B compartments, TAD interiors, and TAD boundaries, respectively, in common and changed regions. A hypergeometric distribution test was performed with the phyper function in R software.

We performed co-expression analyses of the transcriptome data for different *Jatropha* phenotypes and tissues using the WGCNA package (version 1.46) (Additional Table S11) [72–74] and detected 3 modules, MEgreen, MEdarkgreen, and MElightcyan (Additional Fig. S12, Additional Tables S12 and S13). The genes in these modules were enriched for the “reproductive process” function category, implying that they may be related to sex differentiation in *Jatropha*. We computed the distribution of DEGs and co-expressed genes in common and different regions of the chromatin architecture between g-bud and m-bud samples, respectively. The results showed that DEGs were significantly enriched in the altered A/B compartment and TAD regions, but co-expressed genes were not (Fig. 5), suggesting that the DEGs were associated with chromatin architecture alteration and that the co-expressed genes are irrelevant to chromatin organization during sex differentiation in *Jatropha*.

## Discussion

Chromatin organization is an important factor regulating gene transcription in many cellular processes, and dynamic alterations in chromatin architecture play vital roles in responses to environmental stimuli [43, 65, 75–77]. The 3D structure of each chromosome contains 3 hierarchical functional substructure units: A/B compartments, TADs, and chromatin loops [36–39]. In *Jatropha*, the same hierarchical chromatin substructures were found in the nucleus with the Hi-C approach, as they have been in *Arabidopsis* and several crop species [43, 44, 65], implying that these chromatin architectures are widely present in plants. The structural features of TADs are well conserved among species, cell types, and tissues in mammals [36, 40, 78] but not in plants; the lack of conservation in plants might be because of the absence of the CTCF protein that is highly enriched at TAD borders in mammalian systems [36, 79]. This non-conservation of chromatin architecture may contribute to adaptations of plants in response to various environmental conditions. The observation of dynamic alterations in chromatin architecture across the m-bud, g-bud, and m-leaf samples suggests that chromatin organization is associated with different sexual phenotypes or organ morphologies in *Jatropha*.

Through examination of both DEGs and differential contacts between gynoecious and monoecious inflorescence buds, 12 genes involved in *Jatropha* sex differentiation were identified, the expression of which may be regulated by corresponding DNA regulatory elements. In *Arabidopsis, IBM1* encodes a histone demethylase suppressing DNA methylation and gene silencing, and the *ibm1* mutant displays developmental defects [80, 81]. *RKF1* is highly expressed in early flower primordia and during stamen development [82]. *ULCS1* encodes a WD40 repeat protein, RNA interference–mediated silencing of which produces sterile plants with pleiotropic phenotypes [83]. *TPS21* is a sesquiterpene synthase gene expressed in stigmas, anthers, and sepals, which is responsible for the formation of floral volatile sesquiterpenes [84]. *ZCE1* encodes a member of the major latex protein-like gene family that plays a role in promoting vegetative growth and delaying flowering [85]. In *Jatropha*, the expression of *JcIBM1, JcRKF1, JcULCS1, JcTPS21, JcZCE1*, and *JcSTP8* was upregulated in gynoecious inflorescence buds (Additional Table S6). *MMP* is a member of the matrix metalloproteinase gene family, and the *Arabidopsis mmp-1* mutant displays late flowering and early senescence phenotypes [86]. *FER* encodes a plasma membrane receptor protein kinase that regulates reproductive growth [52]. *GA2OX8* encodes a GA 2-oxidase that participates in the GA biosynthetic process [87]. The expression of *JcMMP, JcFER, JcGA2OX8*, and *JcJMT* was downregulated in gynoecious inflorescence buds (Additional Table S6) in the present study. Moreover, in *Arabidopsis, IPT2* and *IPT5* encode CK synthases that catalyse the first step in CK biosynthesis [88]. In *Arabidopsis* ATP/ADP IPTs (IPT1 and IPT3–IPT8) are responsible for isopentenyladenine- and *trans*-zeatin (*t*Z)-type CK synthesis, while tRNA IPTs (IPT2 and IPT9) are responsible for *cis*-zeatin (*c*Z)-type CK synthesis [88]. In *Jatropha, JcIPT2* was upregulated in gynoecious inflorescence buds, while *JcIPT5* was downregulated, suggesting that different types of CKs may play different roles in *Jatropha* sex differentiation even though exogenous CK treatment has been found to improve the production of female flowers [28–30]. These genes displayed different genomic interaction patterns between gynoecious and monoecious inflorescence buds (Fig. 4C and Additional Fig. S11), suggesting that their transcriptional activity may be associated with chromatin organization during sex differentiation in *Jatropha*.

## Conclusions

In this study, we obtained a chromosome-level *de novo* assembly of the *Jatropha* genome using PacBio sequencing combined with Hi-C technology. Based on this high-quality reference genome, we first revealed the features of chromatin architecture in perennial woody plants and investigated the possible function of chromatin organization in sex differentiation in *Jatropha*, which will facilitate understanding of the regulatory mechanisms of sex determination in higher plants.

## Methods

### Plant materials

Two-year-old gynoecious and monoecious *Jatropha curcas* plants were grown in the field at the Xishuangbanna Tropical Botanical Garden of the Chinese Academy of Sciences, Yunnan Province, China. Inflorescence buds and leaves from gynoecious and monoecious plants were fixed for Hi-C library construction. Leaves of monoecious plants were frozen for PacBio sequencing. Two biological replicates per sample were generated for Hi-C library construction.

### PacBio sequencing and *de novo* assembly

PacBio sequencing was performed on a PacBio Sequel sequencer by Novogene Bioinformatics Technology (Beijing, China). After the polymerase reads were filtered (minReadScore = 0.8), the filtered subreads were used for first-round assembly using the FALCON package, version 0.3.0 (Falcon, RRID:SCR_016089) with the following parameters: length cutoff = 1000, seed coverage = 35, and length cutoff pre-assembly = 11,000 [56]. The contig sequences produced were corrected with PacBio sequencing data using the arrow algorithm in PacBio SMRT Link (version 5.1.0) [89]. Along with the Hi-C sequencing data, the contig sequences were then integrated into a candidate chromosome-scale assembly using a 3D DNA pipeline [54]. The candidate assembly was further corrected for the final genome sequences using Juicebox Assembly Tools (Version 1.8.9) [57].

### Genome annotation and quality evaluation

After masking repetitive sequences based on a custom repeat library with the RepeatModeler package (RepeatModeler, RRID:SCR_015027) [90], the assembly of monoecious *Jatropha* was annotated using the MAKER genome annotation pipeline, version 2.31.10 (MAKER, RRID:SCR_005309) [58, 59]. Both transcript and protein sequences were used for *ab initio* gene prediction. The transcript sequences were *de novo* assembled with our previous transcriptome sequencing data (SRP092157) and NCBI RefSeq *Jatropha* transcript data using Trinity, version 2.2.0 (Trinity, RRID:SCR_013048), with the default parameters [91, 92]. The protein sequences were from the Ensembl Plants database (Ensembl Plants, RRID:SCR_008680) [93]. The SNAP and AUGUSTUS programs in the MAKER pipeline were used to train the gene prediction model [94, 95]. A detailed description of the MAKER pipeline is provided on the MAKER Wiki page [96]. The AED algorithm was used for assembly annotation [60]. The QUAST-LG (QUAST-LG, RRID:SCR_001228), BUSCO version 3.0 (BUSCO, RRID:SCR_015008), mummer version 4.0 (mummer, RRID:SCR_001200), and MCScanX packages were used to assess assembly completeness and contiguity [61–64]. Single-base substitutions and short insertions and deletions in the assembly were estimated with PacBio long-read alignments using the arrow algorithm in PacBio SMRT Link (version 5.1.0). A visual Hi-C–based chromatin interaction map approach was used to assess misassemblies, such as structural errors, using Juicebox Assembly Tools (version 1.8.9) [57].

### Hi-C library preparation

The Hi-C protocol was adapted for library construction as previously described [97]. Plant materials were fixed with 1% formaldehyde solution at room temperature for 30 min in a vacuum. Then, 2.5 M glycine was added to quench the cross-linking reaction. Approximately 0.5 g of fixed tissue was ground with liquid nitrogen for DNA isolation. The extracted nuclei were resuspended with 0.5% SDS and incubated at 62°C for 10 min. Then, 10% Triton X-100 was added, and the samples were incubated at 37°C for 15 min. The denatured DNA was digested with the 4-cutter restriction enzyme DpnII at 37°C for 4 h. The DpnII enzyme was inactivated at 62°C for 20 min. Next, the digested DNA was blunt-ended by filling in of nucleotides with the Klenow enzyme at 37°C for 30 min. The proximal chromatin DNA was religated with T4 DNA ligase at room temperature for 4 h. After centrifugation at 1500 × *g* for 3 min, the reaction mixture was resuspended with SDS buffer (50 mM Tris-HCl, 1% SDS, 10 mM EDTA, pH 8.0), proteinase K was added, and the mixture was incubated at 55°C for 30 min. Formaldehyde cross-linking of nuclear complexes was reversed by addition of 30 μL of 5 M NaCl and incubation at 65°C overnight. Subsequent manipulations were carried out as previously described [97]. DNA was reverse crosslinked, purified, and fragmented by sonication on a Covaris sonicator. Biotin labelled DNA was pulled down on Streptavidin Dynabeads. After DNA repair and 3’ A addition, adaptor was added. Diluted DNA on Dynabeads was used for PCR amplifications to produce similar amounts of DNA for sequencing on the Illumina HiSeq X10 platform (PE 2 × 150 bp reads).

### Hi-C data analysis pipeline

Analysis of the Hi-C sequencing data was performed using the Juicer pipeline (Juicer, RRID:SCR_017226) [66]. Duplicate and near-duplicate reads mapped to the same restriction fragment were removed and then filtered with mapping quality scores. The contact matrices were normalized at different resolutions. Eigenvectors were identified with the eigenvector algorithm, the sign of which indicated whether the reads were in compartment A or compartment B; TADs were identified with the arrowhead algorithm; and chromatin loops were identified with the HiCCUPS algorithm. The aggregate enrichment of putative peaks in contact matrices was validated with the APA algorithm as described previously [38]. Differential chromatin contacts between the contact matrices were identified using the HiCcompare R package (version 1.8.0) [67]. All Hi-C maps were generated using Juicebox Assembly Tools (version 1.8.9) [57]. Correlation analysis was performed using the corrplot R package (Version 0.85) [98].

### Analysis of DEGs and co-expressed genes

Our previous transcriptome data were reanalysed to examine DEGs between monoecious and gynoecious inflorescence buds. The sequencing reads were mapped to the new *Jatropha* reference genome using the Subread package, version 1.6.2 (Subread, RRID:SCR_009803), with the default parameters [99, 100]. DEGs with an FDR of ≤0.05 and an expression fold change ≥2.0 were identified using the edgeR package (edgeR, RRID:SCR_012802) [101]. Co-expressed gene analysis was performed using the WGCNA R package, version 1.46 (RRID:SCR_003302) [74]. The expression counts of all samples were log_2_(x+1)-transformed, and batch effects were removed using the ComBat function in the SVA package, version 3.34.0 (SVA, RRID:SCR_002155) [102]. GO and KEGG annotation were performed with the Database for Annotation, Visualization and Integrated Discovery (DAVID), version 6.8 (DAVID, RRID:SCR_001881) [103].

## Availability of Supporting Data and Materials

All high-throughput sequencing reads and the assembly presented in the manuscript have been submitted to the China National GeneBank (CNGB) Nucleotide Sequence Archive (CNSA) under accession number CNP0000449. Raw data are also available and clustered together under NCBI bioproject PRJNA415534. In this study, the data for CNR0106032–CNR0106034 are found under CNGB CNSA accession NCNP0000603; the data from SRR10076311–SRR10076316, SRR10076310, and SRR10076325 are found under NCBI accession number SRP220547; the data for SRR1565783–SRR1565786, SRR1565789–SRR1565790, and SRR1565797–SRR1565798 are found under NCBI accession SRP046221; the data for SRR4473569, SRR4473570, SRR4473575, SRR4473565, SRR4473571, and SRR4473572 found from the NCBI accession SRP092157; and the data for SRR6227301, SRR6227302, SRR6227305, SRR6227306, SRR6227308, and SRR6227312 are all found from the NCBI accession SRP122257. All supporting data and materials are also available in the *GigaScience* GigaDB database [104].

## Additional Files

**Figure S1:** Distribution of PacBio subread lengths.

**Figure S2:** AED score of our *Jatropha* assembly annotation.

**Figure S3:** Synteny analysis between our assembly and the published *Jatropha* assemblies based on gene sequences.

**Figure S4:** Correlation analysis of Hi-C contact matrices between biological replicates. The label “m-bud” indicates monoecious inflorescence bud samples, the label “g-bud” indicates gynoecious inflorescence bud samples, and the label “m-leaf” indicates monoecious leaf samples. The number indicates the correlation coefficient.

**Figure S5:** Hi-C contact maps of the m-bud, g-bud, and m-leaf samples. (A) Genome-wide Hi-C contact maps. (B) Hi-C contact maps of chromosome 1 at 25 kb resolution (observed/expected). The blue area indicates the A compartment region, and the brown area indicates the B compartment region. The labels “m-bud,” “g-bud,” and “m-leaf” indicate the same samples shown in Additional Figure S4.

**Figure S6:** Comparison of the A/B compartments in all chromosomes among the m-bud, g-bud, and m-leaf samples. The black boxes indicate the changed A/B compartment regions, the blue area indicates the A compartment region, and the brown area indicates the B compartment region. The labels “m-bud,” “g-bud,” and “m-leaf” indicate the same samples shown in Additional Figure S4.

**Figure S7:** APA of Hi-C contact matrices across m-bud, g-bud, and m-leaf samples. The labels “m-bud,” “g-bud,” and “m-leaf” indicate the same samples shown in Additional Figure S4.

**Figure S8:** DEGs identified from the g-bud vs m-bud comparison. The blue lines indicate genes with a 2-fold expression change; the red points indicate significant DEGs with FDRs <0.05. FC, fold change; CPM, counts per million mapped reads.

**Figure S9:** GO enrichment analysis of the DEGs. The asterisk indicates the “reproductive process” function category.

**Figure S10:** KEGG enrichment analysis of the DEGs.

**Figure S11:** Genomic interaction profiles of 10 DEGs in the m-bud and g-bud samples. The labels “m-bud” and “g-bud” indicate the same samples shown in Additional Figure S4.

**Figure S12:** Co-expression analysis and GO annotation. (A) Correlation analysis between modules and biological traits (phenotype and tissue) was performed using WGCNA. The right coloured bar indicates the correlation coefficient. The numbers in each coloured cell indicate the correlation coefficient and the corresponding *P*-value (numbers in brackets), calculated using the WGCNA package. The asterisks indicate the MEgreen, MEdarkgreen, and MElightcyan modules. (B) GO enrichment analysis of the co-expressed genes in the MEgreen, MEdarkgreen, and MElightcyan modules. The asterisk indicates the “reproductive process” function category.

**Table S1:** Statistics of our assembly and the published *Jatropha* assemblies.

**Table S2:** Statistics of the Hi-C data of the m-bud, g-bud, and m-leaf samples.

**Table S3:** Chromatin loops identified from the m-bud, g-bud, and m-leaf contact matrices.

**Table S4:** Differential chromatin loops in the g-bud vs m-bud and m-leaf vs m-bud comparisons.

**Table S5:** Differential chromatin contacts at 5 kb resolution in the g-bud vs m-bud and m-leaf vs m-bud comparisons.

**Table S6:** DEGs identified in the g-bud vs m-bud comparison.

**Table S7:** Results of transcriptome comparison between g-bud and m-bud samples.

**Table S8:** List of enriched genes annotated with GO and KEGG analyses.

**Table S9:** DEGs overlapping with differential contact regions at 5 and 10 kb resolutions between g-bud and m-bud samples.

**Table S10:** Twelve DEGs that might be involved in sex differentiation located in differential contact regions between the g-bud and m-bud samples.

**Table S11:** List of transcriptome data for co-expression analysis.

**Table S12:** Gene list for the MEgreen, MEdarkgreen, and MElightcyan modules.

**Table S13:** GO enrichment analysis of the co-expressed genes in the MEgreen, MEdarkgreen, and MElightcyan modules.

giaa009_GIGA-D-19-00223_Original_Submission

giaa009_GIGA-D-19-00223_Revision_1

giaa009_Response_to_Reviewer_Comments_Original_Submission

giaa009_Reviewer_1_Report_Original_Submission Paris Veltsos -- 7/16/2019 Reviewed

giaa009_Reviewer_2_Report_Original_Submission Siam Popluechai -- 7/29/2019 Reviewed

giaa009_Reviewer_3_Report_Original_Submission Jian-Feng Mao, Ph.D. -- 8/11/2019 Reviewed

giaa009_Reviewer_3_Report_Revision_1 Jian-Feng Mao, Ph.D. -- 12/7/2019 Reviewed

giaa009_Supplemental_Figures_and_Tables

## Abbreviations

ACS1: 1-aminocyclopropane-1 carboxylic acid synthase; ADP: adenosine diphosphate; AED: annotation edit distance; APA: aggregate peak analysis; ATP: adenosine triphosphate; bp: base pairs; BR: brassinosteroid; BUSCO: Benchmarking Universal Single-Copy Orthologs; CK: cytokinin; DEG: differentially expressed gene; EDTA: ethylenediaminetetraacetic acid; ETH: ethylene; FDR: false discovery rate; FER: feronia; GA: gibberellin; GA2OX8: gibberellin 2-oxidase 8; Gb: gigabase pairs; GO: Gene Ontology; Hi-C: genome-wide chromosome conformation capture; ids1: indeterminate spikelet1; IBM1: increase in bonsai methylation 1; IPT2: tRNA isopentenyltransferase 2; IPT5: isopentenyltransferase 5; JA: jasmonic acid; JMT: jasmonic acid carboxyl methyltransferase; kb: kilobase pairs; KEGG: Kyoto Encyclopedia of Genes and Genomes; Mb: megabase pairs; MMP: matrix metalloproteinase; na1: nana plant1; NCBI: National Center for Biotechnology Information; PacBio: Pacific Biosciences; RKF1: receptor-like kinase in flowers 1; SDS: sodium dodecyl sulfate; SMRT: single-molecule real-time; STP8: sugar transport protein 8; TAD: topologically associated domain; TPS21: terpene synthase 21; tRNA: transfer RNA; ts2: tasselseed2; ULCS1: ubiquitin ligase complex subunit 1; ZCE1: (Zusammen-CA)-enhanced 1.

## Competing Interests

The authors declare that they have no competing interests.

## Funding

This work was supported by the National Natural Science Foundation of China (31670612, 31971628, 31870291, 31300568, 31370595, and 31571347), Natural Science Foundation of Yunnan Province (2016FB033), the Programme of the Chinese Academy of Sciences (kfj-brsn-2018-6-008 and 2017XTBG-T02), and the Guangdong Science and Technology Department (2016A030313642).

## Authors' Contributions

L.N., M.-S.C., C.H., and Z.-F.X. designed the study and wrote the manuscript. L.N. performed the Hi-C experiments. M.-L.Z., C.X., B.-Z.P., Q.F., Y.-B.T., and H.H. carried out additional experiments. M.-S.C. and L.N. analysed and interpreted the data. All authors reviewed the final manuscript.
