# Supplementary material for: De novo genome assembly and Hi-C analysis reveal an association between chromatin architecture alterations and sex differentiation in the woody plant Jatropha curcas
Source: Gigascience. 2020 Feb 12;9(2):giaa009. doi: 10.1093/gigascience/giaa009 (PMC7014976; doi:10.1093/gigascience/giaa009)
Supplement: giaa009_GIGA-D-19-00223_Original_Submission [file giaa009_giga-d-19-00223_original_submission.pdf]

GigaScience

Chromatin architecture alterations are associated with sex differentiation in the woody plant Jatropha curcas, based on an improved genome assembly and comparative transcriptome analysis between monoecious and gynoecious inflorescence buds

--Manuscript Draft--

**Manuscript Number:**	GIGA-D-19-00223
**Full Title:**	Chromatin architecture alterations are associated with sex differentiation in the woody plant Jatropha curcas, based on an improved genome assembly and comparative transcriptome analysis between monoecious and gynoecious inflorescence buds
**Article Type:**	Research

Funding Information:

Programme of the Chinese Academy of Sciences (2017XTBG-T02)	Dr. Zeng-Fu Xu
National Natural Science Foundation of China (31670612)	Dr. Mao-Sheng Chen
National Natural Science Foundation of China (31870291)	Dr. Bang-Zhen Pan
National Natural Science Foundation of China (31300568)	Dr. Qian-Tang Fu
National Natural Science Foundation of China (31370595)	Dr. Zeng-Fu Xu
National Natural Science Foundation of China (31571347)	Dr. Chunhui Hou
Programme of the Chinese Academy of Sciences (kfj-brsn-2018-6-008)	Dr. Zeng-Fu Xu
Guangdong Science and Technology Department (2016A030313642)	Dr. Chunhui Hou

Abstract:

Background: Chromatin architecture is an essential factor in regulating gene transcription in different cell types and developmental phases. However, studies of chromatin architecture in perennial woody plants and the function of chromatin organization in sex determination have not been reported until now.

Results: Here, we report a chromosome-scale de novo genome assembly of the woody plant Jatropha curcas with a total length of 379.5 Mb and scaffold N50 of 30.7 Mb using PacBio long-reads combined with genome-wide chromosome conformation capture (Hi-C) technology. Based on this high-quality reference genome, we then detected features of chromatin architecture in the monoecious and gynoecious inflorescence buds of Jatropha. A striking difference in chromatin architecture between monoecious and gynoecious Jatropha suggests that chromatin organization is associated with the process of sex differentiation. Moreover, we found that the promoter density of differentially expressed genes (DEGs) is obviously higher in differential contact regions between monoecious and gynoecious inflorescence buds than in other regions, indicating that the expression of DEGs is intimately linked to differential contacts resulting from alteration of chromatin organization. Among these DEGs, twelve genes are relevant to flower development or hormone synthesis, which may participate in the process of sex differentiation in Jatropha. All of these genes show different genomic interaction patterns between monoecious and gynoecious Jatropha.

Conclusions: For the first time, we revealed the features of chromatin architecture in perennial woody plants and investigated the possible function of chromatin organization in the sex differentiation of Jatropha; these findings will facilitate understanding of the regulatory mechanisms of sex determination in higher plants.

Powered by Editorial Manager® and ProduXion Manager® from Aries Systems Corporation

Corresponding Author:	Zeng-Fu Xu, Ph.D.<br>Xishuangbanna Tropical Botanical Garden<br>Menglun, Mengla, Yunnan CHINA
Corresponding Author Secondary Information:	
Corresponding Author's Institution:	Xishuangbanna Tropical Botanical Garden
Corresponding Author's Secondary Institution:	
First Author:	Mao-Sheng Chen
First Author Secondary Information:	
Order of Authors:	Mao-Sheng Chen
	Longjian Niu
	Mei-Li Zhao
	Chuanjia Xu
	Bang-Zhen Pan
	Qian-Tang Fu
	Yan-Bin Tao
	Hui-Ying He
	Chunhui Hou
	Zeng-Fu Xu, Ph.D.
Order of Authors Secondary Information:	
Additional Information:	
Question	Response
Are you submitting this manuscript to a special series or article collection?	No
**Experimental design and statistics**<br><br>Full details of the experimental design and statistical methods used should be given in the Methods section, as detailed in our Minimum Standards Reporting Checklist. Information essential to interpreting the data presented should be made available in the figure legends.<br><br>Have you included all the information requested in your manuscript?	Yes
**Resources**<br><br>A description of all resources used, including antibodies, cell lines, animals and software tools, with enough	Yes

information to allow them to be uniquely identified, should be included in the Methods section. Authors are strongly encouraged to cite Research Resource Identifiers (RRIDs) for antibodies, model organisms and tools, where possible.<br><br>Have you included the information requested as detailed in our Minimum Standards Reporting Checklist?	
**Availability of data and materials**<br><br>All datasets and code on which the conclusions of the paper rely must be either included in your submission or deposited in publicly available repositories (where available and ethically appropriate), referencing such data using a unique identifier in the references and in the "Availability of Data and Materials" section of your manuscript.<br><br>Have you have met the above requirement as detailed in our Minimum Standards Reporting Checklist?	Yes

Powered by Editorial Manager® and ProduXion Manager® from Aries Systems Corporation

Manuscript Click here to download Manuscript Manuscript_17June.docx

Chromatin architecture alterations are associated with sex differentiation in the woody plant *Jatropha curcas*, based on an improved genome assembly and comparative transcriptome analysis between monoecious and gynoecious inflorescence buds

Mao-Sheng Chen[1, †], Longjian Niu[2, 4, †, *], Mei-Li Zhao[1, 3], Chuanjia Xu[1, 3], Bang-Zhen Pan[1], Qian-Tang Fu[1], Yan-Bin Tao[1], Hui-Ying He[1], Chunhui Hou[2, *], Zeng-Fu Xu[1, *]

[1] CAS Key Laboratory of Tropical Plant Resources and Sustainable Use, Xishuangbanna Tropical Botanical Garden, Core Botanical Gardens, Chinese Academy of Sciences, Menglun, Mengla, Yunnan 666303, China

[2] Department of Biology, Southern University of Science and Technology, Shenzhen, Guangdong 518055, China

[3] College of Life Sciences, University of Chinese Academy of Sciences, Beijing 100049, China

[4] Department of Biology, Nankai University, Tianjin, 660885, China

* Correspondence: niulongjian@126.com; hou.ch@sustc.edu.cn; zfxu@xtbg.ac.cn

† Mao-Sheng Chen and Longjian Niu contributed equally to this work

Mao-Sheng Chen,	E-mail: chenms@xtbg.org.cn
Longjian Niu,	E-mail: niulongjian@126.com
Mei-Li Zhao,	E-mail: zhaomeili@xtbg.ac.cn
Chuanjia Xu,	E-mail: xuchuanjia@xtbg.ac.cn
Bang-Zhen Pan,	E-mail: pbz@xtbg.org.cn
Qiantang Fu,	E-mail: qtfu2002@163.com
Yan-Bin Tao,	E-mail: taoyanbin@xtbg.ac.cn
Hui-Ying He,	E-mail: hhy@xtbg.org.cn
Chunhui Hou,	E-mail: houch@sustech.edu.cn
Zeng-Fu Xu,	E-mail: zfxu@xtbg.ac.cn

Abstract

Background: Chromatin architecture is an essential factor in regulating gene transcription in different cell types and developmental phases. However, studies of chromatin architecture in perennial woody plants and the function of chromatin organization in sex determination have not been reported until now.

Results: Here, we report a chromosome-scale *de novo* genome assembly of the woody plant *Jatropha curcas* with a total length of 379.5 Mb and scaffold N50 of 30.7 Mb using PacBio long-reads combined with genome-wide chromosome conformation capture (Hi-C) technology. Based on this high-quality reference genome, we then detected features of chromatin architecture in the monoecious and gynoecious inflorescence buds of *Jatropha*. A striking difference in chromatin architecture between monoecious and gynoecious *Jatropha* suggests that chromatin organization is associated with the process of sex differentiation. Moreover, we found that the promoter density of differentially expressed genes (DEGs) is obviously higher in differential contact regions between monoecious and gynoecious inflorescence buds than in other regions, indicating that the expression of DEGs is intimately linked to differential contacts resulting from alteration of chromatin organization. Among these DEGs, twelve genes are relevant to flower development or hormone synthesis, which may participate in the process of sex differentiation in *Jatropha*. All of these genes show different genomic interaction patterns between monoecious and gynoecious *Jatropha*.

Conclusions: For the first time, we revealed the features of chromatin architecture in perennial woody plants and investigated the possible function of chromatin organization in the sex differentiation of *Jatropha*; these findings will facilitate understanding of the regulatory mechanisms of sex determination in higher plants.

Keywords: high-quality genome, Hi-C, sex determination, chromatin architecture, *Jatropha*

Introduction

Flowering plants have extremely diverse reproductive systems that are controlled by both genetic factors and environmental cues [1]. For optimal outcrossing and efficient resource allocation, approximately 10% of angiosperm species have evolved reproductive systems with unisexual flower, in which the male and female reproductive organs are physically separated; these plant taxa are termed dioecious and monoecious [2, 3]. Sex determination has evolved independently multiple times and various regulatory mechanisms control the process [4-6]. In the sex determination of cucumber, the *femaleness* (*F*) locus controls the degree of femaleness, the *androecious* (*A*) locus promotes maleness and the *andromonoecious* (*M*) locus is responsible for the selective abortion of stamens [7]. The *F* locus has been linked to the 1-aminocyclopropane-1 carboxylic acid synthase (*CsACS1*) gene, which occurs as a single copy in monoecious lines but is duplicated in gynoecious lines [8, 9]; the *M* locus has been linked to the *CsACS2* gene, and a conserved residue conversion (Gly33Cys) in *CsACS2* causes the generation of bisexual flowers in cucumber [10]. The *andromonoecious* (*a*) and *gynoecious* (*g*) loci control sex determination in melon [11]. The *a* locus has been linked to the *GmACS-7* gene, and loss-of-function of *GmACS-7* causes male organ development, generating andromonoecious plants [12]; the *g* locus encodes a repressor of carpel development, CmWIP1, the activation of which causes a transition from male to female flowers in gynoecious plants [13]. In addition, CmACS-11 inhibits the expression of *CmWIP1*, and loss-of-function of *CmACS-11* results in a transition from monoecious to androecious individuals [14]. In *Diospyros*, an autosomal *MeGI* gene regulates anther fertility, and a Y-chromosome *OGI* gene encodes a small RNA that suppresses the expression of *MeGI*, resulting in the generation of androecious individuals [15]. In maize, the *tasselseed1* (*ts1*) gene encodes a lipoxygenase involved in jasmonic acid (JA) biosynthesis, and the *ts1* mutant has defective stamen development because of a low JA concentration [16]. *tasselseed2* (*ts2*) encodes a short-chain alcohol dehydrogenase and is required for the arrest of pistil primordium development [17]. *tasselseed4* (*ts4*) encodes a miR172

microRNA that targets *Tasselseed6* (*Ts6*) /*indeterminate spikelet1* (*ids1*), and both of *ts2* and *ts4* are essential to suppress carpel development [18]. *nana plant1* (*na1*) encodes a 5α-steroid reductase involved in brassinosteroid (BR) biosynthesis, and the *na1* mutant displays dwarf and feminized phenotypes [19]. In addition, exogenous application of auxin, BR, cytokinin (CK), ethylene (ETH), gibberellin (GA), JA, and their inhibitors also affect sexual expression in several species [7, 20-23]. Temperature, photoperiod, nutrition, drought, pH, and seasonality also influence sex differentiation, and epigenetic mechanisms are likely involved in the process [24-27]. As shown by the above results, sex differentiation is a complicated process that is mediated by both environmental and genetic factors, and the regulatory mechanisms of sex differentiation are diverse among various species.

Packing of eukaryotic chromatin forms highly ordered and hierarchical structures contributing to appropriate gene expression in different cell types and developmental phases [28, 29]. This well-ordered three-dimensional (3D) chromatin architecture is essential for gene transcription, DNA replication, and genome integrity [30-32]. According to genome-wide interaction patterns, each chromosome can be partitioned into three hierarchical chromatin structures: the A/B compartments, topologically associated domains (TADs) and chromatin loops [32-35]. The A/B compartments are associated with euchromatic (active) and heterochromatic (inactive) chromatin regions in which genomic and epigenetic features are distinct [33]. TADs are predominant chromatin structural units in which local interactions are far higher than in the boundary between two TADs [32, 36]. TAD domains can spatially confine the interaction between promoters and distal regulatory elements, facilitating the activation of transcription, and correlate well with markers of chromatin activity [31, 34]. Chromatin loops bring genes and their regulatory elements into close proximity for direct physical interactions, such as combinations of enhancers and promoters [29, 37]. Multiple enhancer-promoter combinations can share binding of common transcription factors to establish a chromatin environment in which transcription is more permissive than that created by single combination of enhancer-promoter [38].

In plants, similar chromatin architectures have been identified in the genomes of several crop species, such as rice, maize, tomato, sorghum and foxtail millet, but they are not conserved across these species, suggesting that chromatin organizations are complex and unique in higher plants [39, 40].

Jatropha curcas L., a perennial woody plant, is known as a potential biofuel plant because of its high seed oil content [41, 42]. At present, four different *Jatropha* genome assemblies have been reported [43-46], but they are insufficient to meet the requirements of chromatin architecture analysis, which requires a high-quality reference genome. *Jatropha* has two different ecotypes, monoecious and gynoecious. Monoecious plants bear male and female flowers separately on the same inflorescence; gynoecious plants bear only female flowers, and their male flowers are aborted at an early stage of inflorescence development [47, 48]. In this study, we reported a chromosome-scale *Jatropha* assembly using a combination of single-molecule, real-time (SMRT) sequencing and genome-wide chromosome conformation capture (Hi-C) technology [49, 50]. Based on this high-quality reference genome, we investigated the function of chromatin architecture in sex differentiation by comparing chromatin architectures and transcriptomes between monoecious and gynoecious inflorescence buds in *Jatropha*. Our results will facilitate the elucidation of sex determination in *Jatropha* and unveil the biological functions of chromatin architecture in higher plants.

Results

A chromosome-scale *Jatropha* genome assembly

PacBio long-read sequencing data (33.41 G) were used for *de novo* assembly of the *Jatropha* genome (Additional Figure S1). The sequence coverage was approximately 80× based on the genome size (416 Mb) estimated with flow cytometry [51]. The first round of genome assembly was performed using the FALCON package [52], and then polished using the arrow algorithm in Pacific SMRT

Link. The assembly includes 1,265 contigs with a total length of 378.3 Mb and an N50 value of 1.0 Mb (Table 1). The three-dimensional proximity information obtained via the Hi-C sequencing data was used to correct misjoins and to order and orient the contig assembly; then, the results were integrated into a candidate chromosome-scale assembly using the 3D *de novo* assembly (3D DNA) pipeline [50]. The candidate assembly was further improved by interactive correction using the Juicebox Assembly Tools [53]. The final *Jatropha* assembly (hereafter referred to as the new *Jatropha* assembly) had a total length of 379.1 Mb and an N50 value of 30.7 Mb and contained 11 complete chromosomes (each chromosome > 27.1 Mb) (Table 1). After masking of repetitive sequences, 25,817 protein-coding genes were predicted based on transcript and protein alignments using the MAKER annotation pipeline [54, 55] (Table 1). The annotation of the new *Jatropha* assembly has a high annotation edit distance (AED) score [56], suggesting a high-quality genome annotation (Additional Figure S2).

Quality evaluation of the new *Jatropha* assembly

We calculated small local errors in the new *Jatropha* assembly, such as single-base substitutions, short insertions and deletions, with PacBio long-read alignments using the arrow algorithm in PacBio SMRT Link software. The estimated error rate was 0.22% (substitutions 0.17%, insertions 0.03% and deletions 0.02%), in which most of the errors were from substitutions. However, the actual error rate should be far smaller than the estimated rate because a large number of false errors could be introduced into the genome sequence by the heterozygosity of the *Jatropha* genome. The completeness and contiguity were assessed using the QUAST-LG, BUSCO, mummer and MCScanX packages [57-60]. The BUSCO results showed that the percentage of complete genes (C) was 95.8%, the percentage of one-copy genes (S) was 93.3%, the percentage of fragmented genes (F) was 1.2%, and the percentage of missing genes (M) was 3.0% in the new *Jatropha* assembly, and these values are far better than those of the previous *Jatropha* genome assemblies (Figure 1) [43-45], suggesting that the new *Jatropha* assembly has good completeness. Comparisons of genome sequences

among the new *Jatropha* assembly and the other assemblies showed that genomic structures are similar in these assemblies, but the new *Jatropha* assembly has better completeness and contiguity than the other published assemblies [43-46] (Additional Table S1, Additional Figures S3 and S4). Moreover, we compared Hi-C interaction maps across the new *Jatropha* assembly and previous assemblies by mapping Hi-C sequencing reads to the respective reference genomes and found that the new *Jatropha* assembly displayed perfect completeness and contiguity (Figure 2, Additional Figures S5 and S6).

Features of chromatin architecture in the *Jatropha* genome

Based on the new reference genome assembly obtained in this study, we investigated the chromatin architecture of the *Jatropha* genome with the Hi-C method [33]. Three types of Hi-C libraries were constructed: "m-bud" represented Hi-C libraries from monoecious inflorescence buds, "m-leaf" represented Hi-C libraries from monoecious leaves, and "g-bud" represented Hi-C libraries from gynoecious inflorescence buds, two biological replicates were generated per library (Additional Table S2). Three two-dimensional contact maps were generated to display the chromatin architectures of the m-bud, g-bud and m-leaf samples, respectively (Figure 3), and each chromosome region was partitioned into alternating positive and negative eigenvectors representing the A/B compartments using principal component analysis (PCA) (Figure 4). The A/B compartments, which correspond to euchromatic and heterochromatic regions, are the major chromatin structural units in both animals and plants [33, 35, 61]. The local changes of A/B compartments among the m-bud, g-bud and m-leaf samples showed that chromatin organization is associated with differences of ecotypes or tissues at the A/B compartments architecture level in *Jatropha* (Figure 4).

TAD domains are principal chromatin structural units, in which the frequency of chromatin interactions is higher than that within the boundary regions and reflects distinct and autonomously regulated regions of chromosomes [29, 30, 32, 36]. In

Jatropha, we detected 1376, 1293 and 847 TAD domains at 10 kb resolution from the m-bud, g-bud and m-leaf samples, respectively, with the arrowhead algorithm in the Juicer pipeline [62]. The median length of the TADs was 120-130 kb, and they covered approximately 31.0-58.9% of the *Jatropha* genome in the three samples (Additional Figure S7). Changes in the TAD domains occurred across the three samples, implying that chromatin organization may participate in the processes of phenotypic change or tissue and organ differentiation at the TAD structural level in *Jatropha* (Figure 5A). In rice, the density of protein-coding genes is much lower within TAD interior regions than in the boundary regions between TADs, and the formation of TAD domains may be relevant to histone modifications and gene transcriptions [39, 40]. In *Jatropha,* however, gene distribution changes in the TAD boundary regions were not so marked as in rice, suggesting that chromatin organization may be different between grasses and woody plants (Figure 5B).

The chromatin loop is a fine chromatin structure that brings distant DNA elements and their target genes into close proximity, facilitating transcriptional activation [37]. We detected 2221, 2409 and 371 chromatin loops from the contact matrices of the m-bud, g-bud and m-leaf samples, respectively ((Additional Table S3), using the HiCCUPS algorithm in the Juicer pipeline [62]. These chromatin loops were confirmed using the Aggregate peak analysis (APA) algorithm in the Juicer pipeline (Additional Figure S8) [62]. Different chromatin loops were present in the pairwise comparisons g-bud vs. m-bud and m-leaf vs. m-bud, suggesting that chromatin organization is relevant to differences between ecotypes or tissues at the chromatin loop level (Figure 5A and Additional Table S3).

These chromatin architectures play important roles in the regulation of gene expression in various cellular processes [61]. We monitored obvious alterations of local chromatin organization, such as A/B compartments, TAD domains and chromatin loops across the m-bud, g-bud and m-leaf samples (Figures 4 and 6). These results showed that chromatin organization is intimately associated with different

sexual ecotypes and organ morphologies and may participate in their morphogenetic processes in *Jatropha*.

Differential contacts in monoecious and gynoecious inflorescence buds

To further investigate the function of chromatin architecture in sex differentiation, we detected differential chromatin interactions between gynoecious and monoecious inflorescence buds using the HiCcompare package [63]. A total of 2,425-3,036 differential contacts were identified with a false discovery rate (FDR) of ≤ 0.05 at 5-100 kb resolution (Additional Table S4). In addition, we identified 1,165 differentially expressed genes (DEGs) between gynoecious and monoecious inflorescence buds with an FDR of ≤ 0.05 and a fold change ≥ 2.0 using our published transcriptome data (Additional Table S5) [64]. Then, we calculated the distribution of DEG promoters in the differential contact regions to investigate whether differential contact regions are relevant to the expression of DEGs. Among these genes, the promoter regions of 241 DEGs overlapped with 223 differential contact regions at both 5 kb and 10 kb resolutions, implying that these genes may be regulated by DNA regulatory elements located in the corresponding differential contact regions (Additional Table S6). The promoter density of the DEGs was obviously higher in the differential contact regions than in other regions (background), suggesting that DEG transcription is intimately linked to differential contact, which results from alteration of chromatin organization between gynoecious and monoecious inflorescence buds (Figure 7A). The result supports the hypothesis that chromatin architecture may participate in the process of sex differentiation in *Jatropha*.

Genes involved in sex differentiation and their genomic interaction patterns

We identified twelve genes from the 241 DEGs located in differential contact regions, eight of which are involved in flower development and four in hormone synthesis (Additional Table S7). These genes had different genomic interaction patterns between gynoecious and monoecious inflorescence buds, suggesting that

their expression may be regulated by corresponding regulatory elements during sex differentiation (Figure 7B and Additional Figure S9). For example, *JcSTP8* (*jc002715*) and *JcJMT* (*jc008699*) had different interaction loci at 5 kb resolution between the m-bud and g-bud samples, which may play important roles in promoting the expression of *JcSTP8* and inhibiting the expression of *JcJMT*, respectively (Figure 7B and Additional Table S7). In *Arabidopsis*, SUGAR TRANSPORT PROTEIN 8 (STP8) is a sugar transport protein that contributes to the uptake of glucose in pollen development and pollen tube growth [65, 66]; JASMONIC ACID CARBOXYL METHYLTRANSFERASE (JMT) is a jasmonic acid carboxyl methyltransferase that catalyses the formation of methyljasmonate from JA [67]. *JcSTP8* and *JcJMT*, together with the other genes identified, may participate in sex differentiation in *Jatropha*, and chromatin organization may play an important role in the regulation of their transcription.

Discussion

Chromatin organization is an important factor in regulating gene transcription in many cellular processes, and dynamic alteration of chromatin architecture plays vital roles in responses to environmental stimuli in plants [39, 61, 68-70]. The three-dimensional (3D) structure of each chromosome contains three hierarchical functional substructure units: A/B compartments, TAD domains and chromatin loops [32-35]. In *Jatropha*, the same hierarchical chromatin substructures are found in the nucleus with the Hi-C approach, as in *Arabidopsis* and several crop species [39, 40, 61], implying that these chromatin architectures should be widely present in plants. In mammals, the structural features of TADs are highly conserved across different species, but the same is not true in plants; this difference might be because of the absence of the CTCF protein, which is highly enriched at TAD borders in mammalian systems [32, 71]. The non-conservation of chromatin architecture may contribute to the adaptation of plants to various environmental conditions. The dynamic alteration of chromatin architecture observed across the m-bud, g-bud and m-leaf samples

suggests that chromatin organization is associated with differences between sexual ecotypes or organ morphologies in *Jatropha*.

By examining both DEGs and differential contacts between gynoecious and monoecious inflorescence buds, twelve genes involved in the sex differentiation of *Jatropha* were identified, the expression of which may be regulated by corresponding DNA regulatory elements. In *Arabidopsis*, *INCREASE IN BONSAI METHYLATION 1* (*IBM1*) encodes a histone demethylase suppressing DNA methylation and gene silencing; the *ibm1* mutant displays developmental defects [72, 73]. *RECEPTOR-LIKE KINASE IN FLOWERS 1* (*RKF1*) is highly expressed in early flower primordia and stamen development [74]. *UBIQUITIN LIGASE COMPLEX SUBUNIT 1* (*ULCS1*) encodes a WD40-repeat protein, RNAi-mediated silencing of which causes sterile plants with pleiotropic phenotypes [75]. *TERPENE SYNTHASE 21* (*TPS21*) is a sesquiterpene synthase gene expressed in stigmas, anthers and sepals and is responsible for the formation of floral volatile sesquiterpenes [76]. (*ZUSAMMEN-CA*)-*ENHANCED 1* (*ZCE1*) encodes a member of the major latex protein-like gene family and plays a role in promoting vegetative growth and delaying flowering [77]. In *Jatropha*, corresponding homologous genes, namely, *JcIBM1* (*jc006371*), *JcRKF1* (*jc023149*), *JcULCS1* (*jc023230*), *JcTPS21* (*jc019906*), *JcZCE1* (*jc021698*) and *JcSTP8*, are more highly expressed in gynoecious inflorescence buds than in monoecious ones. *MATRIX METALLOPROTEINASE* (*MMP*) is a member of the matrix metalloproteinase gene family; the *Arabidopsis mmp-1* mutant displays late flowering and early senescence phenotypes [78]. *FERONIA* (*FER*) encodes a plasma membrane receptor protein kinase regulating reproductive growth in *Arabidopsis* [48]. *GIBBERELLIN 2-OXIDASE 8* (*GA2OX8*) encodes a gibberellin (GA) 2-oxidase participating in the GA biosynthetic process in *Arabidopsis* [79]. Four homologous *Jatropha* genes, *JcMMP* (*jc004196*), *JcFER* (*jc003891*), *JcGA2OX8* (*jc021138*) and *JcJMT*, are downregulated in gynoecious inflorescence buds compared to monoecious ones. Moreover, *tRNA ISOPENTENYLTRANSFERASE 2* (*IPT2*) and *ISOPENTENYLTRANSFERASE 5* (*IPT5*) encode cytokinin synthases that catalyse the

first step in cytokinin biosynthesis in *Arabidopsis* [80]. In *Jatropha*, the expression of *JcIPT2* (*jc006165*) is upregulated in gynoecious inflorescence buds compared to monoecious ones, while *JcIPT5* (*jc020647*) is downregulated. In *Arabidopsis*, ATP/ADP IPTs (IPT1, 3–8) are responsible for isopentenyladenine- and *trans*-zeatin (*t*Z)-type cytokinin synthesis and tRNA IPTs (IPT2 and 9) for *cis*-zeatin (*c*Z)-type cytokinin synthesis [80]. These results suggest that different types of cytokinins may play different roles in the sex differentiation of *Jatropha*. All of these genes have different genomic interaction patterns in gynoecious and monoecious samples, implying that their transcription is associated with chromatin organization and the regulation of sex differentiation in *Jatropha*.

Conclusions

In this study, we reported a chromosome-level *de novo* assembly of the *Jatropha* genome using PacBio sequencing combined with Hi-C technology. Based on this high-quality reference genome, we first revealed the features of chromatin architecture in perennial woody plants and investigated the possible function of chromatin organization in sex differentiation in *Jatropha*, which will facilitate understanding of the regulatory mechanisms of sex determination in higher plants.

Methods

Plant materials

Two-year-old gynoecious and monoecious plants were grown in the field in the Xishuangbanna Tropical Botanical Garden of the Chinese Academy of Sciences, Yunnan province, China. Inflorescence buds and leaves from gynoecious and monoecious plants were fixed for Hi-C library construction. Leaves of monoecious plants were frozen for PacBio sequencing. Two biological replicates per sample were generated for Hi-C library construction.

PacBio sequencing and *de novo* assembly

PacBio sequencing was performed on a PacBio Sequel sequencer by Novogene Bioinformatics Technology (Beijing, China). After the polymerase reads were filtered (minReadScore = 0.8), the filtered subreads were used for first-round assembly using the FALCON package (version 0.3.0) with the following parameters: length cutoff = 1000, seed coverage = 35 and length cutoff pre-assembly = 11000 [52]. The contig sequences produced were corrected with PacBio sequencing data using the arrow algorithm in PacBio SMRT Link (v5.1.0). Combined with Hi-C sequencing data, the contig sequences were then integrated into a candidate chromosome-scale assembly using a 3D *de novo* assembly (3D DNA) pipeline [50]. The candidate assembly was further corrected for the final genome sequences using Juicebox Assembly Tools [53].

Genome annotation and quality evaluation

After masking repetitive sequences based on a custom repeat library with the RepeatModeler package (http://www.repeatmasker.org), the assembly of monoecious *Jatropha* was annotated using the MAKER genome annotation pipeline (version 2.31.10) [54, 55]. Both transcript and protein sequences were used for *a b initio* gene prediction. The transcript sequences were *de novo* assembled with our previous transcriptome sequencing data (SRP092157) and NCBI RefSeq *Jatropha* transcript data using Trinity (version 2.2.0) with the default parameters [81, 82]. The protein sequences were from the Ensembl Plants Database (http://plants.ensembl.org). The SNAP and AUGUSTUS programs in MAKER were used to the train the gene prediction model. A detailed description of the MAKER pipeline is provided on the MAKER Wiki page (http://weatherby.genetics.utah.edu/MAKER/wiki/index.php/Main_Page). The AED algorithm was used for assembly annotation [56]. The QUAST-LG, BUSCO (version 3.0), mummer (version 4.0) and MCScanX packages were used to assess assembly completeness and contiguity [57-60]. Single-base substitutions and short insertions and deletions in assembly were estimated with PacBio long-read alignments using the arrow algorithm in Pacific SMRT Link software (v5.1.0)

(https://www.pacb.com/support/software-downloads). A visual Hi-C-based chromatin interaction map approach was used to assess misassemblies, such as structural errors, using the Juicebox Assembly Tools [53].

Hi-C library preparation

The Hi-C protocol was adapted for library construction as previously described [83]. Plant materials were fixed with 2% formaldehyde solution at room temperature for 30 min in a vacuum. Then 2.5 M glycine was added to quench the crosslinking reaction. Approximately 0.5 g of fixed tissue was ground with liquid nitrogen for DNA isolation. The extracted nuclei were resuspended with 0.5% SDS and incubated at 62 °C for 5 min, and 10% Triton X-100 was added and incubated at 37 °C for 15 min. The denatured DNA was digested with the 4-cutter restriction enzyme DpnII at 37 °C overnight. The DpnII enzyme was inactivated at 62 °C for 20 min. Next, the digested DNA was blunt-ended by filling in nucleotides with Klenow enzyme at 37 °C for 2 h. The proximal chromatin DNA was religated with T4 DNA ligation enzyme at room temperature for 4 h. After centrifugation at 1000 ×g for 3 min, the reaction mixture was resuspended with SDS buffer (50 mM Tris-HCl, 1% SDS, 10 mM EDTA, pH 8.0), and proteinase K was added and incubated at 55 °C for 30 min. Formaldehyde crosslinks of nuclear complexes were reversed by adding 30 μl of 5 M NaCl and incubated at 65 °C overnight. Subsequent manipulations were carried out as previously described [83]. The final Hi-C library sequencing was performed on an Illumina HiSeq-2500 platform with PE 150 bp reads.

Hi-C data analysis pipeline

Analysis of the Hi-C sequencing data was performed using the Juicer pipeline [62]. Duplicate and near-duplicate reads mapped to the same restriction fragment were removed and then filtered with mapping quality scores. The contact matrices were normalized at different resolutions. Eigenvectors were identified with the eigenvector algorithm, the sign of which indicates compartment A or compartment B; TADs were identified with the arrowhead algorithm; chromatin loops were identified

with the HiCCUPS algorithm; the aggregate enrichment of putative peaks in contact matrices was validated with the APA algorithm as described [34]. Differential chromatin contacts between the contact matrices were identified using the HiCcompare R-package [63]. All Hi-C maps were generated using the Juicebox package [53].

DEGs between monoecious and gynoecious inflorescence buds

Our previous transcriptome data were reanalysed to examine DEGs between monoecious and gynoecious inflorescence buds. Sequencing reads were mapped to the new *Jatropha* reference genome using the Subread package (version 1.6.2) with the default parameters [84, 85]. DEGs with an FDR of ≤ 0.01 and expression fold change ≥ 2.0 were identified using the edgeR R package [86].

Availability of supporting data and materials

All high-throughput sequencing reads and the assembly presented in the manuscript have been submitted to the China National GeneBank (CNGB) Nucleotide Sequence Archive (CNSA) under accession number CNP0000449 (https://db.cngb.org/cnsa/review/show/CNP0000449_20190514_84e10f2a).

Declarations

List of abbreviations

ACS1: 1-aminocyclopropane-1 carboxylic acid synthase AED: annotation edit distance APA: aggregate peak analysis BR: brassinosteroid CK: Cytokinin DEG: differentially expressed gene ETH: Ethylene FDR: False discovery rate FER: FERONIA GA: Gibberellin GA2OX8: GIBBERELLIN 2-OXIDASE 8 Hi-C: genome-wide chromosome conformation capture ids1: indeterminate spikelet1 IBM1: INCREASE IN BONSAI METHYLATION 1 IPT2: tRNA

ISOPENTENYLTRANSFERASE 2 IPT5: ISOPENTENYLTRANSFERASE 5 JA: jasmonic acid JMT: JASMONIC ACID CARBOXYL METHYLTRANSFERASE MMP: MATRIX METALLOPROTEINASE na1: nana plant1 RKF1: RECEPTOR-LIKE KINASE IN FLOWERS 1 SMRT: single-molecule, real-time STP8: SUGAR TRANSPORT PROTEIN 8 TAD: topologically associated domain TPS21: TERPENE SYNTHASE 21 ts2: tasselseed2 ULCS1: UBIQUITIN LIGASE COMPLEX SUBUNIT 1 ZCE1: (ZUSAMMEN-CA)-ENHANCED 1

Ethics Statement

The *Jatropha curcas* used in this study is not an endangered or protected species, and no specific permissions were required for the study in this paper.

Consent for publication

Not applicable.

Competing interests

The authors declare that they have no competing interests.

Funding

This work was supported by the National Natural Science Foundation of China (31670612, 31870291, 31300568, 31370595 and 31571347), the Programme of the Chinese Academy of Sciences (kfj-brsn-2018-6-008 and 2017XTBG-T02) and Guangdong Science and Technology Department (2016A030313642).

Author Contributions

L-JN, M-SC, CH, and Z-FX designed the study and wrote the paper. L-JN

performed the Hi-C experiments. M-LZ, CX, B-ZP, Q-TF, Y-BT, and H-YH carried out additional experiments. M-SC and L-JN analysed and interpreted the data. All authors reviewed the final manuscript.

Acknowledgements

We gratefully acknowledge the Central Laboratory of the Xishuangbanna Tropical Botanical Garden for providing high-performance computing and other research facilities.

References

1. Aryal R and Ming R. Sex determination in flowering plants: papaya as a model system. Plant Sci. 2014;217-218:56-62. doi:10.1016/j.plantsci.2013.10.018.
2. Bawa KS. Evolution of dioecy in flowering plants. Annu Rev Ecol Syst. 1980;11:15-39. doi:10.1146/annurev.es.11.110180.000311.
3. Thomson JD and Barrett SCH. Selection for outcrossing, sexual selection, and the evolution of dioecy in plants. The American Naturalist. 1981;118:443-9. doi:10.1086/283837.
4. Ainsworth C, Parker J and Buchanan-Wollaston V. Sex determination in plants. Current Topics in Developmental Biology. 1998;38:167-223. doi:10.4161/psb.2.3.3728.
5. Chuck G. Molecular mechanisms of sex determination in monoecious and dioecious plants. Adv Bot Res. 2010;54:53-83. doi:10.1016/S0065-2296(10)54002-3.
6. Diggle PK, Di Stilio VS, Gschwend AR, Golenberg EM, Moore RC, Russell JRW, et al. Multiple developmental processes underlie sex differentiation in angiosperms. Trends Genet. 2011;27 9:368-76. doi:10.1016/j.tig.2011.05.003.
7. Perl-Treves R. Male to female conversion along the cucumber shoot: approaches to studying sex genes and floral development in *Cucumis sativus*. In: Ainsworth CC, editor. Sex determination in plants. Oxford: BIOS Scientific Publishers; 1999. p. 189-286.
8. Mibus H and Tatlioglu T. Molecular characterization and isolation of the *F/f* gene for femaleness in cucumber (*Cucumis sativus* L.). Theor Appl Genet. 2004;109 8:1669-76. doi:10.1007/s00122-004-1793-7.
9. Kamachi Si, Sekimoto H, Kondo N and Sakai S. Cloning of a cDNA for a 1-aminocyclopropane-1-carboxylate synthase that is expressed during development of female flowers at the apices of *Cucumis sativus* L. Plant Cell Physiol. 1997;38 11:1197-206. doi:10.1093/oxfordjournals.pcp.a029106.
10. Li Z, Huang S, Liu S, Pan J, Zhang Z, Tao Q, et al. Molecular isolation of the *M* gene suggests that a conserved-residue conversion induces the formation of bisexual flowers in cucumber plants. Genetics. 2009;182 4:1381-5. doi:10.1534/genetics.109.104737.
11. Poole CF and Grimball PC. Inheritance of new sex forms in *Cucumis melo* L. J Hered. 1939;30 1:21-5.

12. Boualem A, Fergany M, Fernandez R, Troadec C, Martin A, Morin H, et al. A conserved mutation in an ethylene biosynthesis enzyme leads to andromonoecy in melons. Science. 2008;321 5890:836-8. doi:10.1126/science.1159023.
13. Martin A, Troadec C, Boualem A, Rajab M, Fernandez R, Morin H, et al. A transposon-induced epigenetic change leads to sex determination in melon. Nature. 2009;461 7267:1135-8. doi:10.1038/nature08498.
14. Boualem A, Troadec C, Camps C, Lemhemdi A, Morin H, Sari M-A, et al. A cucurbit androecy gene reveals how unisexual flowers develop and dioecy emerges. Science. 2015;350 6261:688-91. doi:10.1126/science.aac8370.
15. Akagi T, Henry IM, Tao R and Comai L. A Y-chromosome-encoded small RNA acts as a sex determinant in persimmons. Science. 2014;346 6209:646-50. doi:10.1126/science.1257225.
16. Acosta IF, Laparra H, Romero SP, Schmelz E, Hamberg M, Mottinger JP, et al. *tasselseed1* is a lipoxygenase affecting jasmonic acid signaling in sex determination of maize. Science. 2009;323 5911:262-5. doi:10.1126/science.1164645.
17. DeLong A, Calderon-Urrea A and Dellaporta SL. Sex determination gene *TASSELSEED2* of maize encodes a short-chain alcohol dehydrogenase required for stage-specific floral organ abortion. Cell. 1993;74 4:757-68. doi:10.1016/0092-8674(93)90522-R.
18. Chuck G, Meeley R, Irish E, Sakai H and Hake S. The maize *tasselseed4* microRNA controls sex determination and meristem cell fate by targeting *Tasselseed6/indeterminate spikelet1*. Nat Genet. 2007;39 12:1517-21. doi:10.1038/ng.2007.20.
19. Hartwig T, Chuck GS, Fujioka S, Klempien A, Weizbauer R, Potluri DP, et al. Brassinosteroid control of sex determination in maize. Proc Natl Acad Sci USA. 2011;108 49:19814-9. doi:10.1073/pnas.1108359108.
20. Louis JP and Durand B. Studies with the dioecious angiosperm *Mercurialis annua* L. (2n=16): Correlation between genic and cytoplasmic male sterility, sex segregation and feminizing hormones (cytokinins). Molecular and General Genetics MGG. 1978;165 3:309-22. doi:10.1007/bf00332532.
21. Chailakhyan MK. Genetic and hormonal regulation of growth, flowering, and sex expression in plants. Am J Bot. 1979;66 6:717-36. doi:10.2307/2442417.
22. Durand R and Durand B. Sexual differentiation in higher plants. Physiol Plant. 1984;60 3:267-74. doi:10.1111/j.1399-3054.1984.tb06061.x.
23. Irish EE and Nelson T. Sex determination in monoecious and dioecious plants. Plant Cell. 1989;1 8:737-44. doi:10.1105/tpc.1.8.737.
24. Korpelainen H. Labile sex expression in plants. Biological Reviews. 1998;73 2:157-80. doi:10.1111/j.1469-185X.1997.tb00028.x.
25. Field DL, Pickup M and Barrett SCH. Comparative analyses of sex-ratio variation in dioecious flowering plants. Evolution. 2013;67 3:661-72. doi:10.1111/evo.12001.
26. Golenberg EM and West NW. Hormonal interactions and gene regulation can link monoecy and environmental plasticity to the evolution of dioecy in plants. Am J Bot. 2013;100 6:1022-37. doi:10.3732/ajb.1200544.
27. Piferrer F. Epigenetics of sex determination and gonadogenesis. Dev Dyn. 2013;242 4:360-70. doi:10.1002/dvdy.23924.
28. Gibcus JH and Dekker J. The hierarchy of the 3D genome. Mol Cell. 2013;49 5:773-82. doi:10.1016/j.molcel.2013.02.011.

29. Sexton T and Cavalli G. The role of chromosome domains in shaping the functional genome. Cell. 2015;160 6:1049-59. doi:https://doi.org/10.1016/j.cell.2015.02.040.
30. Sexton T, Yaffe E, Kenigsberg E, Bantignies F, Leblanc B, Hoichman M, et al. Three-dimensional folding and functional organization principles of the *Drosophila* genome. Cell. 2012;148 3:458-72. doi:https://doi.org/10.1016/j.cell.2012.01.010.
31. Jin F, Li Y, Dixon JR, Selvaraj S, Ye Z, Lee AY, et al. A high-resolution map of the three-dimensional chromatin interactome in human cells. Nature. 2013;503:290-4. doi:10.1038/nature12644
32. Dixon JR, Selvaraj S, Yue F, Kim A, Li Y, Shen Y, et al. Topological domains in mammalian genomes identified by analysis of chromatin interactions. Nature. 2012;485:376-80. doi:10.1038/nature11082
33. Lieberman-Aiden E, van Berkum NL, Williams L, Imakaev M, Ragoczy T, Telling A, et al. Comprehensive mapping of long-range interactions reveals folding principles of the human genome. Science. 2009;326 5950:289-93. doi:10.1126/science.1181369.
34. Rao SSP, Huntley MH, Durand NC, Stamenova EK, Bochkov ID, Robinson JT, et al. A 3D map of the human genome at kilobase resolution reveals principles of chromatin looping. Cell. 2014;159 7:1665-80. doi:https://doi.org/10.1016/j.cell.2014.11.021.
35. Miao Y and Bing R. The three-dimensional organization of mammalian genomes. Annu Rev Cell Dev Biol. 2017;33 1:265-89. doi:10.1146/annurev-cellbio-100616-060531.
36. Nora EP, Lajoie BR, Schulz EG, Giorgetti L, Okamoto I, Servant N, et al. Spatial partitioning of the regulatory landscape of the X-inactivation centre. Nature. 2012;485:381-5. doi:10.1038/nature11049
37. Liu C and Weigel D. Chromatin in 3D: progress and prospects for plants. Genome biology. 2015;16 1:170. doi:10.1186/s13059-015-0738-6.
38. Mousavi K, Zare H, Dell'Orso S, Grontved L, Gutierrez-Cruz G, Derfoul A, et al. eRNAs promote transcription by establishing chromatin accessibility at defined genomic loci. Mol Cell. 2013;51 5:606-17. doi:https://doi.org/10.1016/j.molcel.2013.07.022.
39. Liu C, Cheng YJ, Wang JW and Weigel D. Prominent topologically associated domains differentiate global chromatin packing in rice from *Arabidopsis*. Nature plants. 2017;3 9:742-8. doi:10.1038/s41477-017-0005-9.
40. Dong Q, Li N, Li X, Yuan Z, Xie D, Wang X, et al. Genome-wide Hi-C analysis reveals extensive hierarchical chromatin interactions in rice. plant J. 2018;94 6:1141-56. doi:10.1111/tpj.13925.
41. Fairless D. Biofuel: the little shrub that could--maybe. Nature. 2007;449 7163:652-5. doi:10.1038/449652a.
42. Sato S, Hirakawa H, Isobe S, Fukai E, Watanabe A, Kato M, et al. Sequence analysis of the genome of an oil-bearing tree, *Jatropha curcas* L. DNA Res. 2011;18 1:65-76. doi:10.1093/dnares/dsq030.
43. Ha J, Shim S, Lee T, Kang YJ, Hwang WJ, Jeong H, et al. Genome sequence of *Jatropha curcas* L., a non-edible biodiesel plant, provides a resource to improve seed-related traits. Plant Biotechnol J. 2019;17 2:517-30. doi:doi:10.1111/pbi.12995.
44. Wu P, Zhou C, Cheng S, Wu Z, Lu W, Han J, et al. Integrated genome sequence and linkage map of physic nut (*Jatropha curcas* L.), a biodiesel plant. plant J. 2015;81 5:810-21. doi:doi:10.1111/tpj.12761.
45. Hirakawa H, Tsuchimoto S, Sakai H, Nakayama S, Fujishiro T, Kishida Y, et al. Upgraded genomic information of *Jatropha curcas* L. Plant Biotechnol. 2012;29 2:123-30. doi:10.5511/plantbiotechnology.12.0515a.

46. Kancharla N, Jalali S, Narasimham JV, Nair V, Yepuri V, Thakkar B, et al. *De Novo* sequencing and hybrid assembly of the biofuel crop *Jatropha curcas* L.: identification of quantitative trait loci for geminivirus resistance. Genes. 2019;10 1:69.
47. Rincon-Rabanales M, Vargas-Lopez LI, Adriano-Anaya L, Vazquez-Ovando A, Salvador-Figueroa M and Ovando-Medina I. Reproductive biology of the biofuel plant *Jatropha curcas* in its center of origin. PeerJ. 2016;4:e1819. doi:10.7717/peerj.1819.
48. Haruta M, Gaddameedi V, Burch H, Fernandez D and Sussman MR. Comparison of the effects of a kinase-dead mutation of FERONIA on ovule fertilization and root growth of *Arabidopsis*. FEBS Lett. 2018;592 14:2395-402. doi:doi:10.1002/1873-3468.13157.
49. Burton JN, Adey A, Patwardhan RP, Qiu R, Kitzman JO and Shendure J. Chromosome-scale scaffolding of *de novo* genome assemblies based on chromatin interactions. Nat Biotechnol. 2013;31 12:1119-25. doi:10.1038/nbt.2727.
50. Dudchenko O, Batra SS, Omer AD, Nyquist SK and Hoeger M. *De novo* assembly of the *Aedes aegypti* genome using Hi-C yields chromosome-length scaffolds. Science. 2017;356 6333:92-5. doi:10.1126/science.aal3327.
51. Carvalho CR, Clarindo WR, Praça MM, Araújo FS and Carels N. Genome size, base composition and karyotype of *Jatropha curcas* L., an important biofuel plant. Plant Sci. 2008;174 6:613-7. doi:https://doi.org/10.1016/j.plantsci.2008.03.010.
52. Chin CS, Peluso P and Sedlazeck FJ. Phased diploid genome assembly with single-molecule real-time sequencing. Nat Methods. 2016;13 12:1050-4. doi:10.1038/nmeth.4035.
53. Durand NC, Robinson JT, Shamim MS, Machol I, Mesirov JP, Lander ES, et al. Juicebox provides a visualization system for Hi-C contact maps with unlimited zoom. Cell systems. 2016;3 1:99-101. doi:10.1016/j.cels.2015.07.012.
54. Campbell MS, Holt C, Moore B and Yandell M. Genome annotation and curation using MAKER and MAKER-P. Current protocols in bioinformatics. 2014;48 1:4.11.1-4..39. doi:10.1002/0471250953.bi0411s48.
55. Cantarel BL, Korf I, Robb SM, Parra G, Ross E, Moore B, et al. MAKER: an easy-to-use annotation pipeline designed for emerging model organism genomes. Genome Res. 2008;18 1:188-96. doi:10.1101/gr.6743907.
56. Eilbeck K, Moore B, Holt C and Yandell M. Quantitative measures for the management and comparison of annotated genomes. BMC Bioinformatics. 2009;10 1:67. doi:10.1186/1471-2105-10-67.
57. Mikheenko A, Prjibelski A, Saveliev V, Antipov D and Gurevich A. Versatile genome assembly evaluation with QUAST-LG. Bioinformatics. 2018;34 13:i142-i50. doi:10.1093/bioinformatics/bty266.
58. Simão FA, Waterhouse RM, Ioannidis P, Kriventseva EV and Zdobnov EM. BUSCO: assessing genome assembly and annotation completeness with single-copy orthologs. Bioinformatics. 2015;31 19:3210-2. doi:10.1093/bioinformatics/btv351.
59. Kurtz S, Phillippy A, Delcher AL, Smoot M, Shumway M, Antonescu C, et al. Versatile and open software for comparing large genomes. Genome Biology. 2004;5 2:R12. doi:10.1186/gb-2004-5-2-r12.
60. Wang Y, Tang H, DeBarry JD, Tan X, Li J, Wang X, et al. MCScanX: a toolkit for detection and evolutionary analysis of gene synteny and collinearity. Nucleic Acids Res. 2012;40 7:e49-e. doi:10.1093/nar/gkr1293.

61. Doğan ES and Liu C. Three-dimensional chromatin packing and positioning of plant genomes. Nature plants. 2018;4 8:521-9. doi:10.1038/s41477-018-0199-5.
62. Durand NC, Shamim MS, Machol I, Rao SSP, Huntley MH, Lander ES, et al. Juicer provides a one-click system for analyzing loop-resolution Hi-C experiments. Cell systems. 2016;3 1:95-8. doi:https://doi.org/10.1016/j.cels.2016.07.002.
63. Stansfield JC, Cresswell KG, Vladimirov VI and Dozmorov MG. HiCcompare: an R-package for joint normalization and comparison of HI-C datasets. BMC Bioinformatics. 2018;19 1:279. doi:10.1186/s12859-018-2288-x.
64. Chen M-S, Pan B-Z, Fu Q, Tao Y-B, Martínez-Herrera J, Niu L, et al. Comparative transcriptome analysis between gynoecious and monoecious plants identifies regulatory networks controlling sex determination in *Jatropha curcas*. front plant sci. 2016;7:1953. doi:10.3389/fpls.2016.01953.
65. Rottmann T, Klebl F, Schneider S, Kischka D, Rüscher D, Sauer N, et al. Sugar transporter STP7 specificity for L-arabinose and D-xylose contrasts with the typical hexose transporters STP8 and STP12. Plant Physiol. 2018;176 3:2330-50. doi:10.1104/pp.17.01493.
66. Rottmann T, Fritz C, Sauer N and Stadler R. Glucose uptake via STP transporters inhibits in vitro pollen tube growth in a HEXOKINASE1-dependent manner in *Arabidopsis thaliana*. The Plant Cell. 2018;30 9:2057-81. doi:10.1105/tpc.18.00356.
67. Seo HS, Song JT, Cheong J-J, Lee Y-H, Lee Y-W, Hwang I, et al. Jasmonic acid carboxyl methyltransferase: a key enzyme for jasmonate-regulated plant responses. Proc Natl Acad Sci USA. 2001;98 8:4788-93.
68. Probst AV and Mittelsten Scheid O. Stress-induced structural changes in plant chromatin. Curr Opin Plant Biol. 2015;27:8-16. doi:https://doi.org/10.1016/j.pbi.2015.05.011.
69. Li L, Lyu X, Hou C, Takenaka N, Nguyen Huy Q, Ong C-T, et al. Widespread rearrangement of 3D chromatin organization underlies polycomb-mediated stress-induced silencing. Mol Cell. 2015;58 2:216-31. doi:https://doi.org/10.1016/j.molcel.2015.02.023.
70. Rosa S and Shaw P. Insights into chromatin structure and dynamics in plants. Biology. 2013;2 4:1378.
71. Dong P, Tu X, Chu P-Y, Lü P, Zhu N, Grierson D, et al. 3D chromatin architecture of large plant genomes determined by local A/B compartments. Molecular plant. 2017;10 12:1497-509. doi:https://doi.org/10.1016/j.molp.2017.11.005.
72. Wang Y, Xue X, Zhu J-K and Dong J. Demethylation of ERECTA receptor genes by IBM1 histone demethylase affects stomatal development. Development. 2016;143 23:4452-61. doi:10.1242/dev.129932.
73. Inagaki S, Takahashi M, Hosaka A, Ito T, Toyoda A, Fujiyama A, et al. Gene ‐ body chromatin modification dynamics mediate epigenome differentiation in *Arabidopsis*. The EMBO Journal. 2017;36 8:970-80. doi:10.15252/embj.201694983.
74. Takahashi T, Mu J-H, Gasch A and Chua N-H. Identification by PCR of receptor-like protein kinases from *Arabidopsis* flowers. Plant Mol Biol. 1998;37 4:587-96. doi:10.1023/a:1005924817190.
75. Beris D, Kapolas G, Livanos P, Roussis A, Milioni D and Haralampidis K. RNAi-mediated silencing of the *Arabidopsis thaliana ULCS1* gene, encoding a WDR protein, results in cell wall modification impairment and plant infertility. Plant Sci. 2016;245:71-83. doi:https://doi.org/10.1016/j.plantsci.2016.01.008.
76. Tholl D, Chen F, Petri J, Gershenzon J and Pichersky E. Two sesquiterpene synthases are responsible for the complex mixture of sesquiterpenes emitted from Arabidopsis flowers. plant J.

2005;42 5:757-71. doi:10.1111/j.1365-313X.2005.02417.x.

77. Guo D, Wong WS, Xu WZ, Sun FF, Qing DJ and Li N. *Cis-cinnamic acid-enhanced 1* gene plays a role in regulation of *Arabidopsis* bolting. Plant Mol Biol. 2011;75 4:481-95. doi:10.1007/s11103-011-9746-4.
78. Golldack D, Popova OV and Dietz K-J. Mutation of the matrix metalloproteinase at2-MMP inhibits growth and causes late flowering and early senescence in *Arabidopsis*. J Biol Chem. 2002;277 7:5541-7. doi:10.1074/jbc.M106197200.
79. Schomburg FM, Bizzell CM, Lee DJ, Zeevaart JAD and Amasino RM. Overexpression of a novel class of gibberellin 2-oxidases decreases gibberellin levels and creates dwarf plants. The Plant Cell. 2003;15 1:151-63. doi:10.1105/tpc.005975.
80. Miyawaki K, Tarkowski P, Matsumoto-Kitano M, Kato T, Sato S, Tarkowska D, et al. Roles of *Arabidopsis* ATP/ADP isopentenyltransferases and tRNA isopentenyltransferases in cytokinin biosynthesis. Proc Natl Acad Sci USA. 2006;103 44:16598-603.
81. Grabherr MG, Haas BJ, Yassour M, Levin JZ, Thompson DA, Amit I, et al. Full-length transcriptome assembly from RNA-Seq data without a reference genome. Nat Biotechnol. 2011;29 7:644-52. doi:10.1038/nbt.1883.
82. Haas BJ, Papanicolaou A, Yassour M, Grabherr M, Blood PD, Bowden J, et al. *De novo* transcript sequence reconstruction from RNA-seq using the Trinity platform for reference generation and analysis. Nature Protocols. 2013;8 8:1494-512. doi:10.1038/nprot.2013.084.
83. Wang C, Liu C, Roqueiro D, Grimm D, Schwab R, Becker C, et al. Genome-wide analysis of local chromatin packing in *Arabidopsis thaliana*. Genome Res. 2015;25 2:246-56. doi:10.1101/gr.170332.113.
84. Liao Y, Smyth GK and Shi W. The Subread aligner: fast, accurate and scalable read mapping by seed-and-vote. Nucleic Acids Res. 2013;41 10:e108. doi:10.1093/nar/gkt214.
85. Liao Y, Smyth GK and Shi W. featureCounts: an efficient general purpose program for assigning sequence reads to genomic features. Bioinformatics. 2014;30 7:923-30. doi:10.1093/bioinformatics/btt656.
86. Robinson MD, McCarthy DJ and Smyth GK. edgeR: a Bioconductor package for differential expression analysis of digital gene expression data. Bioinformatics. 2010;26 1:139-40. doi:10.1093/bioinformatics/btp616.

Figure legends

Figure 1 BUSCO annotations of the new *Jatropha* genome assembly and other published assemblies. n represents gene number of single-copy orthologs.

Figure 2 Comparison of Hi-C contact maps among the new *Jatropha* genome assembly and the other two assemblies. Red square represents the strongest signal value.

Figure 3 Hi-C contact maps of the m-bud, g-bud and m-leaf samples. (A) Genome-wide Hi-C contact maps and (B) Hi-C contact maps of chromosome 1 at 25 kb resolution. The m-bud label indicates monoecious inflorescence bud samples; g-bud indicates gynoecious inflorescence bud samples; m-leaf indicates monoecious inflorescence leaf samples. Red square represents the strongest signal value.

Figure 4 Comparison of the A/B compartments across the m-bud, g-bud and m-leaf samples. The black box indicates the change of A/B compartments among the three samples; m-bud, g-bud and m-leaf indicate the same samples shown in Figure 3.

Figure 5 Comparisons of TADs and chromatin loops in chromosome 2 across the m-bud, g-bud and m-leaf samples. (A) TADs and chromatin loops in chromosome 2. The yellow box indicates TAD structure; the cyan square indicates chromatin loop structure; the blue arrow indicates TAD regions, and the green arrow indicates chromatin loops; m-bud, g-bud and m-leaf indicate the same samples shown in Figure 3. Red square represents the strongest signal value. (B) Distribution of protein-coding genes around TAD boundaries. The red line represents gene distribution in random TAD regions (background); the shaded green area indicates the inside of the TAD region; m-bud, g-bud and m-leaf indicate the same samples shown in Figure 3.

Figure 6 Comparisons of chromatin architecture in chromosome 2 between the sample pairs g-bud vs. m-bud and m-leaf vs. m-bud. Blue arrows indicate differential TADs;

green arrows indicate differential chromatin loops; m-bud, g-bud and m-leaf indicate the same samples shown in Figure 3. Red square represents the strongest signal value.

Figure 7 Distribution of DEGs in differential contact regions and genomic interaction profiles of the *JcJMT* and *JcSTP8* genes. (A) Distribution of DEGs in differential contact regions in the pairwise comparison g-bud vs. m-bud. (B) Genomic interaction profiles of the *JcJMT* and *JcSTP8* genes in the m-bud and g-bud samples. The m-bud and g-bud labels indicate the same samples shown in Figure 3.

Supplementary Information

Additional Figure S1 Distribution of PacBio subread lengths.

Additional Figure S2 AED score of the annotation of the new *Jatropha* genome assembly.

Additional Figure S3 Length distribution of the sequences of the new *Jatropha* genome assembly and the other assemblies

Additional Figure S4 Comparisons of genomic sequences between the new *Jatropha* genome assembly and the other assemblies.

Additional Figure S5 Comparison of chromosome structures between the new *Jatropha* genome assembly and the published assembly. jc1-11 indicates the chromosome codes of the new *Jatropha* assembly and chr1-11 indicates the chromosome codes of the published *Jatropha* assembly (Ha et al. 2019).

Additional Figure S6 Comparison of chromosome sequences between the new *Jatropha* genome assembly and the published *Jatropha* assembly. jc1-11 indicates the chromosome codes of the new *Jatropha* assembly and chr1-11 indicates the chromosome codes of the published *Jatropha* assembly (Ha et al. 2019).

Additional Figure S7 Distribution of TAD sizes at 10 kb resolution in the m-bud, g-bud and m-leaf samples. The m-bud, g-bud m-leaf labels indicate the same samples shown in Figure 3.

Additional Figure S8 Aggregate peak analysis (APA) of m-bud, g-bud and m-leaf samples. The m-bud, g-bud m-leaf labels indicate the same samples shown in Figure 3.

Additional Figure S9 Genomic interaction profiles of the candidate genes in the

m-bud and g-bud samples. The m-bud and g-bud labels indicate the same samples shown in Figure 3.

Additional Table S1 Statistics of the new *Jatropha* assembly and the other assemblies.

Additional Table S2 Statistics of the Hi-C data of the m-bud, g-bud and m-leaf samples.

Additional Table S3 Chromatin loops identified from the m-bud, g-bud and m-leaf contact matrices.

Additional Table S4 Differential chromatin contacts at 5 kb resolution in the pairwise comparisons g-bud vs. m-bud and m-leaf vs. m-bud.

Additional Table S5 Differentially expressed genes (DEGs) identified from the pairwise comparison g-bud vs. m-bud.

Additional Table S6 DEGs located in differential contact regions at 5 kb and 10 kb resolutions in the pairwise comparison g-bud vs. m-bud.

Additional Table S7 Twelve DEGs involved in sex differentiation located in differential contact regions in the pairwise comparison g-bud vs. m-bud.

 Click here to download Table Table1.docx

Table 1 Statistics of the new *Jatropha* assembly

Assembly	Number	N50	N75	L50	L75	Total length (kb)
Contigs	1,265	1,029,648	362,618	86	246	378,337,367
Scaffolds	1,196	30,651,357	27,306,515	6	10	379,507,867
chromosomes	11	-	-	-	-	337,277,379
Coding genes	25,817	-	-	-	-	40,884,597

Figure 1 Click here to download Figure Figure 1.tif

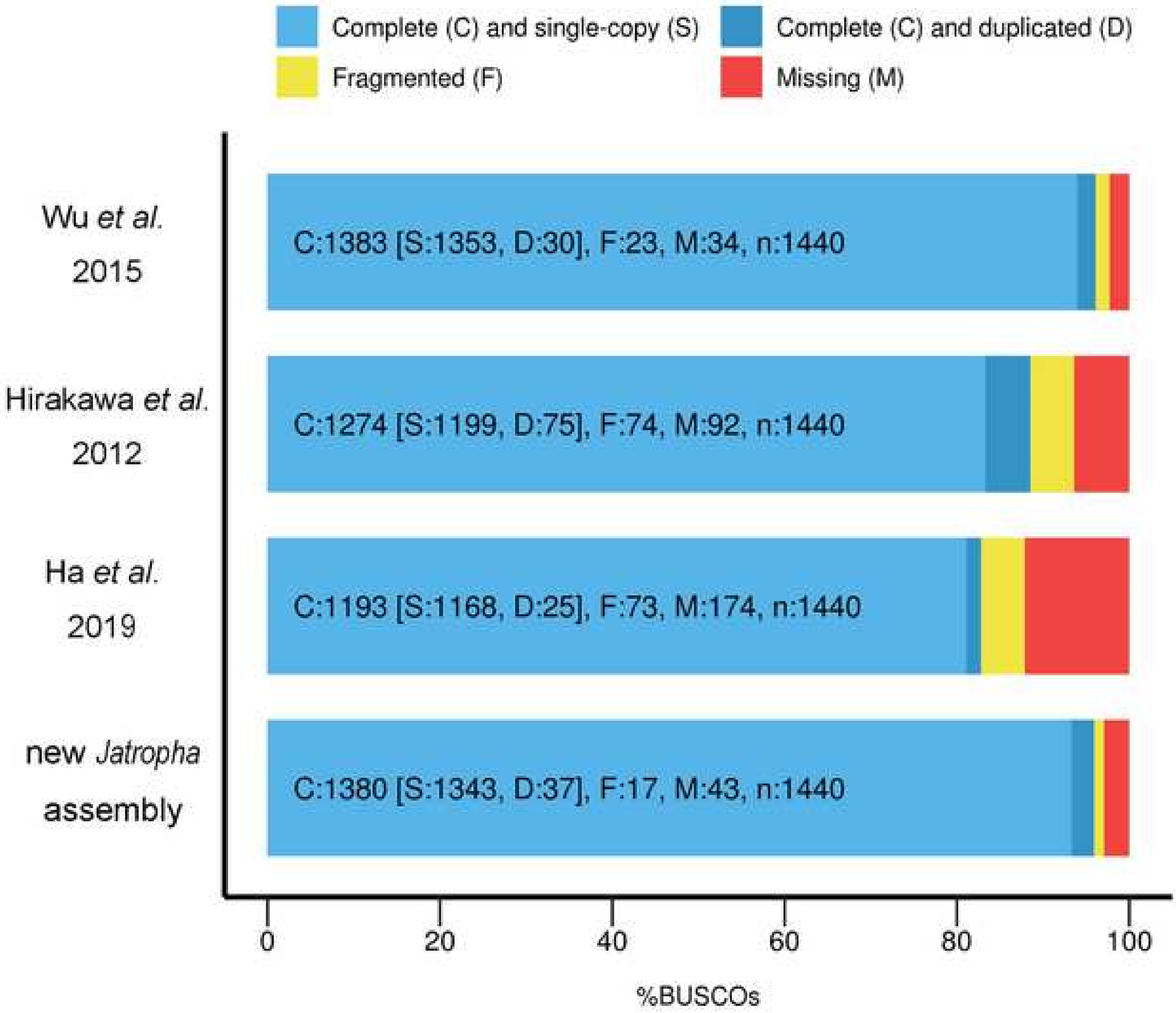

Figure 2

Click here to download Figure Figure 2.tif

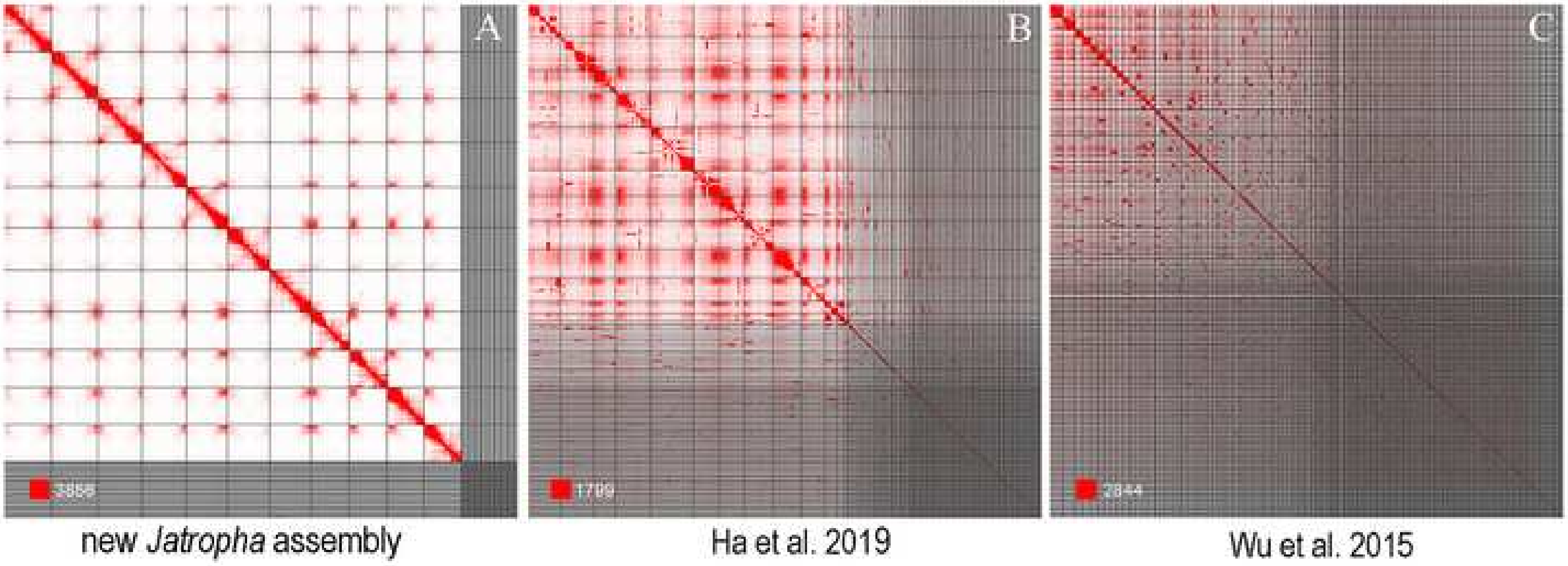

Figure 3

Click here to download Figure Figure 3.tif

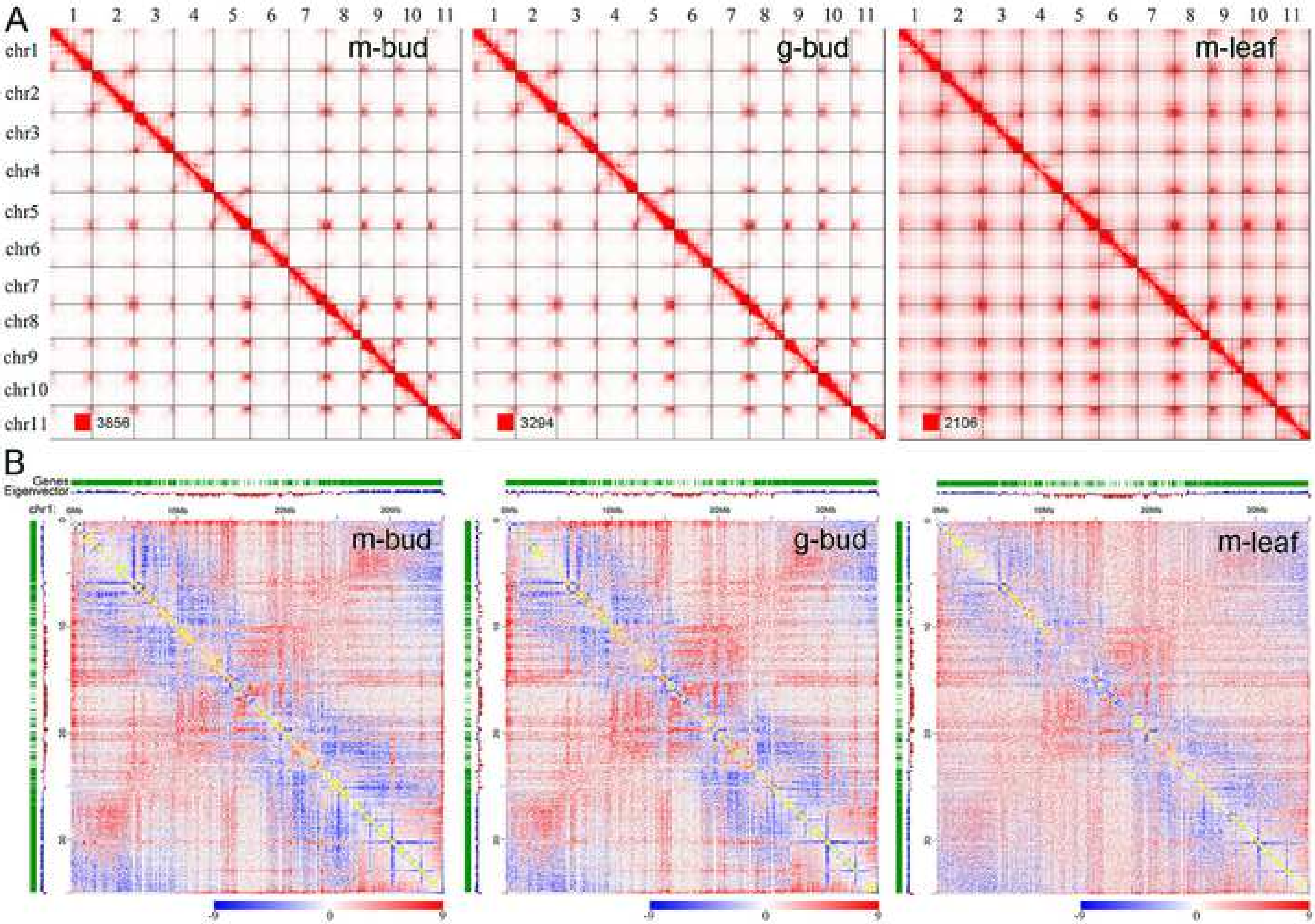

Figure 4

Click here to download Figure Figure 4.tif

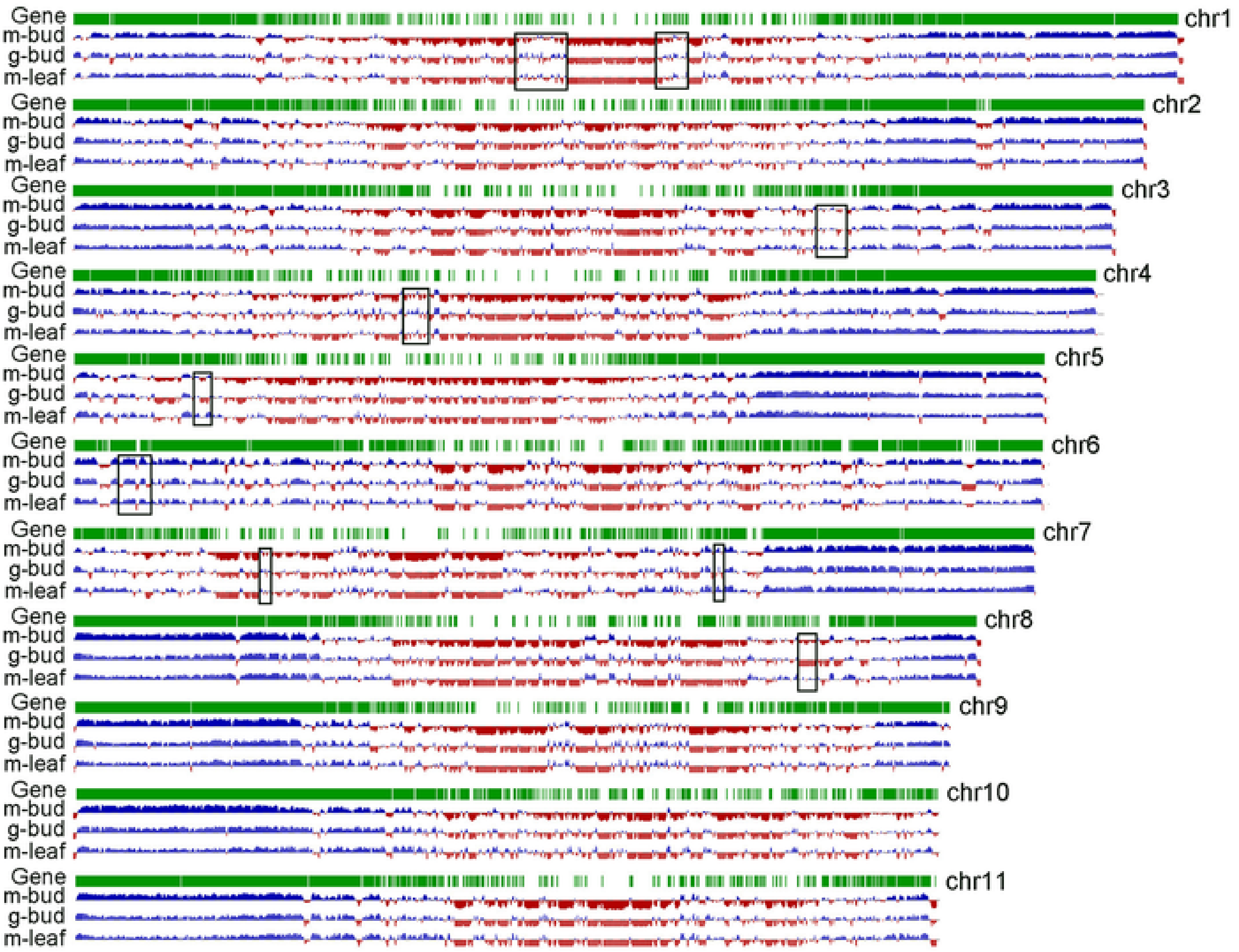

Figure 5

Click here to download Figure Figure 5.tif

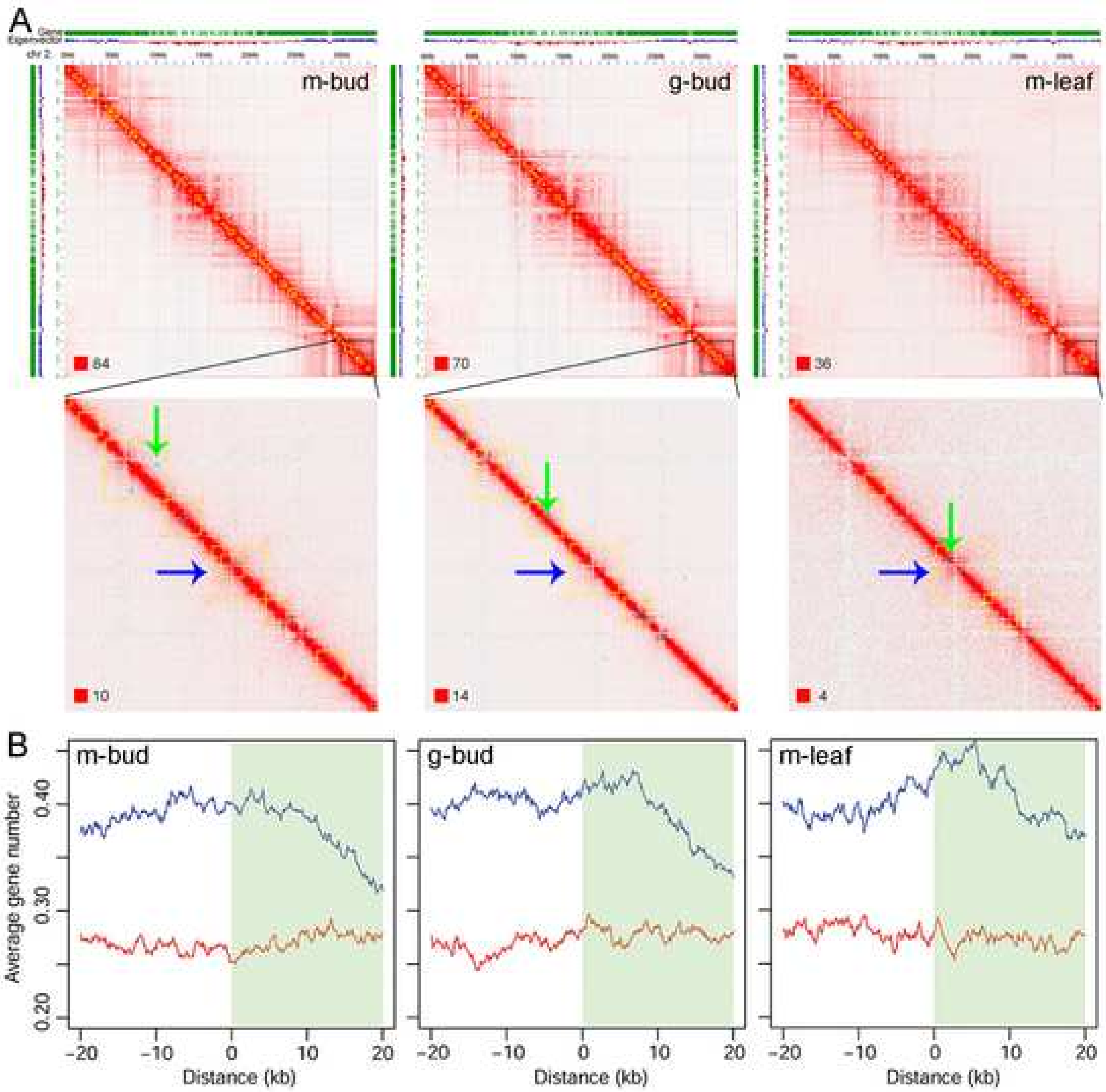

Figure 6

Click here to download Figure Figure 6.tif

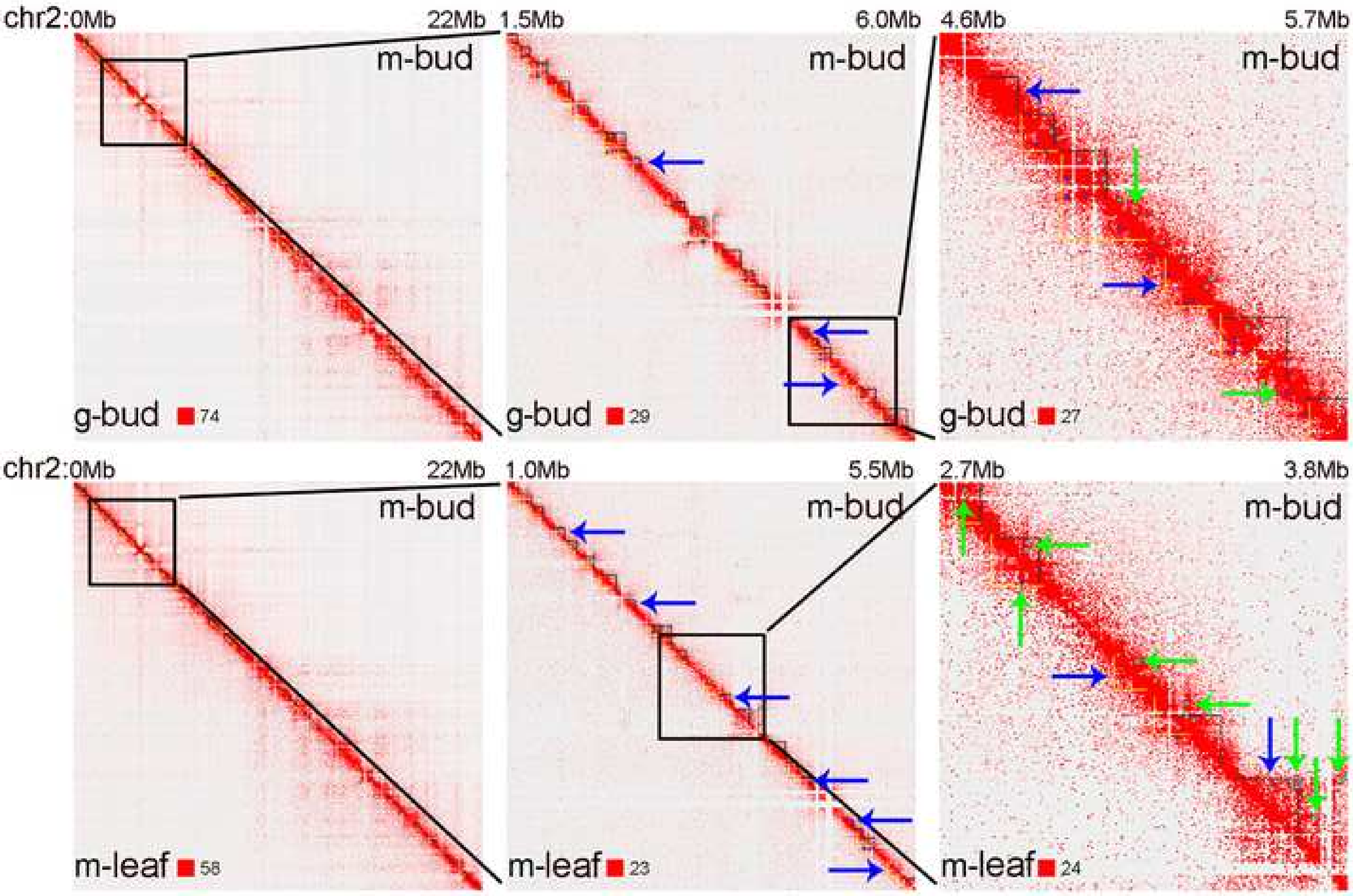

Figure 7

Click here to download Figure Figure 7.tif

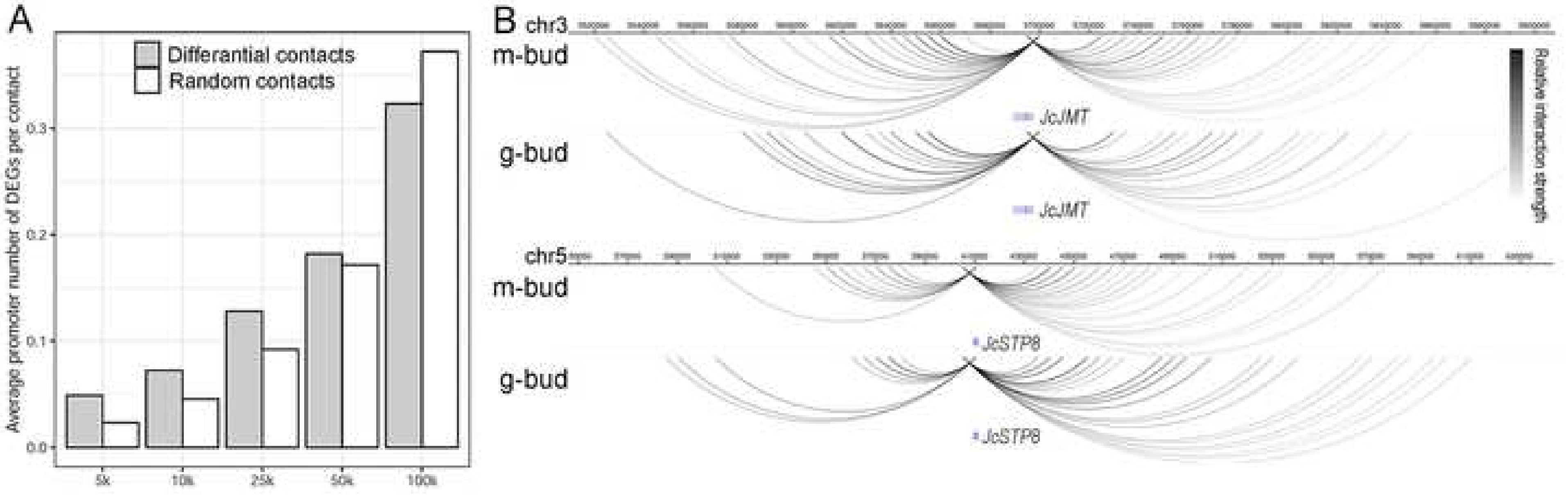

Additional Figure S1

Click here to access/download
Supplementary Material
Additional Figure S1.tif

Additional Figure S2

Click here to access/download
Supplementary Material
Additional Figure S2.tif

Additional Figure S3

Click here to access/download
Supplementary Material
Additional Figure S3.tif

Additional Figure S4

Click here to access/download
Supplementary Material
Additional Figure S4.tif

Additional Figure S5

Click here to access/download
Supplementary Material
Additional Figure S5.tif

Additional Figure S6

Click here to access/download
Supplementary Material
Additional Figure S6.tif

Additional Figure S7

Click here to access/download
Supplementary Material
Additional Figure S7.tif

Additional Figure S8

Click here to access/download
Supplementary Material
Additional Figure S8.tif

Additional Figure S9

Click here to access/download
Supplementary Material
Additional Figure S9.tif

Additional Table S1

Click here to access/download
Supplementary Material
Additional Table S1.docx

Additional Table S2

Click here to access/download
Supplementary Material
Additional Table S2.xlsx

Additional Table S3

Click here to access/download
Supplementary Material
Additional Table S3.xls

Additional Table S4

Click here to access/download
Supplementary Material
Additional Table S4.xlsx

Additional Table S5

Click here to access/download
Supplementary Material
Additional Table S5.xls

Additional Table S6

Click here to access/download
Supplementary Material
Additional Table S6.xlsx

Additional Table S7

Click here to access/download
Supplementary Material
Additional Table S7.xlsx
