# Supplementary material for: De novo genome assembly and Hi-C analysis reveal an association between chromatin architecture alterations and sex differentiation in the woody plant Jatropha curcas
Source: Gigascience. 2020 Feb 12;9(2):giaa009. doi: 10.1093/gigascience/giaa009 (PMC7014976; doi:10.1093/gigascience/giaa009)
Supplement: giaa009_GIGA-D-19-00223_Revision_1 [file giaa009_giga-d-19-00223_revision_1.pdf]

GigaScience

De novo genome assembly and Hi-C analysis reveal the association between chromatin architecture alterations and sex differentiation in the woody plant Jatropha curcas

--Manuscript Draft--

**Manuscript Number:**	GIGA-D-19-00223R1
**Full Title:**	De novo genome assembly and Hi-C analysis reveal the association between chromatin architecture alterations and sex differentiation in the woody plant Jatropha curcas
**Article Type:**	Research
**Funding Information:**	National Natural Science Foundation of China (31670612) — Dr. Mao-Sheng Chen<br>National Natural Science Foundation of China (31971628) — Dr. Mao-Sheng Chen<br>National Natural Science Foundation of China (31870291) — Dr. Bang-Zhen Pan<br>National Natural Science Foundation of China (31300568) — Dr. Qian-Tang Fu<br>National Natural Science Foundation of China (31370595) — Dr. Zeng-Fu Xu<br>National Natural Science Foundation of China (31571347) — Dr. Chunhui Hou<br>Programme of the Chinese Academy of Sciences (kfj-brsn-2018-6-008) — Dr. Zeng-Fu Xu<br>Programme of the Chinese Academy of Sciences (2017XTBG-T02) — Dr. Zeng-Fu Xu<br>Guangdong Science and Technology Department (2016A030313642) — Dr. Chunhui Hou
**Abstract:**	Background: Chromatin architecture is an essential factor in regulating gene transcription in different cell types and developmental phases. However, studies of chromatin architecture in perennial woody plants and the function of chromatin organization in sex determination have not been reported.<br>Results: Here, we report a chromosome-scale de novo genome assembly of the woody plant Jatropha curcas with a total length of 379.5 Mb and scaffold N50 of 30.7 Mb using PacBio long-reads combined with genome-wide chromosome conformation capture (Hi-C) technology. Based on this high-quality reference genome, we detected chromatin architecture differences between the monoecious and gynoecious inflorescence buds of Jatropha . Differentially expressed genes (DEGs) were significantly enriched in the changed A/B compartments and topologically associated domain (TAD) regions and occurred preferentially in differential contact regions between monoecious and gynoecious inflorescence buds. Twelve DEGs related to flower development or hormone synthesis displayed significantly different genomic interaction patterns in monoecious and gynoecious inflorescence buds. These results demonstrate that chromatin organization participates in the regulation of gene transcription during the process of sex differentiation in Jatropha .<br>Conclusions: We revealed the features of chromatin architecture in perennial woody plants and investigated the possible function of chromatin organization in sex differentiation of Jatropha . These findings will facilitate understanding of the regulatory mechanisms of sex determination in higher plants.
**Corresponding Author:**	Zeng-Fu Xu, Ph.D.<br>Xishuangbanna Tropical Botanical Garden

Powered by Editorial Manager® and ProduXion Manager® from Aries Systems Corporation

	Menglun, Mengla, Yunnan CHINA
**Corresponding Author Secondary Information:**	
**Corresponding Author's Institution:**	Xishuangbanna Tropical Botanical Garden
**Corresponding Author's Secondary Institution:**	
**First Author:**	Mao-Sheng Chen
**First Author Secondary Information:**	
**Order of Authors:**	Mao-Sheng Chen
	Longjian Niu
	Mei-Li Zhao
	Chuanjia Xu
	Bang-Zhen Pan
	Qian-Tang Fu
	Yan-Bin Tao
	Hui-Ying He
	Chunhui Hou
	Zeng-Fu Xu, Ph.D.
**Order of Authors Secondary Information:**	
**Response to Reviewers:**	Our point-by-point responses to the comments of the editor and the reviewers are as follows:<br><br>Responses to the comments of the editor:<br>Please also meet our reproducibility guidelines, as we would ask you add identifiers (ORCID IDs for authors, RRIDs for software, NCBI taxon IDs for species, etc.) and would also recommend including protocols in protocols.io.<br>Response: Thanks for your suggestion. The identifiers were added in lines 21-30, 357, 367, 377-392, 418 and 432-441 in the revised manuscript.<br><br>Responses to the comments of the Reviewer 1:<br><br>1. One issue is that the FDR used is unclear. It is 0.05 in page 9 but 0.01 in page 15. From Additional Table S5 it seems like the threshold used was 0.05.<br>Response: Thanks for your comment. An FDR of ≤ 0.05 was used in this study. The error was corrected in line 434 on page 16 in the revised manuscript.<br><br>2. In addition, it would be useful to provide the RNAseq result of all genes so that interested parties can apply their own logFC and FDR thresholds when comparing studies.<br>Response: The RNA-seq result of all genes was included in the Additional Table S7 in the revised manuscript.<br><br>3. Finally a volcano plot that shows the effect of the threshold would also help visualizing the effect of choosing different thresholds.<br>Response: A volcano plot of transcriptome analysis was added as an Additional Figure S8.<br><br>4. Page 9: "These genes" - It is unclear if this refers to 12 or 8 genes. What are the identification criteria for the 12 and 8 genes? Do the remaining 241 genes not follow these criteria?<br>Response: “These genes” indicates the 12 genes, which may be involved in flower development or biosynthesis of phytohormones associated with sex differentiation in Jatropha. We have clarified this in lines 263-276 of the revised manuscript.

5. The version of the software used is missing from all text except the Methods section. Is this acceptable?
Response: We added the version information of the software in all text in the revised manuscript.

6. Fig S6: Please make the new assembly to be collinear with Ha et al, which will make any comparisons easier. Ideally the raw files are as collinear as possible, but at least the figure should be so that all remaining inconsistencies are more likely to be true inversions.
Response: Genome-wide collinearity comparison has displayed in Figure 1B, which is generated with the entire genome sequences in the revised manuscript. The old Additional Figures S5 and S6 were merged into a new Figure 1E to show the collinear comparison of gene coding sequences of corresponding chromosomes between our assembly and the Ha et al. (2019) assembly, in which the differences between chromosomes are originated from respective genome assemblies.

Minor comments
7. Page 2, second last sentence "Among these DEGs" -> insert number of DEGs.
Response: We revised the relevant description in lines 45-49 on page 2 in the revised manuscript.

8. Page 5: "real-time (SMRT)" -> "PacBio", as in first paragraph of results
Response: We revised the "real-time (SMRT)" as "PacBio" in line 135 on page 5 in the revised manuscript.

9. Page 6: "The assembly includes" -> "The assembly is comprised of", or specify what else it includes.
Response: We revised the "The assembly includes" as "The assembly is comprised of" in line 149 on page 6 in the revised manuscript.

10. Page 6: delete ", in which most of the errors were from substitutions"
Response: We revised the relevant description in lines 166-168 on pages 6 and 7 in the revised manuscript.

11. Page 6: The BUSCO result text is unnecessarily long. It is sufficient to say it is best that other assemblies, and use the [] format used in Fig 6 to describe the result.
Response: We revised the description of BUSCO result in lines 172-174 on page 7 in the revised manuscript.

12. Page 8, first paragraph, second last sentence: "transcriptions" -> transcription
Response: We revised "transcriptions" as "transcription" in line 212 on page 8 in the revised manuscript.

13. Page 8: "((Additional Table S3)" -> "(Additional Table S3)"
Response: We removed the extra left bracket in line 221 on page 8 in the revised manuscript.

14. Page 10: remove the non-capitalised "is a sugar transport protein that" and "is a jasmonic acid carboxyle methyltransferase that" as the information just repeats the gene name.
Response: We revised the sentence in lines 282-284 on page 11 in the revised manuscript.

15. Page 13: "used to the train" -> "used to train"
Response: we revised "used to the train" as "used to train" in line 386 on page 14 in the revised manuscript.

16. Fig S3: "Scoffold" -> "Scaffold"
Response: We revised "Scoffold" as "Scaffold" in the new Figure 1D (old Additional Figure S3) in the revised manuscript.

Responses to the comments of the Reviewer 2:

1. Title is too long, May I suggest a title as" Chromatin architecture alterations are

associated with sex differentiation in the woody plant Jatropha curcas".
Response: Thanks for your suggestion. We revised the title as "De novo genome assembly and Hi-C analysis reveal the association between chromatin architecture alterations and sex differentiation in the woody plant Jatropha curcas".

2. Introduction: Authors should include some more literatures on effect of exogenous hormones to Jatropha flowerin. Several works have been reported on a possible หำป sex determination genes.
Response: More literatures were included in lines 94-101 on page 4 in the introduction section of the revised manuscript.

3. Discussions: Page 12: There are a couple of literatures of effect on cytokinin to Jatropha flowering genes expression. Authors need to include these literatures if they want to speculate the important of cytokinin biosynthesis genes.
Response: More literatures were included in lines 343-344 on page 13 in the discussion section of the revised manuscript.

Responses to the comments of the Reviewer 3:

1. The current DEGs part is very general. Even though you have a previous publication on it, new DEGs result should be presented in detail other than simply some values of DEGs. In my view, the DEGs should be summarized on their functional categories (such as GO, Kegg, flower pathways, sexual determination pathways). Then, the readers could have chance to build a comprehensive picture of gene expression related to the biological process, and to the effects of chromatin organization on gene expression. If you include more gene expression (see my next comment), please provide in-depth description on gene expression and co-expression. That is not difficult.
Response: Thanks for your suggestion. We added GO and KEGG enrichment analysis of DEGs (Additional Figure S9, S10, and Additional Table S8) and co-expression genes (Additional Figure S12 and Additional Table S13) in lines 249-252 on pages 9 and 10, and lines 287-293 on page 11, respectively, in the result section of the revised manuscript.

2. Chromatin architecture alternation of sex differentiation is the core point of this article. Comparison between chromatin contact and gene expression units makes the most key part of the analyses present in the study. While, there are still lacking some interesting staff, such as comparison between gene co-expression profile and chromatin organization. The published gene expression reports (including the No. 64 cited in the reference list, and some others listed in the following lines) provide enough data for a gene co-expression study. Please collect more RNA-seq data, enrich the gene expression part, construct gene co-expression profile, compare the gene co-expression and chromatin contact, make in-depth investigation and description, and raise potential hypothesis. We would like to know if such genes contacted in chromatin level go together in gene expression for example in "m-bud", "gbud" and "m-leaf". Or which set of genes do that, which do not? This means the author make need to provide a gene co-expression study, and then for this kind of comparsion. Such kind of analyse is a rountine way to examine the connection between gene expression and chromatin organization. Please see the articles listed below.
Response: We performed co-expression analysis and found that the co-expressed genes related to the “Reproductive process” are irrelevant to chromatin structure changes, however, DEGs preferentially occur in the changed regions of compartment A/B and TADs (Figure 5), implying that chromatin organization is associated with gene transcription. The relevant description is added in lines 293-299 on page 11 in the result section of the revised manuscript.

3. Page 5, "suggesting that chromatin organization may be different between grasses and woody plants". If you only have cases from one grass (here rice) and one woody plant (Jatropha), keep conservative and give up this tandency, as few people will agree with you.
Response: We revised the description in lines 210-217 on page 8 in the revised manuscript.

4. I see some results not well described in the results part but in the discussion part,

Powered by Editorial Manager® and ProduXion Manager® from Aries Systems Corporation

	like all you discussed in Page 11, especially the genes IDs, I did read them in results. Please enrich the results, do not be so general. You may put them into supplementary file, if you are worrying about the space.<br>Response: An additional Table S10 was included and the relevant description was added in lines 263-275 on page 10 in the revised manuscript.<br><br>5. Line number is lacking. And it is very hard for reviewers to provide their comments.<br>Response: Sorry for this mistake. Line number was added in the revised manuscript.<br><br>6. Please replace "new Jatropha assembly" in different Figures or Additional Figures with "our assembly" or "the present assembly".<br>Response: All "new Jatropha assembly" was replaced with "our Jatropha assembly" in all figures in the revised manuscript.<br><br>7. Additional Figures S3. I may want to see different assemblies were contrasted in one panel by overlying different items, but not in separated ones. And also transformation could applied to X or Y variables, so as to get good visitation.<br>Response: The old additional Figure S3 was replaced with a new Figure 1D in the revised manuscript.<br><br>8. Why the assembly from Kancharla et al. 2019 was never compared in analyses presented in Figure 2, Additional Figure S3 and other Figures? But in that in Additional Table S1?<br>Response: The quality of the assembly from Kancharla et al. 2019 is poor as shown in Additional Table S1. In addition, the assembly from Kancharla et al. 2019 hasn't been used widely in Jatropha research as that of assemblies from Hirakawa et al. 2012 and Wu et al. 2015. Therefore, we didn't perform further comparative analysis.<br><br>9. Additional Figures S5 is a partial one. Please provide an intact one.<br>Response: The old Additional Figures S5 was replaced with a new Figure 1E, showing chromosomal differences between our Jatropha assembly and the assembly from Ha et al. 2019 in the revised manuscript.<br><br>10. Additional Figures S7. Please provide the contrast in one panel, not two or more.<br>Response: The old Additional Figure S7 was replaced with a new Figure 3C in the revised manuscript.<br><br>11. Figure 5, hard to see yellow box and cyan square, only black square could I see. Please refine this figure.<br>Response: The old Figure 5 was removed, and the contents were incorporated into the new Figures 2 and 3 in the revised manuscript.<br><br>12. Figure 6, hard to see red square, only dark squares I see. Please refine this figure.<br>Response: The old Figure 6 was removed, and the contents were incorporated into the new Figure 2 in the revised manuscript.<br><br>We thank the reviewers for the kind help.
**Additional Information:**	
**Question**	**Response**
Are you submitting this manuscript to a special series or article collection?	No
**Experimental design and statistics**<br><br>Full details of the experimental design and statistical methods used should be given in the Methods section, as detailed in our Minimum Standards Reporting Checklist. Information essential to interpreting the	Yes

data presented should be made available in the figure legends.<br><br>Have you included all the information requested in your manuscript?	
**Resources**<br><br>A description of all resources used, including antibodies, cell lines, animals and software tools, with enough information to allow them to be uniquely identified, should be included in the Methods section. Authors are strongly encouraged to cite Research Resource Identifiers (RRIDs) for antibodies, model organisms and tools, where possible.<br><br>Have you included the information requested as detailed in our Minimum Standards Reporting Checklist?	Yes
**Availability of data and materials**<br><br>All datasets and code on which the conclusions of the paper rely must be either included in your submission or deposited in publicly available repositories (where available and ethically appropriate), referencing such data using a unique identifier in the references and in the "Availability of Data and Materials" section of your manuscript.<br><br>Have you have met the above requirement as detailed in our Minimum Standards Reporting Checklist?	Yes

Manuscript Click here to access/download;Manuscript;223-HL.docx

De novo genome assembly and Hi-C analysis reveal an association between chromatin architecture alterations and sex differentiation in the woody plant *Jatropha curcas*

Mao-Sheng Chen[1, 2, †], Longjian Niu[3, 5, †], Mei-Li Zhao[1, 4], Chuanjia Xu[1, 4], Bang-Zhen Pan[1, 2], Qiantang Fu[1, 2], Yan-Bin Tao[1, 2], Huiying He[1, 2], Chunhui Hou[3, *], Zeng-Fu Xu[1, 2, *]

[1] CAS Key Laboratory of Tropical Plant Resources and Sustainable Use, Xishuangbanna Tropical Botanical Garden, The Innovative Academy of Seed Design, Chinese Academy of Sciences, Menglun, Mengla, Yunnan 666303, China

[2] Center of Economic Botany, Core Botanical Gardens, Chinese Academy of Sciences, Menglun, Mengla, Yunnan 666303, China

[3] Department of Biology, Southern University of Science and Technology, Shenzhen, Guangdong 518055, China

[4] College of Life Sciences, University of Chinese Academy of Sciences, Beijing 100049, China

[5] Department of Biology, Nankai University, Tianjin, 660885, China

* Correspondence: houch@sustech.edu.cn; zfxu@xtbg.ac.cn

† Mao-Sheng Chen and Longjian Niu contributed equally to this work

Mao-Sheng Chen, ORCID: 0000-0001-5038-2287; E-mail: chenms@xtbg.org.cn
Longjian Niu, ORCID: 0000-0002-2545-3669; E-mail: niulongjian@126.com
Mei-Li Zhao, ORCID: 0000-0001-5013-4259; E-mail: zhaomeili@xtbg.ac.cn
Chuanjia Xu, ORCID: 0000-0001-5304-0952; E-mail: xuchuanjia@xtbg.ac.cn
Bang-Zhen Pan, ORCID: 0000-0002-7594-7966; E-mail: pbz@xtbg.org.cn
Qiantang Fu, ORCID: 0000-0002-3401-0413; E-mail: qtfu2002@163.com
Yan-Bin Tao, ORCID: 0000-0002-3855-9761; E-mail: taoyanbin@xtbg.ac.cn
Huiying He, ORCID: 0000-0002-9522-9013; E-mail: hhy@xtbg.org.cn

Chunhui Hou, ORCID: 0000-0002-8339-1857; E-mail: houch@sustech.edu.cn
Zeng-Fu Xu, ORCID: 0000-0001-6045-5865; E-mail: zfxu@xtbg.ac.cn

Abstract

Background: Chromatin architecture is an essential factor regulating gene transcription in different cell types and developmental phases. However, studies on chromatin architecture in perennial woody plants and on the function of chromatin organization in sex determination have not been reported.

Results: Here, we produced a chromosome-scale *de novo* genome assembly of the woody plant *Jatropha curcas* with a total length of 379.5 Mb and a scaffold N50 of 30.7 Mb using PacBio long reads combined with genome-wide chromosome conformation capture (Hi-C) technology. Based on this high-quality reference genome, we detected chromatin architecture differences between monoecious and gynoecious inflorescence buds of *Jatropha*. Differentially expressed genes (DEGs) were significantly enriched in the changed A/B compartments and topologically associated domain (TAD) regions and occurred preferentially in differential contact regions between monoecious and gynoecious inflorescence buds. Twelve DEGs related to flower development or hormone synthesis displayed significantly different genomic interaction patterns in monoecious and gynoecious inflorescence buds. These results demonstrate that chromatin organization participates in the regulation of gene transcription during the process of sex differentiation in *Jatropha.*

Conclusions: We have revealed the features of chromatin architecture in perennial woody plants and investigated the possible function of chromatin organization in *Jatropha* sex differentiation. These findings will facilitate understanding of the regulatory mechanisms of sex determination in higher plants.

Keywords: high-quality genome, Hi-C, sex determination, chromatin architecture, *Jatropha*

Introduction

Flowering plants have extremely diverse reproductive systems that are controlled by both genetic factors and environmental cues [1]. For optimal outcrossing and efficient resource allocation, approximately 10% of angiosperm species have evolved reproductive systems with unisexual flowers, in which the male and female reproductive organs are physically separated; these plant taxa are termed dioecious or monoecious [2, 3]. Sex determination has evolved independently multiple times, and various regulatory mechanisms control this process [4-6]. During sex determination in cucumber, the *femaleness* (*F*) locus controls the degree of femaleness, the *androecious* (*A*) locus promotes maleness, and the *andromonoecious* (*M*) locus is responsible for the selective abortion of stamens [7]. The *F* locus has been linked to the 1-aminocyclopropane-1 carboxylic acid synthase (*CsACS1*) gene, which occurs as a single copy in monoecious lines but is duplicated in gynoecious lines [8, 9], while the *M* locus has been linked to the *CsACS2* gene, and a conserved residue conversion (Gly33Cys) in *CsACS2* causes the generation of bisexual flowers in cucumber [10]. The *andromonoecious* (*a*) and *gynoecious* (*g*) loci control sex determination in melon [11]. The *a* locus has been linked to the *GmACS-7* gene, and loss-of-function of *GmACS-7* causes male organ development, generating andromonoecious plants [12]; the *g* locus encodes a repressor of carpel development, CmWIP1, activation of which causes a transition from male to female flowers in gynoecious plants [13]. In addition, CmACS-11 inhibits the expression of *CmWIP1*, and loss-of-function of *CmACS-11* results in a transition from monoecious to androecious individuals [14]. In *Diospyros,* an autosomal *MeGI* gene regulates anther fertility, and a Y-chromosome *OGI* gene encodes a small RNA that suppresses the expression of *MeGI*, resulting in the generation of androecious individuals [15]. In maize, the *tasselseed1* (*ts1*) gene encodes a lipoxygenase involved in jasmonic acid (JA) biosynthesis, and the *ts1* mutant has

defective stamen development because of a low JA concentration [16]. *tasselseed2* (*ts2*) encodes a short-chain alcohol dehydrogenase and is required for the arrest of pistil primordium development [17]. *tasselseed4* (*ts4*) encodes a microRNA, miR172, that targets *Tasselseed6* (*Ts6*)/*indeterminate spikelet1* (*ids1*), and both *ts2* and *ts4* are essential for suppression of carpel development [18]. *nana plant1* (*na1*) encodes a 5α-steroid reductase involved in brassinosteroid (BR) biosynthesis, and the *na1* mutant displays dwarf and feminized phenotypes [19]. In addition, exogenous application of auxin, BR, cytokinin (CK), ethylene (ETH), gibberellin (GA), JA, and their inhibitors also affects sexual expression in several species [7, 20-23]. Temperature, photoperiod, nutrition, drought, pH, and seasonality further influence sex differentiation, and epigenetic mechanisms are likely involved in the process [24-27]. In *Jatropha*, treatment with 6-benzyladenine (BA, a synthetic compound with CK activity) significantly increases the number of female flowers, in which a *SUPERMAN* orthologue (*JcSUP*) is upregulated, while *TASSELSEED2* orthologue (*JcTS2*) is downregulated [28-30]. Treatment with paclobutrazol, a GA biosynthesis inhibitor, downregulates the expression of the orthologous genes *JcHUA1*, *NO POLLEN GERMINATION-RELATED 2* (*JcNPGR2*), *MALE GAMETOPHYTE DEFECTIVE 2* (*JcMGP2*), and *JcMGP3* and increases the number of female flowers in *Jatropha* [31]. As shown by the above findings, sex differentiation is a complicated process that is mediated by both genetic and environmental factors, and the regulatory mechanisms of sex differentiation are diverse among various species.

Eukaryotic chromatin is packed into highly ordered and hierarchical structures, which contributes to the regulation of gene expression in different cell types and developmental phases [32, 33]. This well-ordered three-dimensional (3D) chromatin architecture is essential for gene transcription, DNA replication, and genome integrity [34-36]. According to genome-wide interaction patterns, each chromosome can be partitioned into three hierarchical chromatin structures: A/B compartments, topologically associated domains (TADs) and chromatin loops [36-39]. The A/B compartments are associated with euchromatic (active) and heterochromatic (inactive)

chromatin regions in which genomic and epigenetic features are distinct [37]. TADs are predominant chromatin structural units, and local interactions occur with far greater frequency within TADs than at the boundary between two TADs [36, 40]. TADs can spatially confine the interactions between promoters and distal regulatory elements, facilitating the activation of transcription, and are well correlated with markers of chromatin activity [35, 38]. Chromatin loops bring genes and their regulatory elements, such as enhancers and promoters, into close proximity for direct interactions [33, 41]. Multiple enhancer-promoter combinations can share binding of common transcription factors to establish a chromatin environment more permissive to transcription than that created by a single enhancer-promoter combination [42]. In plants, similar chromatin architectures have been identified in the genomes of several crop species, such as rice, maize, tomato, sorghum and foxtail millet, but they are not conserved across these species, suggesting that chromatin organizations are complex and unique in higher plants [43, 44].

Jatropha curcas L. (NCBI:txid180498), a perennial woody plant, is known as a potential biofuel crop because of its high seed oil content [45, 46]. A member of the spurge family native to the American tropics, common names in include the physic nut, wild castor, or bubble bush. At present, four different *Jatropha* genome assemblies have been reported [47-50], but they are insufficient to meet the requirements of chromatin architecture analysis, which requires a high-quality reference genome. *Jatropha* has two different ecotypes, monoecious and gynoecious. Monoecious plants bear male and female flowers separately on the same inflorescence; in contrast, gynoecious plants bear only female flowers, as their male flowers are aborted at an early stage of inflorescence development [51, 52]. In this study, we produced a chromosome-scale *Jatropha* assembly using a combination of single-molecule PacBio sequencing and genome-wide chromosome conformation capture (Hi-C) technology [53, 54]. Based on this high-quality reference genome, we investigated the function of chromatin architecture during sex differentiation by comparing chromatin architectures and transcriptomes between monoecious and gynoecious *Jatropha* inflorescence buds. Our results will facilitate the

elucidation of sex determination in *Jatropha* and clarify the biological functions of chromatin architecture in higher plants.

Results

Chromosome-scale *Jatropha* genome assembly

PacBio long-read sequencing data (33.41 Gb) were used for *de novo* assembly of the *Jatropha* genome (Additional Figure S1). The sequence coverage was approximately 80× based on the genome size (416 Mb), as estimated with flow cytometry [55]. The first round of genome assembly was performed using the FALCON package (version 0.3.0) [56], and then polishing was performed using the arrow algorithm in Pacific SMRT Link (version 5.1.0). The assembly was composed of 1,265 contigs with a total length of 378.3 Mb and an N50 value of 1.0 Mb (Table 1). The three-dimensional proximity information obtained via the Hi-C sequencing data was used to correct instances of misjoining and to order and orient the contig assembly; then, the results were integrated into a candidate chromosome-scale assembly using the 3D *de novo* assembly (3D DNA) pipeline [54]. The candidate assembly was further improved by interactive correction using Juicebox Assembly Tools [57]. The final *Jatropha* assembly (hereafter referred to as our *Jatropha* assembly) had a total length of 379.1 Mb and an N50 value of 30.7 Mb and contained 11 complete chromosomes (each chromosome > 27.1 Mb) (Table 1). After masking of repetitive sequences, 25,817 protein-coding genes were predicted based on transcript and protein alignments using the MAKER annotation pipeline (version 2.31.10) [58, 59] (Table 1). The annotation of our *Jatropha* assembly had a high annotation edit distance (AED) score (Additional Figure S2) [60], suggesting that it was a high-quality genome annotation.

Quality evaluation of the new *Jatropha* assembly

We calculated small local errors in the new *Jatropha* assembly, such as single-base substitutions, short insertions and deletions, with PacBio long-read alignments using

the arrow algorithm in PacBio SMRT Link (version 5.1.0). The estimated error rate was 0.22% (substitutions, 0.17%; insertions, 0.03%; and deletions, 0.02%). However, the actual error rate should be far smaller than the estimated rate, because a large number of false errors could have been introduced into the genome sequence by the heterozygosity of the *Jatropha* genome. The completeness and contiguity were assessed using the QUAST-LG, BUSCO (version 3.0), mummer (version 4.0) and MCScanX packages [61-64]. The BUSCO results showed that our assembly was more complete than the published *Jatropha* genome assemblies (Figure 1A) [47-49]. Comparison of the genome sequences showed that our assembly and the published *Jatropha* assemblies had similar genomic structures (Figure 1B) [47-50], but the completeness and contiguity of our *Jatropha* assembly were better than those of the other assemblies (Figure 1A, 1D and 1E, Additional Table S1 and Additional Figure S3). Moreover, we compared Hi-C interaction maps across our assembly and the previous *Jatropha* assemblies by mapping Hi-C sequencing reads to the respective reference genomes, and our *Jatropha* assembly displayed perfect completeness and contiguity in this analysis (Figure 1C and Figure 1E).

Features of chromatin architecture in the new *Jatropha* genome

We investigated the chromatin architecture of our *Jatropha* genome with the Hi-C method [37]. Three types of Hi-C libraries were constructed: "m-bud" Hi-C libraries from monoecious inflorescence buds, "m-leaf" Hi-C libraries from monoecious leaves, and "g-bud" Hi-C libraries from gynoecious inflorescence buds. Two biological replicates per sample were generated (Additional Table S2). The biological replicates had a high correlation coefficient (Additional Figure S4). Three two-dimensional contact maps were generated to display the chromatin architectures of the m-bud, g-bud and m-leaf samples (Additional Figure S5). Each chromosome region was partitioned into alternating positive and negative eigenvectors representing the A/B compartments using principal component analysis (PCA) (Figure 2B and Additional Figure S6). The average number of protein-coding genes in the A compartment regions

was significantly higher than that in the B compartment regions (Figure 2C); the A and B compartments correspond to euchromatic and heterochromatic regions, which are the important chromatin structural units in both animals and plants [37, 39, 65]. The local differences in the A/B compartments in the g-bud vs. m-bud and m-leaf vs. m-bud comparisons implied that chromatin organization was varied (Figure 2A, 2B and Additional Figure S6), which may have been associated with the different phenotypes or tissues.

TADs are principal chromatin structural units; notably, the frequency of chromatin interactions within TADs is higher than that within the boundary regions and reflects the presence of distinct and autonomously regulated regions of chromosomes [33, 34, 36, 40]. In *Jatropha*, we detected 1,055, 1,058 and 821 TAD-like domains at 10 kb resolution from the m-bud, g-bud and m-leaf samples, respectively, with the arrowhead algorithm in the Juicer pipeline [66]. The median length of the TADs was 90-110 kb (Figure 3C), and they covered approximately 30.5-46.3% of the *Jatropha* chromosomes. Great differences were observed in the TAD regions in both the g-bud vs. m-bud and m-leaf vs. m-bud comparisons (Figure 2A and 2C), implying that chromatin architecture also differs among different phenotypes or tissues in *Jatropha*. In rice, the formation of TADs may be relevant to histone modifications and gene transcription; the density of protein-coding genes is much lower in TAD interior regions than in the boundary regions [43, 44]. In *Jatropha*, gene density was significantly higher in the TAD boundary regions than in the TAD interior regions in inflorescence buds (m-bud and g-bud groups), similar to the case in rice, but no differences between leaves and buds (m-leaf and m-bud groups) were observed (Figure 3A and 3B), suggesting that TAD features vary among different sexual phenotypes of *Jatropha*.

The chromatin loop is a fine chromatin structure that brings distant DNA elements and their target genes into close proximity, facilitating transcriptional activation [41]. We detected 2,221, 2,409 and 371 chromatin loops from the contact matrices of the m-bud, g-bud and m-leaf samples, respectively (Additional Table S3), using the HiCCUPS

algorithm in the Juicer pipeline [66]. These chromatin loops were confirmed using the aggregate peak analysis (APA) algorithm in the Juicer pipeline (Additional Figure S7) [66]. Differential chromatin loops were detected in the g-bud vs. m-bud and m-leaf vs. m-bud comparisons (Additional Table S4), suggesting that chromatin loops are also varied; this variation may be relevant to different phenotypes or tissues.

Chromatin architecture plays important roles in the regulation of gene expression during various cellular processes [65]. We monitored obvious local chromatin architecture alterations in A/B compartments, TADs and chromatin loops across the m-bud, g-bud and m-leaf samples (Figures 2, Additional Figure S6 and Additional Table S4). The results implied that chromatin organization is intimately associated with different sexual phenotypes and organ morphologies in *Jatropha*.

Differential contacts and differentially expressed genes (DEGs) between monoecious and gynoecious inflorescence buds

To further investigate the function of chromatin architecture in sex differentiation, we detected differences in chromatin interactions between gynoecious and monoecious inflorescence buds using the HiCcompare package [67]. A total of 2,425-3,036 differential contacts were identified with a false discovery rate (FDR) of ≤ 0.05 at 5-100 kb resolution (Additional Table S5). The differential contacts between g-bud and m-bud samples preferentially occurred in the altered chromatin architecture regions, while those between m-leaf and m-bud samples were enriched only in the changed A/B compartment regions (Figure 4A). These findings imply that the differential contacts are relevant to chromatin architecture alterations during *Jatropha* sex differentiation.

In addition, we identified 1,165 DEGs between gynoecious and monoecious inflorescence buds with an FDR of ≤ 0.05 and a fold change ≥ 2.0 using our published transcriptome data (Additional Figure S8, Additional Tables S6 and S7) [68]. Gene Ontology (GO) and Kyoto Encyclopedia of Genes and Genomes (KEGG) analyses showed that the "reproductive process" (GO: 0022414) and "plant hormone signal

transduction" (ath04075) functional categories were enriched for the DEGs (Additional Figures S9 and S10 and Table S8). We investigated the relationship between DEG distribution and differential contact regions and found that the promoters of 241 DEGs overlapped with 223 differential contact regions at both 5 kb and 10 kb resolutions, implying that these genes may be regulated by DNA regulatory elements located in corresponding differential contact regions (Additional Table S9). The promoter density of the DEGs was obviously higher in the differential contact regions than in the other regions (background) at 5 kb, 10 kb and 25 kb resolutions, respectively (Figure 3B), suggesting that gene transcription is linked to the differential contacts. These results were coincident with the findings that the differential contacts were associated with chromatin architecture alterations between gynoecious and monoecious inflorescence buds (Figure 4A).

Moreover, we identified 12 genes from the 241 DEGs located in the differential contact regions that are homologous to *Arabidopsis* genes involved in flower development or biosynthesis of phytohormones associated with sex differentiation in *Jatropha*. The 12 genes included *Jatropha FERONIA* (*JcFER*, *jc003891*), *GIBBERELLIN 2-OXIDASE 8* (*JcGA2OX8*, *jc021138*), *INCREASE IN BONSAI METHYLATION 1* (*JcIBM1, jc006371*), *ISOPENTENYLTRANSFERASE 5* (*JcIPT5*, *jc020647*), *JASMONIC ACID CARBOXYL METHYLTRANSFERASE* (*JcJMT*, *jc008699*), *MATRIX METALLOPROTEINASE* (*JcMMP*, *jc004196*), *RECEPTOR-LIKE KINASE IN FLOWERS 1* (*RKF1*, *jc023149*), *SUGAR TRANSPORT PROTEIN 8* (*JcSTP8*, *jc002715*), *TERPENE SYNTHASE 21* (*JcTPS21*, *jc019906*), *tRNA ISOPENTENYLTRANSFERASE 2* (*JcIPT2*, *jc006165*), *UBIQUITIN LIGASE COMPLEX SUBUNIT 1* (*JcULCS1*, *jc023230*) and *ZUSAMMEN-CA-ENHANCED 1* (*JcZCE1*, *jc021698*) (Additional Table S10). These genes displayed different genomic interaction patterns between gynoecious and monoecious inflorescence buds (Figure 4C and Additional Figure S11), suggesting that their expression may be regulated by corresponding regulatory elements during sex differentiation. For example, *JcSTP8* and *JcJMT* had different interaction loci at 5 kb resolution between the m-bud and g-bud

samples, which may have helped promote the expression of *JcSTP8* or inhibit the expression of *JcJMT* (Figure 7 and Additional Table S7). In *Arabidopsis*, STP8 contributes to the uptake of glucose during pollen development and pollen tube growth [69, 70], and JMT catalyses the formation of methyljasmonate from JA [71]. *JcSTP8* and *JcJMT*, together with the other genes identified in this study, could participate in *Jatropha* sex differentiation, during which chromatin organization may regulate their transcription.

Distribution of DEGs and co-expressed genes in chromatin architecture units

We performed co-expression analyses of the transcriptome data for different *Jatropha* phenotypes and tissues using the WGCNA package (version 1.46) (Additional Table S11)[72-74] and detected three modules, MEgreen, MEdarkgreen and MElightcyan (Additional Figure S12, Additional Tables S12 and S13). The genes in these modules were enriched for the "reproductive process" function category, implying that they may be related to sex differentiation in *Jatropha*. We computed the distribution of DEGs and co-expressed genes in common and different regions of the chromatin architecture between g-bud and m-bud samples, respectively. The results showed that DEGs were significantly enriched in the altered A/B compartment and TAD regions, but co-expressed genes were not (Figure 5), suggesting that the DEGs were associated with chromatin architecture alteration and that the co-expressed genes are irrelevant to chromatin organization during sex differentiation in *Jatropha*.

Discussion

Chromatin organization is an important factor regulating gene transcription in many cellular processes, and dynamic alterations in chromatin architecture plays vital roles in responses to environmental stimuli [43, 65, 75-77]. The 3D structure of each chromosome contains three hierarchical functional substructure units: A/B compartments, TADs and chromatin loops [36-39]. In *Jatropha*, the same hierarchical chromatin substructures were found in the nucleus with the Hi-C approach, as they have

been in *Arabidopsis* and several crop species [43, 44, 65], implying that these chromatin architectures are widely present in plants. The structural features of TADs are well conserved among species, cell types, and tissues in mammals [36, 40, 78], but not in plants; the lack of conservation in plants might be because of the absence of the CTCF protein that is highly enriched at TAD borders in mammalian systems [36, 79]. This non-conservation of chromatin architecture may contribute to adaptations of plants in response to various environmental conditions. The observation of dynamic alterations in chromatin architecture across the m-bud, g-bud and m-leaf samples suggests that chromatin organization is associated with different sexual phenotypes or organ morphologies in *Jatropha*.

Through examination of both DEGs and differential contacts between gynoecious and monoecious inflorescence buds, twelve genes involved in *Jatropha* sex differentiation were identified, the expression of which may be regulated by corresponding DNA regulatory elements. In *Arabidopsis*, *IBM1* encodes a histone demethylase suppressing DNA methylation and gene silencing, and the *ibm1* mutant displays developmental defects [80, 81]. *RKF1* is highly expressed in early flower primordia and during stamen development [82]. *ULCS1* encodes a WD40 repeat protein, RNAi-mediated silencing of which produces sterile plants with pleiotropic phenotypes [83]. *TPS21* is a sesquiterpene synthase gene expressed in stigmas, anthers and sepals, which is responsible for the formation of floral volatile sesquiterpenes [84]. *ZCE1* encodes a member of the major latex protein-like gene family that plays a role in promoting vegetative growth and delaying flowering [85]. In *Jatropha*, the expression of *JcIBM1*, *JcRKF1*, *JcULCS1*, *JcTPS21*, *JcZCE1* and *JcSTP8* was upregulated in gynoecious inflorescence buds (Additional Table S6). *MMP* is a member of the matrix metalloproteinase gene family, and the *Arabidopsis mmp-1* mutant displays late flowering and early senescence phenotypes [86]. *FER* encodes a plasma membrane receptor protein kinase that regulates reproductive growth [52]. *GA2OX8* encodes a GA 2-oxidase that participates in the GA biosynthetic process [87]. The expression of *JcMMP*, *JcFER*, *JcGA2OX8* and *JcJMT* was downregulated in gynoecious

inflorescence buds (Additional Table S6) in the current study. Moreover, in *Arabidopsis*, *IPT2* and *IPT5* encode CK synthases that catalyse the first step in CK biosynthesis [88]. In *Arabidopsis* ATP/ADP IPTs (IPT1 and IPT3–IPT8) are responsible for isopentenyladenine- and *trans*-zeatin (*t*Z)-type CK synthesis, while tRNA IPTs (IPT2 and IPT9) are responsible for *cis*-zeatin (*c*Z)-type CK synthesis [88]. In *Jatropha*, *JcIPT2* was upregulated in gynoecious inflorescence buds, while *JcIPT5* was downregulated, suggesting that different types of CKs may play different roles in *Jatropha* sex differentiation even through exogenous CK treatment has been found to improve the production of female flowers [28-30]. These genes displayed different genomic interaction patterns between gynoecious and monoecious inflorescence buds (Figure 4C and Additional Figure S11), suggesting that their transcriptional activity may be associated with chromatin organization during sex differentiation in *Jatropha*.

Conclusions

In this study, we obtained a chromosome-level *de novo* assembly of the *Jatropha* genome using PacBio sequencing combined with Hi-C technology. Based on this high-quality reference genome, we first revealed the features of chromatin architecture in perennial woody plants and investigated the possible function of chromatin organization in sex differentiation in *Jatropha*, which will facilitate understanding of the regulatory mechanisms of sex determination in higher plants.

Methods

Plant materials

Two-year-old gynoecious and monoecious *Jatropha curcas* plants were grown in the field at the Xishuangbanna Tropical Botanical Garden of the Chinese Academy of Sciences, Yunnan Province, China. Inflorescence buds and leaves from gynoecious and monoecious plants were fixed for Hi-C library construction. Leaves of monoecious plants were frozen for PacBio sequencing. Two biological replicates per sample were

generated for Hi-C library construction.

PacBio sequencing and *de novo* assembly

PacBio sequencing was performed on a PacBio Sequel sequencer by Novogene Bioinformatics Technology (Beijing, China). After the polymerase reads were filtered (minReadScore = 0.8), the filtered subreads were used for first-round assembly using the FALCON package (Falcon, RRID: SCR_016089; version 0.3.0) with the following parameters: length cutoff = 1000, seed coverage = 35 and length cutoff pre-assembly = 11000 [56]. The contig sequences produced were corrected with PacBio sequencing data using the arrow algorithm in PacBio SMRT Link (version 5.1.0) (www.pacb.com). Along with the Hi-C sequencing data, the contig sequences were then integrated into a candidate chromosome-scale assembly using a 3D DNA pipeline [54]. The candidate assembly was further corrected for the final genome sequences using Juicebox Assembly Tools (Version 1.8.9) [57].

Genome annotation and quality evaluation

After masking repetitive sequences based on a custom repeat library with the RepeatModeler package (RepeatModeler, RRID: SCR_015027) (www.repeatmasker.org), the assembly of monoecious *Jatropha* was annotated using the MAKER genome annotation pipeline (MAKER, RRID: SCR_005309; version 2.31.10) [58, 59]. Both transcript and protein sequences were used for *ab initio* gene prediction. The transcript sequences were *de novo* assembled with our previous transcriptome sequencing data (SRP092157) and NCBI RefSeq *Jatropha* transcript data using Trinity (Trinity, RRID: SCR_013048; version 2.2.0) with the default parameters [89, 90]. The protein sequences were from the Ensembl Plants database (Ensembl Plants, RRID: SCR_008680) (http://plants.ensembl.org). The SNAP and AUGUSTUS programs in the MAKER pipeline were used to train the gene prediction model [91, 92]. A detailed description of the MAKER pipeline is provided on the MAKER Wiki page[93]. The AED algorithm was used for assembly annotation [60]. The QUAST-LG

(RRID: SCR_001228), BUSCO (RRID: SCR_015008; version 3.0), mummer (RRID: SCR_001200; version 4.0) and MCScanX packages were used to assess assembly completeness and contiguity [61-64]. Single-base substitutions and short insertions and deletions in the assembly were estimated with PacBio long-read alignments using the arrow algorithm in Pacific SMRT Link (version 5.1.0). A visual Hi-C-based chromatin interaction map approach was used to assess misassemblies, such as structural errors, using Juicebox Assembly Tools (version 1.8.9) [57].

Hi-C library preparation

The Hi-C protocol was adapted for library construction as previously described [94]. Plant materials were fixed with 2% formaldehyde solution at room temperature for 30 min in a vacuum. Then, 2.5 M glycine was added to quench the crosslinking reaction. Approximately 0.5 g of fixed tissue was ground with liquid nitrogen for DNA isolation. The extracted nuclei were resuspended with 0.5% SDS and incubated at 62 °C for 5 min. Then, 10% Triton X-100 was added, and the samples were incubated at 37 °C for 15 min. The denatured DNA was digested with the 4-cutter restriction enzyme DpnII at 37 °C overnight. The DpnII enzyme was inactivated at 62 °C for 20 min. Next, the digested DNA was blunt-ended by filling in of nucleotides with the Klenow enzyme at 37 °C for 2 h. The proximal chromatin DNA was religated with T4 DNA ligase at room temperature for 4 h. After centrifugation at 1,000 ×*g* for 3 min, the reaction mixture was resuspended with SDS buffer (50 mM Tris-HCl, 1% SDS, 10 mM EDTA, pH 8.0), proteinase K was added, and the mixture was incubated at 55 °C for 30 min. Formaldehyde crosslinking of nuclear complexes was reversed by addition of 30 µl of 5 M NaCl and incubation at 65 °C overnight. Subsequent manipulations were carried out as previously described [94]. The final Hi-C library sequencing was performed on an Illumina HiSeq-2500 platform with 150 bp paired-end reads.

Hi-C data analysis pipeline

Analysis of the Hi-C sequencing data was performed using the Juicer pipeline (Juicer, RRID: SCR_017226) [66]. Duplicate and near-duplicate reads mapped to the

same restriction fragment were removed and then filtered with mapping quality scores. The contact matrices were normalized at different resolutions. Eigenvectors were identified with the eigenvector algorithm, the sign of which indicated whether the reads were in compartment A or compartment B; TADs were identified with the arrowhead algorithm; and chromatin loops were identified with the HiCCUPS algorithm. The aggregate enrichment of putative peaks in contact matrices was validated with the APA algorithm as described previously [38]. Differential chromatin contacts between the contact matrices were identified using the HiCcompare R package (version 1.8.0) [67]. All Hi-C maps were generated using Juicebox Assembly Tools (version 1.8.9) [57]. Correlation analysis was performed using the corrplot R package (Version 0.85) [95].

Analysis of DEGs and co-expressed genes

Our previous transcriptome data were reanalysed to examine DEGs between monoecious and gynoecious inflorescence buds. The sequencing reads were mapped to the new *Jatropha* reference genome using the Subread package (Subread, RRID: SCR_009803; version 1.6.2) with the default parameters [96, 97]. DEGs with an FDR of ≤ 0.05 and an expression fold change ≥ 2.0 were identified using the edgeR package (edgeR, RRID: SCR_012802) [98]. Co-expressed gene analysis was performed using the WGCNA R package (RRID: SCR_003302; version 1.46) [74]. The expression counts of all samples were $\log_2(x+1)$-transformed, and batch effects were removed using the ComBat function in the SVA package (RRID: SCR_002155; version 3.34.0) [99]. GO and KEGG annotation were performed with the Database for Annotation, Visualization and Integrated Discovery (DAVID) (RRID: SCR_001881; version 6.8) [100].

Availability of supporting data and materials

All high-throughput sequencing reads and the assembly presented in the manuscript have been submitted to the China National GeneBank (CNGB) Nucleotide Sequence Archive (CNSA) under accession number CNP0000449. Raw data is also

available and clustered together under NCBI bioproject PRJNA415534. In this study, the data for CNR0106032-CNR0106034 were from the CNGB under accession number CNP0000603; the data from SRR10076311-SRR10076316, SRR10076310 and SRR10076325 were from the NCBI under accession number SRP220547; the data for SRR1565783-SRR1565786, SRR1565789-SRR1565790, and SRR1565797-SRR1565780 were from the NCBI under accession number SRR1565797; the data for SRR4473569-SRR4473570, SRR4473575, SRR4473565, and SRR4473571-SRR4473572 were from the NCBI under accession number SRP092157; and the data for SRR6227301-SRR6227302, SRR6227305-SRR6227306, SRR6227308 and SRR6227312 were from the NCBI under accession number SRP122257. All supporting data and materials are available in the *GigaScience* GigaDB database [101].

List of abbreviations

ACS1: 1-aminocyclopropane-1 carboxylic acid synthase, AED: annotation edit distance, APA: aggregate peak analysis, BR: brassinosteroid, CK: cytokinin, DEG: differentially expressed gene, ETH: ethylene, FDR: false discovery rate, FER: FERONIA, GA: gibberellin, GA2OX8: GIBBERELLIN 2-OXIDASE 8, Hi-C: genome-wide chromosome conformation capture, ids1: indeterminate spikelet1, IBM1: INCREASE IN BONSAI METHYLATION 1, IPT2: tRNA ISOPENTENYLTRANSFERASE 2, IPT5: ISOPENTENYLTRANSFERASE 5, JA: jasmonic acid, JMT: JASMONIC ACID CARBOXYL METHYLTRANSFERASE, MMP: MATRIX METALLOPROTEINASE, na1: nana plant1, RKF1: RECEPTOR-LIKE KINASE IN FLOWERS 1, STP8: SUGAR TRANSPORT PROTEIN 8, TAD: topologically associated domain, TPS21: TERPENE SYNTHASE 21, ts2: tasselseed2, ULCS1: UBIQUITIN LIGASE COMPLEX SUBUNIT 1, ZCE1: (ZUSAMMEN-CA)-ENHANCED 1

Ethics approval and consent to participate

Not applicable

Competing interests

The authors declare no conflict of interest.

Funding

This work was supported by the National Natural Science Foundation of China (31670612, 31971628, 31870291, 31300568, 31370595 and 31571347), the Programme of the Chinese Academy of Sciences (kfj-brsn-2018-6-008 and 2017XTBG-T02) and the Guangdong Science and Technology Department (2016A030313642).

Author contributions

LN, M-SC, CH, and Z-FX designed the study and wrote the paper. LN performed the Hi-C experiments. M-LZ, CX, B-ZP, QF, Y-BT, and HH carried out additional experiments. M-SC and LN analysed and interpreted the data. All authors reviewed the final manuscript.

Acknowledgements

We gratefully acknowledge the Central Laboratory of the Xishuangbanna Tropical Botanical Garden for providing high-performance computing and other research facilities.

References

1. Aryal R and Ming R. Sex determination in flowering plants: papaya as a model system. Plant Sci. 2014;217-218:56-62. doi:10.1016/j.plantsci.2013.10.018.
2. Bawa KS. Evolution of dioecy in flowering plants. Annu Rev Ecol Syst. 1980;11:15-39. doi:10.1146/annurev.es.11.110180.000311.

3. Thomson JD and Barrett SCH. Selection for outcrossing, sexual selection, and the evolution of dioecy in plants. The American Naturalist. 1981;118:443-9. doi:10.1086/283837.
4. Ainsworth C, Parker J and Buchanan-Wollaston V. Sex determination in plants. Current Topics in Developmental Biology. 1998;38:167-223. doi:10.4161/psb.2.3.3728.
5. Chuck G. Molecular mechanisms of sex determination in monoecious and dioecious plants. Adv Bot Res. 2010;54:53-83. doi:10.1016/S0065-2296(10)54002-3.
6. Diggle PK, Di Stilio VS, Gschwend AR, Golenberg EM, Moore RC, Russell JRW, et al. Multiple developmental processes underlie sex differentiation in angiosperms. Trends Genet. 2011;27 9:368-76. doi:10.1016/j.tig.2011.05.003.
7. Perl-Treves R. Male to female conversion along the cucumber shoot: approaches to studying sex genes and floral development in *Cucumis sativus*. In: Ainsworth CC, editor. Sex determination in plants. Oxford: BIOS Scientific Publishers; 1999. p. 189-286.
8. Mibus H and Tatlioglu T. Molecular characterization and isolation of the *F/f* gene for femaleness in cucumber (*Cucumis sativus* L.). Theor Appl Genet. 2004;109 8:1669-76. doi:10.1007/s00122-004-1793-7.
9. Kamachi Si, Sekimoto H, Kondo N and Sakai S. Cloning of a cDNA for a 1-aminocyclopropane-1-carboxylate synthase that is expressed during development of female flowers at the apices of *Cucumis sativus* L. Plant Cell Physiol. 1997;38 11:1197-206. doi:10.1093/oxfordjournals.pcp.a029106.
10. Li Z, Huang S, Liu S, Pan J, Zhang Z, Tao Q, et al. Molecular isolation of the *M* gene suggests that a conserved-residue conversion induces the formation of bisexual flowers in cucumber plants. Genetics. 2009;182 4:1381-5. doi:10.1534/genetics.109.104737.
11. Poole CF and Grimball PC. Inheritance of new sex forms in *Cucumis melo* L. J Hered. 1939;30 1:21-5.
12. Boualem A, Fergany M, Fernandez R, Troadec C, Martin A, Morin H, et al. A conserved mutation in an ethylene biosynthesis enzyme leads to andromonoecy in melons. Science. 2008;321 5890:836-8. doi:10.1126/science.1159023.
13. Martin A, Troadec C, Boualem A, Rajab M, Fernandez R, Morin H, et al. A transposon-induced epigenetic change leads to sex determination in melon. Nature. 2009;461 7267:1135-8. doi:10.1038/nature08498.
14. Boualem A, Troadec C, Camps C, Lemhemdi A, Morin H, Sari M-A, et al. A cucurbit androecy gene reveals how unisexual flowers develop and dioecy emerges. Science. 2015;350 6261:688-91. doi:10.1126/science.aac8370.
15. Akagi T, Henry IM, Tao R and Comai L. A Y-chromosome-encoded small RNA acts as a sex determinant in persimmons. Science. 2014;346 6209:646-50. doi:10.1126/science.1257225.
16. Acosta IF, Laparra H, Romero SP, Schmelz E, Hamberg M, Mottinger JP, et al. *tasselseed1* is a lipoxygenase affecting jasmonic acid signaling in sex determination of maize. Science. 2009;323 5911:262-5. doi:10.1126/science.1164645.
17. DeLong A, Calderon-Urrea A and Dellaporta SL. Sex determination gene *TASSELSEED2* of maize encodes a short-chain alcohol dehydrogenase required for stage-specific floral organ abortion. Cell. 1993;74 4:757-68. doi:10.1016/0092-8674(93)90522-R.
18. Chuck G, Meeley R, Irish E, Sakai H and Hake S. The maize *tasselseed4* microRNA controls sex determination and meristem cell fate by targeting *Tasselseed6/indeterminate spikelet1*. Nat Genet. 2007;39 12:1517-21. doi:10.1038/ng.2007.20.

19. Hartwig T, Chuck GS, Fujioka S, Klempien A, Weizbauer R, Potluri DP, et al. Brassinosteroid control of sex determination in maize. Proc Natl Acad Sci USA. 2011;108 49:19814-9. doi:10.1073/pnas.1108359108.
20. Louis JP and Durand B. Studies with the dioecious angiosperm *Mercurialis annua* L. (2n=16): Correlation between genic and cytoplasmic male sterility, sex segregation and feminizing hormones (cytokinins). Molecular and General Genetics MGG. 1978;165 3:309-22. doi:10.1007/bf00332532.
21. Chailakhyan MK. Genetic and hormonal regulation of growth, flowering, and sex expression in plants. Am J Bot. 1979;66 6:717-36. doi:10.2307/2442417.
22. Durand R and Durand B. Sexual differentiation in higher plants. Physiol Plant. 1984;60 3:267-74. doi:10.1111/j.1399-3054.1984.tb06061.x.
23. Irish EE and Nelson T. Sex determination in monoecious and dioecious plants. Plant Cell. 1989;1 8:737-44. doi:10.1105/tpc.1.8.737.
24. Korpelainen H. Labile sex expression in plants. Biological Reviews. 1998;73 2:157-80. doi:10.1111/j.1469-185X.1997.tb00028.x.
25. Field DL, Pickup M and Barrett SCH. Comparative analyses of sex-ratio variation in dioecious flowering plants. Evolution. 2013;67 3:661-72. doi:10.1111/evo.12001.
26. Golenberg EM and West NW. Hormonal interactions and gene regulation can link monoecy and environmental plasticity to the evolution of dioecy in plants. Am J Bot. 2013;100 6:1022-37. doi:10.3732/ajb.1200544.
27. Piferrer F. Epigenetics of sex determination and gonadogenesis. Dev Dyn. 2013;242 4:360-70. doi:10.1002/dvdy.23924.
28. Chen M-S, Pan B-Z, Wang G-J, Ni J, Niu L and Xu Z-F. Analysis of the transcriptional responses in inflorescence buds of Jatropha curcas exposed to cytokinin treatment. BMC Plant Biol. 2014;14 1:318. doi:10.1186/s12870-014-0318-z.
29. Pan B-Z, Luo Y, Song L, Chen M-S, Li J-L and Xu Z-F. Thidiazuron increases fruit number in the biofuel plant Jatropha curcas by promoting pistil development. Ind Crop Prod. 2016;81:202-1. doi:10.1016/j.indcrop.2015.11.054.
30. Seesangboon A, Pokawattana T, Eungwanichayapant PD, Tovaranonte J and Popluechai S. Effects of 6-benzyladenine on *Jatropha* gene expression and flower development. Russian Journal of Plant Physiology. 2018;65 3:345-56. doi:10.1134/s1021443718030135.
31. Seesangboon A, Gruneck L, Pokawattana T, Eungwanichayapant PD, Tovaranonte J and Popluechai S. Transcriptome analysis of *Jatropha curcas* L. flower buds responded to the paclobutrazol treatment. Plant Physiol Biochem. 2018;127:276-86. doi:https://doi.org/10.1016/j.plaphy.2018.03.035.
32. Gibcus JH and Dekker J. The hierarchy of the 3D genome. Mol Cell. 2013;49 5:773-82. doi:10.1016/j.molcel.2013.02.011.
33. Sexton T and Cavalli G. The role of chromosome domains in shaping the functional genome. Cell. 2015;160 6:1049-59. doi:https://doi.org/10.1016/j.cell.2015.02.040.
34. Sexton T, Yaffe E, Kenigsberg E, Bantignies F, Leblanc B, Hoichman M, et al. Three-dimensional folding and functional organization principles of the *Drosophila* genome. Cell. 2012;148 3:458-72. doi:https://doi.org/10.1016/j.cell.2012.01.010.
35. Jin F, Li Y, Dixon JR, Selvaraj S, Ye Z, Lee AY, et al. A high-resolution map of the three-dimensional chromatin interactome in human cells. Nature. 2013;503:290-4. doi:10.1038/nature12644.

36. Dixon JR, Selvaraj S, Yue F, Kim A, Li Y, Shen Y, et al. Topological domains in mammalian genomes identified by analysis of chromatin interactions. Nature. 2012;485:376-80. doi:10.1038/nature11082.
37. Lieberman-Aiden E, van Berkum NL, Williams L, Imakaev M, Ragoczy T, Telling A, et al. Comprehensive mapping of long-range interactions reveals folding principles of the human genome. Science. 2009;326 5950:289-93. doi:10.1126/science.1181369.
38. Rao SSP, Huntley MH, Durand NC, Stamenova EK, Bochkov ID, Robinson JT, et al. A 3D map of the human genome at kilobase resolution reveals principles of chromatin looping. Cell. 2014;159 7:1665-80. doi:https://doi.org/10.1016/j.cell.2014.11.021.
39. Miao Y and Bing R. The three-dimensional organization of mammalian genomes. Annu Rev Cell Dev Biol. 2017;33 1:265-89. doi:10.1146/annurev-cellbio-100616-060531.
40. Nora EP, Lajoie BR, Schulz EG, Giorgetti L, Okamoto I, Servant N, et al. Spatial partitioning of the regulatory landscape of the X-inactivation centre. Nature. 2012;485:381-5. doi:10.1038/nature11049.
41. Liu C and Weigel D. Chromatin in 3D: progress and prospects for plants. Genome biology. 2015;16 1:170. doi:10.1186/s13059-015-0738-6.
42. Mousavi K, Zare H, Dell'Orso S, Grontved L, Gutierrez-Cruz G, Derfoul A, et al. eRNAs promote transcription by establishing chromatin accessibility at defined genomic loci. Mol Cell. 2013;51 5:606-17. doi:https://doi.org/10.1016/j.molcel.2013.07.022.
43. Liu C, Cheng YJ, Wang JW and Weigel D. Prominent topologically associated domains differentiate global chromatin packing in rice from *Arabidopsis*. Nature plants. 2017;3 9:742-8. doi:10.1038/s41477-017-0005-9.
44. Dong Q, Li N, Li X, Yuan Z, Xie D, Wang X, et al. Genome-wide Hi-C analysis reveals extensive hierarchical chromatin interactions in rice. plant J. 2018;94 6:1141-56. doi:10.1111/tpj.13925.
45. Fairless D. Biofuel: the little shrub that could--maybe. Nature. 2007;449 7163:652-5. doi:10.1038/449652a.
46. Sato S, Hirakawa H, Isobe S, Fukai E, Watanabe A, Kato M, et al. Sequence analysis of the genome of an oil-bearing tree, *Jatropha curcas* L. DNA Res. 2011;18 1:65-76. doi:10.1093/dnares/dsq030.
47. Ha J, Shim S, Lee T, Kang YJ, Hwang WJ, Jeong H, et al. Genome sequence of *Jatropha curcas* L., a non-edible biodiesel plant, provides a resource to improve seed-related traits. Plant Biotechnol J. 2019;17 2:517-30. doi:doi:10.1111/pbi.12995.
48. Wu P, Zhou C, Cheng S, Wu Z, Lu W, Han J, et al. Integrated genome sequence and linkage map of physic nut (*Jatropha curcas* L.), a biodiesel plant. plant J. 2015;81 5:810-21. doi:doi:10.1111/tpj.12761.
49. Hirakawa H, Tsuchimoto S, Sakai H, Nakayama S, Fujishiro T, Kishida Y, et al. Upgraded genomic information of *Jatropha curcas* L. Plant Biotechnol. 2012;29 2:123-30. doi:10.5511/plantbiotechnology.12.0515a.
50. Kancharla N, Jalali S, Narasimham JV, Nair V, Yepuri V, Thakkar B, et al. *De Novo* sequencing and hybrid assembly of the biofuel crop *Jatropha curcas* L.: identification of quantitative trait loci for geminivirus resistance. Genes. 2019;10 1:69.
51. Rincon-Rabanales M, Vargas-Lopez LI, Adriano-Anaya L, Vazquez-Ovando A, Salvador-Figueroa M and Ovando-Medina I. Reproductive biology of the biofuel plant *Jatropha curcas* in its center of origin. PeerJ. 2016;4:e1819. doi:10.7717/peerj.1819.
52. Haruta M, Gaddameedi V, Burch H, Fernandez D and Sussman MR. Comparison of the effects of a

kinase-dead mutation of FERONIA on ovule fertilization and root growth of *Arabidopsis*. FEBS Lett. 2018;592 14:2395-402. doi:doi:10.1002/1873-3468.13157.

53. Burton JN, Adey A, Patwardhan RP, Qiu R, Kitzman JO and Shendure J. Chromosome-scale scaffolding of *de novo* genome assemblies based on chromatin interactions. Nat Biotechnol. 2013;31 12:1119-25. doi:10.1038/nbt.2727.
54. Dudchenko O, Batra SS, Omer AD, Nyquist SK and Hoeger M. *De novo* assembly of the *Aedes aegypti* genome using Hi-C yields chromosome-length scaffolds. Science. 2017;356 6333:92-5. doi:10.1126/science.aal3327.
55. Carvalho CR, Clarindo WR, Praça MM, Araújo FS and Carels N. Genome size, base composition and karyotype of *Jatropha curcas* L., an important biofuel plant. Plant Sci. 2008;174 6:613-7. doi:https://doi.org/10.1016/j.plantsci.2008.03.010.
56. Chin CS, Peluso P and Sedlazeck FJ. Phased diploid genome assembly with single-molecule real-time sequencing. Nat Methods. 2016;13 12:1050-4. doi:10.1038/nmeth.4035.
57. Durand NC, Robinson JT, Shamim MS, Machol I, Mesirov JP, Lander ES, et al. Juicebox provides a visualization system for Hi-C contact maps with unlimited zoom. Cell systems. 2016;3 1:99-101. doi:10.1016/j.cels.2015.07.012.
58. Campbell MS, Holt C, Moore B and Yandell M. Genome annotation and curation using MAKER and MAKER-P. Current protocols in bioinformatics. 2014;48 1:4.11.1-4..39. doi:10.1002/0471250953.bi0411s48.
59. Cantarel BL, Korf I, Robb SM, Parra G, Ross E, Moore B, et al. MAKER: an easy-to-use annotation pipeline designed for emerging model organism genomes. Genome Res. 2008;18 1:188-96. doi:10.1101/gr.6743907.
60. Eilbeck K, Moore B, Holt C and Yandell M. Quantitative measures for the management and comparison of annotated genomes. BMC Bioinformatics. 2009;10 1:67. doi:10.1186/1471-2105-10-67.
61. Mikheenko A, Prjibelski A, Saveliev V, Antipov D and Gurevich A. Versatile genome assembly evaluation with QUAST-LG. Bioinformatics. 2018;34 13:i142-i50. doi:10.1093/bioinformatics/bty266.
62. Simão FA, Waterhouse RM, Ioannidis P, Kriventseva EV and Zdobnov EM. BUSCO: assessing genome assembly and annotation completeness with single-copy orthologs. Bioinformatics. 2015;31 19:3210-2. doi:10.1093/bioinformatics/btv351.
63. Kurtz S, Phillippy A, Delcher AL, Smoot M, Shumway M, Antonescu C, et al. Versatile and open software for comparing large genomes. Genome Biology. 2004;5 2:R12. doi:10.1186/gb-2004-5-2-r12.
64. Wang Y, Tang H, DeBarry JD, Tan X, Li J, Wang X, et al. MCScanX: a toolkit for detection and evolutionary analysis of gene synteny and collinearity. Nucleic Acids Res. 2012;40 7:e49-e. doi:10.1093/nar/gkr1293.
65. Doğan ES and Liu C. Three-dimensional chromatin packing and positioning of plant genomes. Nature plants. 2018;4 8:521-9. doi:10.1038/s41477-018-0199-5.
66. Durand NC, Shamim MS, Machol I, Rao SSP, Huntley MH, Lander ES, et al. Juicer provides a one-click system for analyzing loop-resolution Hi-C experiments. Cell systems. 2016;3 1:95-8. doi:https://doi.org/10.1016/j.cels.2016.07.002.
67. Stansfield JC, Cresswell KG, Vladimirov VI and Dozmorov MG. HiCcompare: an R-package for joint normalization and comparison of HI-C datasets. BMC Bioinformatics. 2018;19 1:279.

doi:10.1186/s12859-018-2288-x.
68. Chen M-S, Pan B-Z, Fu Q, Tao Y-B, Martínez-Herrera J, Niu L, et al. Comparative transcriptome analysis between gynoecious and monoecious plants identifies regulatory networks controlling sex determination in *Jatropha curcas*. Front Plant Sci. 2017;7:1953. doi:10.3389/fpls.2016.01953.
69. Rottmann T, Klebl F, Schneider S, Kischka D, Rüscher D, Sauer N, et al. Sugar transporter STP7 specificity for L-arabinose and D-xylose contrasts with the typical hexose transporters STP8 and STP12. Plant Physiol. 2018;176 3:2330-50. doi:10.1104/pp.17.01493.
70. Rottmann T, Fritz C, Sauer N and Stadler R. Glucose uptake via STP transporters inhibits in vitro pollen tube growth in a HEXOKINASE1-dependent manner in *Arabidopsis thaliana*. The Plant Cell. 2018;30 9:2057-81. doi:10.1105/tpc.18.00356.
71. Seo HS, Song JT, Cheong J-J, Lee Y-H, Lee Y-W, Hwang I, et al. Jasmonic acid carboxyl methyltransferase: a key enzyme for jasmonate-regulated plant responses. Proc Natl Acad Sci USA. 2001;98 8:4788-93.
72. Chen M-S, Zhao M-L, Wang G-J, He H-Y, Bai X, Pan B-Z, et al. Transcriptome analysis of two inflorescence branching mutants reveals cytokinin is an important regulator in controlling inflorescence architecture in the woody plant Jatropha curcas. BMC Plant Biol. 2019;19:468. doi:10.1186/s12870-019-2069-3.
73. Sapeta H, Lourenço T, Lorenz S, Grumaz C, Kirstahler P, Barros PM, et al. Transcriptomics and physiological analyses reveal co-ordinated alteration of metabolic pathways in Jatropha curcas drought tolerance. J Exp Bot. 2015;67 3:845-60. doi:10.1093/jxb/erv499.
74. Langfelder P and Horvath S. WGCNA: an R package for weighted correlation network analysis. BMC Bioinformatics. 2008;9 1:559-. doi:10.1186/1471-2105-9-559.
75. Probst AV and Mittelsten Scheid O. Stress-induced structural changes in plant chromatin. Curr Opin Plant Biol. 2015;27:8-16. doi:https://doi.org/10.1016/j.pbi.2015.05.011.
76. Li L, Lyu X, Hou C, Takenaka N, Nguyen Huy Q, Ong C-T, et al. Widespread rearrangement of 3D chromatin organization underlies polycomb-mediated stress-induced silencing. Mol Cell. 2015;58 2:216-31. doi:https://doi.org/10.1016/j.molcel.2015.02.023.
77. Rosa S and Shaw P. Insights into chromatin structure and dynamics in plants. Biology. 2013;2 4:1378.
78. Vietri Rudan M, Barrington C, Henderson S, Ernst C, Odom Duncan T, Tanay A, et al. Comparative Hi-C reveals that CTCF underlies evolution of chromosomal domain architecture. Cell Reports. 2015;10 8:1297-309. doi:https://doi.org/10.1016/j.celrep.2015.02.004.
79. Dong P, Tu X, Chu P-Y, Lü P, Zhu N, Grierson D, et al. 3D chromatin architecture of large plant genomes determined by local A/B compartments. Molecular plant. 2017;10 12:1497-509. doi:https://doi.org/10.1016/j.molp.2017.11.005.
80. Wang Y, Xue X, Zhu J-K and Dong J. Demethylation of ERECTA receptor genes by IBM1 histone demethylase affects stomatal development. Development. 2016;143 23:4452-61. doi:10.1242/dev.129932.
81. Inagaki S, Takahashi M, Hosaka A, Ito T, Toyoda A, Fujiyama A, et al. Gene‐body chromatin modification dynamics mediate epigenome differentiation in *Arabidopsis*. The EMBO Journal. 2017;36 8:970-80. doi:10.15252/embj.201694983.
82. Takahashi T, Mu J-H, Gasch A and Chua N-H. Identification by PCR of receptor-like protein kinases from *Arabidopsis* flowers. Plant Mol Biol. 1998;37 4:587-96. doi:10.1023/a:1005924817190.
83. Beris D, Kapolas G, Livanos P, Roussis A, Milioni D and Haralampidis K. RNAi-mediated silencing of

the *Arabidopsis thaliana ULCS1* gene, encoding a WDR protein, results in cell wall modification impairment and plant infertility. Plant Sci. 2016;245:71-83. doi:https://doi.org/10.1016/j.plantsci.2016.01.008.
84. Tholl D, Chen F, Petri J, Gershenzon J and Pichersky E. Two sesquiterpene synthases are responsible for the complex mixture of sesquiterpenes emitted from Arabidopsis flowers. plant J. 2005;42 5:757-71. doi:10.1111/j.1365-313X.2005.02417.x.
85. Guo D, Wong WS, Xu WZ, Sun FF, Qing DJ and Li N. *Cis-cinnamic acid-enhanced 1* gene plays a role in regulation of *Arabidopsis* bolting. Plant Mol Biol. 2011;75 4:481-95. doi:10.1007/s11103-011-9746-4.
86. Golldack D, Popova OV and Dietz K-J. Mutation of the matrix metalloproteinase at2-MMP inhibits growth and causes late flowering and early senescence in *Arabidopsis*. J Biol Chem. 2002;277 7:5541-7. doi:10.1074/jbc.M106197200.
87. Schomburg FM, Bizzell CM, Lee DJ, Zeevaart JAD and Amasino RM. Overexpression of a novel class of gibberellin 2-oxidases decreases gibberellin levels and creates dwarf plants. The Plant Cell. 2003;15 1:151-63. doi:10.1105/tpc.005975.
88. Miyawaki K, Tarkowski P, Matsumoto-Kitano M, Kato T, Sato S, Tarkowska D, et al. Roles of *Arabidopsis* ATP/ADP isopentenyltransferases and tRNA isopentenyltransferases in cytokinin biosynthesis. Proc Natl Acad Sci USA. 2006;103 44:16598-603.
89. Grabherr MG, Haas BJ, Yassour M, Levin JZ, Thompson DA, Amit I, et al. Full-length transcriptome assembly from RNA-Seq data without a reference genome. Nat Biotechnol. 2011;29 7:644-52. doi:10.1038/nbt.1883.
90. Haas BJ, Papanicolaou A, Yassour M, Grabherr M, Blood PD, Bowden J, et al. *De novo* transcript sequence reconstruction from RNA-seq using the Trinity platform for reference generation and analysis. Nature Protocols. 2013;8 8:1494-512. doi:10.1038/nprot.2013.084.
91. Stanke M, Diekhans M, Baertsch R and Haussler D. Using native and syntenically mapped cDNA alignments to improve de novo gene finding. Bioinformatics. 2008;24 5:637-44. doi:10.1093/bioinformatics/btn013.
92. Korf I. Gene finding in novel genomes. BMC Bioinformatics. 2004;5 1:59. doi:10.1186/1471-2105-5-59.
93. MAKER Wiki. http://weatherby.genetics.utah.edu/MAKER/wiki/index.php/Main_Page
94. Wang C, Liu C, Roqueiro D, Grimm D, Schwab R, Becker C, et al. Genome-wide analysis of local chromatin packing in *Arabidopsis thaliana*. Genome Res. 2015;25 2:246-56. doi:10.1101/gr.170332.113.
95. R package "corrplot": Visualization of a Correlation Matrix. https://cran.r-project.org/web/packages/corrplot/index.html
96. Liao Y, Smyth GK and Shi W. The Subread aligner: fast, accurate and scalable read mapping by seed-and-vote. Nucleic Acids Res. 2013;41 10:e108. doi:10.1093/nar/gkt214.
97. Liao Y, Smyth GK and Shi W. featureCounts: an efficient general purpose program for assigning sequence reads to genomic features. Bioinformatics. 2014;30 7:923-30. doi:10.1093/bioinformatics/btt656.
98. Robinson MD, McCarthy DJ and Smyth GK. edgeR: a Bioconductor package for differential expression analysis of digital gene expression data. Bioinformatics. 2010;26 1:139-40. doi:10.1093/bioinformatics/btp616.
99. Leek JT, Johnson WE, Parker HS, Jaffe AE and Storey JD. The sva package for removing batch

effects and other unwanted variation in high-throughput experiments. Bioinformatics. 2012;28 6:882-3. doi:10.1093/bioinformatics/bts034.

100. Huang DW, Sherman BT and Lempicki RA. Systematic and integrative analysis of large gene lists using DAVID bioinformatics resources. Nature Protocols. 2009;4 1:44-57. doi:10.1038/nprot.2008.211.

101. Chen MS; Niu L; Zhao ML; Xu C; Pan BZ; Fu Q; Tao YB; He H; Hou C; Xu ZF (2020): Supporting data for "De novo genome assembly and Hi-C analysis reveal the association between chromatin architecture alterations and sex differentiation in the woody plant Jatropha curcas" *GigaScience* Database. http://dx.doi.org/10.5524/100689.

Figure legends

Figure 1 Genome comparison between our assembly and the published *Jatropha* assemblies. (A) BUSCO annotation of our assembly and the published *Jatropha* assemblies. n represents the number of single-copy orthologous genes. (B) Collinearity analysis of entire genome sequences between our assembly and the published *Jatropha* assemblies. (C) Comparison of Hi-C contact maps among our *Jatropha* genome assembly and the other two assemblies. The red square represents the strongest signal value. (D) Distribution of sequence length in our assembly and the published *Jatropha* assemblies. (E) Comparison of corresponding chromosomes between our assembly and the Ha et al. (2019) assembly. “jc1-11” indicates the chromosome codes of our *Jatropha* assembly, and “chr1-11” indicates the chromosome codes of the Ha et al. (2019) *Jatropha* assembly.

Figure 2 Chromatin architecture changes in the g-bud vs. m-bud and m-leaf vs. m-bud comparisons. (A) Comparison of the chromatin architecture of chromosome 3 in g-bud vs. m-bud samples and in m-leaf vs. m-bud samples. The black arrows indicate the changed A/B compartment regions, and the green arrows indicate the changed TAD regions. The legends indicate the interaction strength (observed/expected). (B) A/B compartments of chromosome 3 across m-bud, g-bud and m-leaf samples. The black box indicates the changed regions; the shaded green area indicates the B compartment region. (C) Protein-coding gene distribution in A/B compartment regions across the m-bud, g-bud and m-leaf samples. The diamond represents the mean value. A and B

represent the A compartment and B compartment, respectively. Statistical tests were carried out using the Welch two-sample t-test in R software (https://cran.r-project.org). (D) Comparison of TADs in g-bud vs. m-bud samples and in m-leaf vs. m-bud samples. The number represents the number of TADs. The label “m-bud” indicates monoecious inflorescence bud samples, the label “g-bud” indicates gynoecious inflorescence bud samples, and the label “m-leaf” indicates monoecious leaf samples.

Figure 3 Distribution of protein-coding genes around TADs across the m-bud, g-bud and m-leaf samples. (A) Gene distribution around TAD boundaries. The left area indicates TAD boundary regions, and the shaded green area indicates TAD interior regions. (**B)** Comparison of gene density between TAD boundary and interior regions. The diamond represents the mean value. Statistical tests were performed using the Welch two-sample t-test in R software. (C) Distribution of TAD sizes at 10 kb resolution in the m-bud, g-bud and m-leaf samples. The labels “m-bud”, “g-bud” and “m-leaf” indicate the same samples shown in Figure 2.

Figure 4 Differential contacts are relevant to gene transcription. (A) Enrichment analysis of the differential contacts in chromatin architecture regions. A hypergeometric distribution test was performed with the phyper function in R software. (B) Densities of DEG promoters in differential contact regions between the g-bud and m-bud samples. (C) Genomic interaction profiles of the *JcJMT* and *JcSTP8* genes in the m-bud and g-bud samples. The labels “m-bud”, “g-bud” and “m-leaf” indicate the same samples shown in Figure 2.

Figure 5 Enrichment analysis of the DEGs and the co-expressed genes in chromatin architecture regions. (A), (B) and (C) Enrichment analysis of the DEGs in A/B compartments, TAD interiors and TAD boundaries, respectively, in common and changed regions. (D), (E) and (F) Enrichment analysis of the co-expressed genes in A/B compartments, TAD interiors and TAD boundaries, respectively, in common and changed regions. A hypergeometric distribution test was performed with the phyper function in R software.

Table 1 Statistics of our *Jatropha* genome assembly.

Supplementary information

Additional Figure S1 Distribution of PacBio subread lengths.

Additional Figure S2 AED score of our *Jatropha* assembly annotation.

Additional Figure S3 Synteny analysis between our assembly and the published *Jatropha* assemblies based on gene sequences.

Additional Figure S4 Correlation analysis of Hi-C contact matrices between biological replicates. The label “m-bud” indicates monoecious inflorescence bud samples, the label “g-bud” indicates gynoecious inflorescence bud samples, and the label “m-leaf” indicates monoecious leaf samples. The number indicates the correlation coefficient.

Additional Figure S5 Hi-C contact maps of the m-bud, g-bud and m-leaf samples. (A) Genome-wide Hi-C contact maps. (B) Hi-C contact maps of chromosome 1 at 25 kb resolution (observed/expected). The blue area indicates the A compartment region, and the brown area indicates the B compartment region. The labels “m-bud”, “g-bud” and “m-leaf” indicate the same samples shown in Additional Figure S4.

Additional Figure S6 Comparison of the A/B compartments in all chromosomes among the m-bud, g-bud and m-leaf samples. The black boxes indicate the changed A/B compartment regions, the blue area indicates the A compartment region, and the brown area indicates the B compartment region. The labels “m-bud”, “g-bud” and “m-leaf” indicate the same samples shown in Additional Figure S4.

Additional Figure S7 APA of Hi-C contact matrices across m-bud, g-bud and m-leaf samples. The labels “m-bud”, “g-bud” and “m-leaf” indicate the same samples shown in Additional Figure S4.

Additional Figure S8 DEGs identified from the g-bud vs. m-bud comparison. The blue lines indicate genes with a two-fold expression change; the red points indicate

significant DEGs with FDRs < 0.05. FC, fold change; CPM, counts per million mapped reads.

Additional Figure S9 GO enrichment analysis of the DEGs. The asterisk indicates the “reproductive process” function category.

Additional Figure S10 KEGG enrichment analysis of the DEGs.

Additional Figure S11 Genomic interaction profiles of ten DEGs in the m-bud and g-bud samples. The labels “m-bud” and “g-bud” indicate the same samples shown in Additional Figure S4.

Additional Figure S12 Co-expression analysis and GO annotation. (A) Correlation analysis between modules and biological traits (phenotype and tissue) was performed using WGCNA. The right coloured bar indicates the correlation coefficient. The numbers in each coloured cell indicate the correlation coefficient and the corresponding P-value (numbers in brackets), calculated using the WGCNA package. The asterisks indicate the MEgreen, MEdarkgreen and MElightcyan modules. (B) GO enrichment analysis of the co-expressed genes in the MEgreen, MEdarkgreen and MElightcyan modules. The asterisk indicates the “reproductive process” function category.

Additional Table S1 Statistics of our assembly and the published *Jatropha* assemblies.

Additional Table S2 Statistics of the Hi-C data of the m-bud, g-bud and m-leaf samples.

Additional Table S3 Chromatin loops identified from the m-bud, g-bud and m-leaf contact matrices.

Additional Table S4 Differential chromatin loops in the g-bud vs. m-bud and m-leaf vs. m-bud comparisons.

Additional Table S5 Differential chromatin contacts at 5 kb resolution in the g-bud vs. m-bud and m-leaf vs. m-bud comparisons.

Additional Table S6 DEGs identified in the g-bud vs. m-bud comparison.

Additional Table S7 Results of transcriptome comparison between g-bud and m-bud samples.

Additional Table S8 List of enriched genes annotated with GO and KEGG analyses.

Additional Table S9 DEGs overlapping with differential contact regions at 5 kb and 10 kb resolutions between g-bud and m-bud samples.

Additional Table S10 Twelve DEGs that might be involved in sex differentiation located in differential contact regions between the g-bud and m-bud samples.

Additional Table S11 List of transcriptome data for co-expression analysis.

Additional Table S12 Gene list for the MEgreen, MEdarkgreen and MElightcyan modules.

Additional Table S13 GO enrichment analysis of the co-expressed genes in the MEgreen, MEdarkgreen and MElightcyan modules.

 Click here to access/download;Table;Table1.docx

Table 1 Statistics of our *Jatropha* genome assembly

Assembly	Number	N50	N75	L50	L75	Total length (kb)
Contigs	1,265	1,029,648	362,618	86	246	378,337,367
Scaffolds	1,196	30,651,357	27,306,515	6	10	379,507,867
chromosomes	11	-	-	-	-	337,277,379
Coding genes	25,817	-	-	-	-	40,884,597

Click here to access/download;Figure;Figure1.pdf

Figure 1

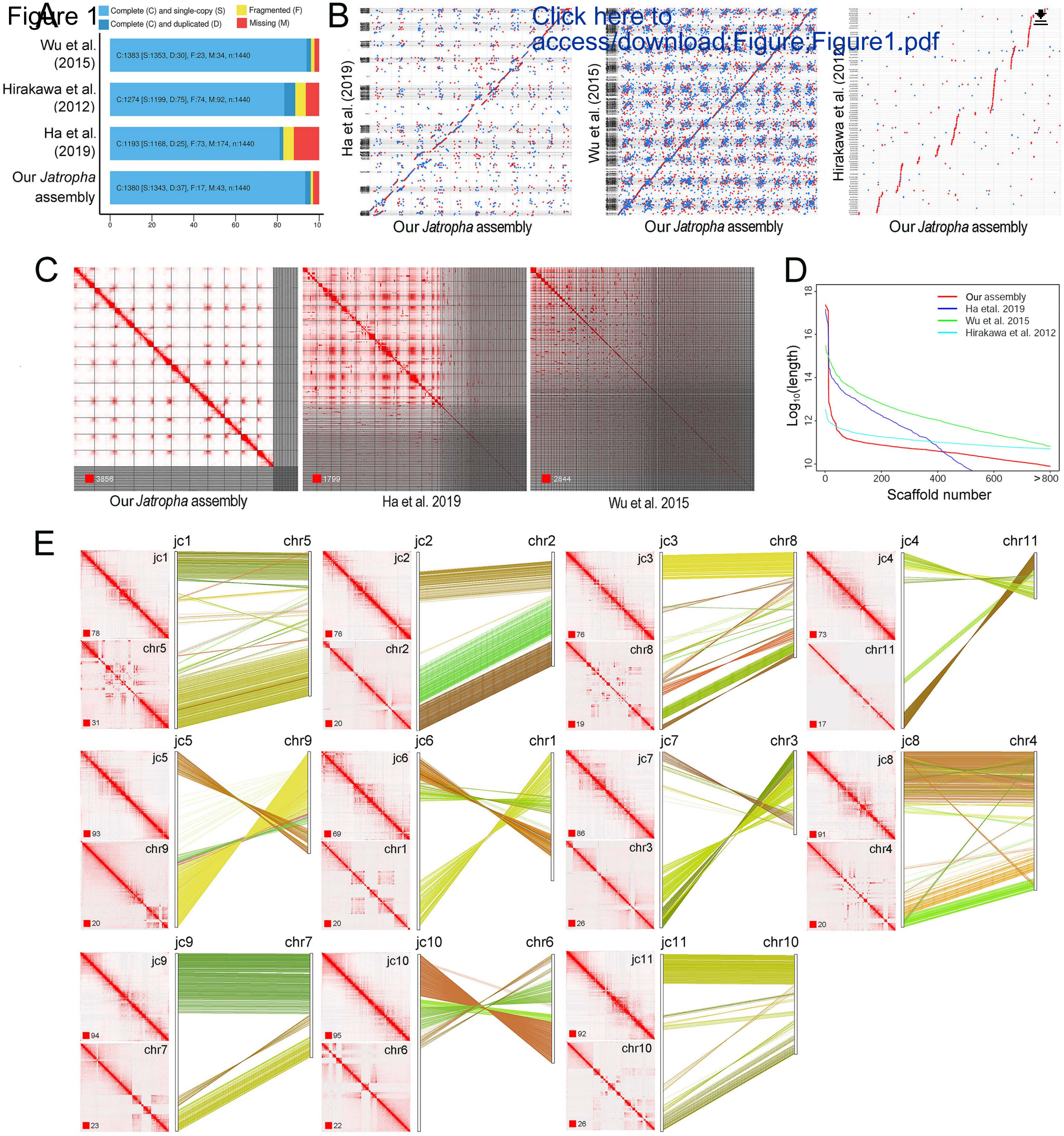

Figure 2

Click here to access/download;Figure;Figure2

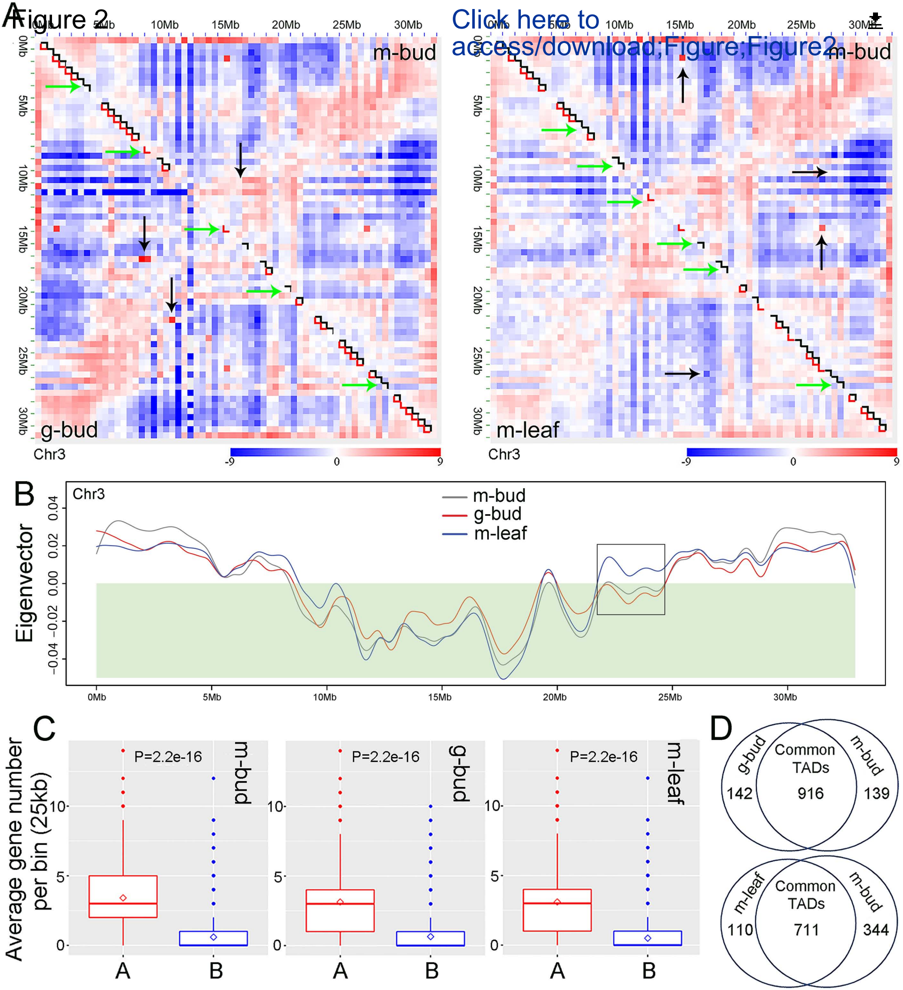

Figure 3

Click here to access/download;Figu

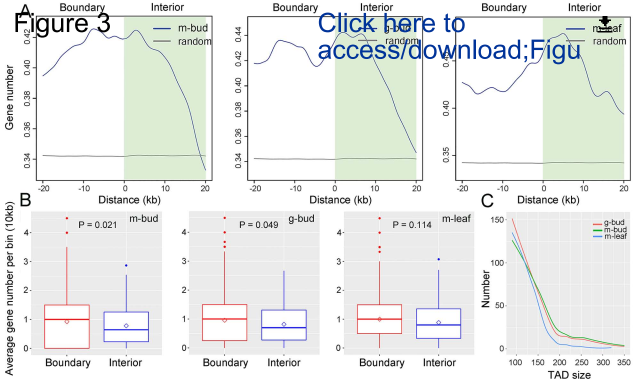

Figure 4

Click here to access/download;Figure;Figure4.

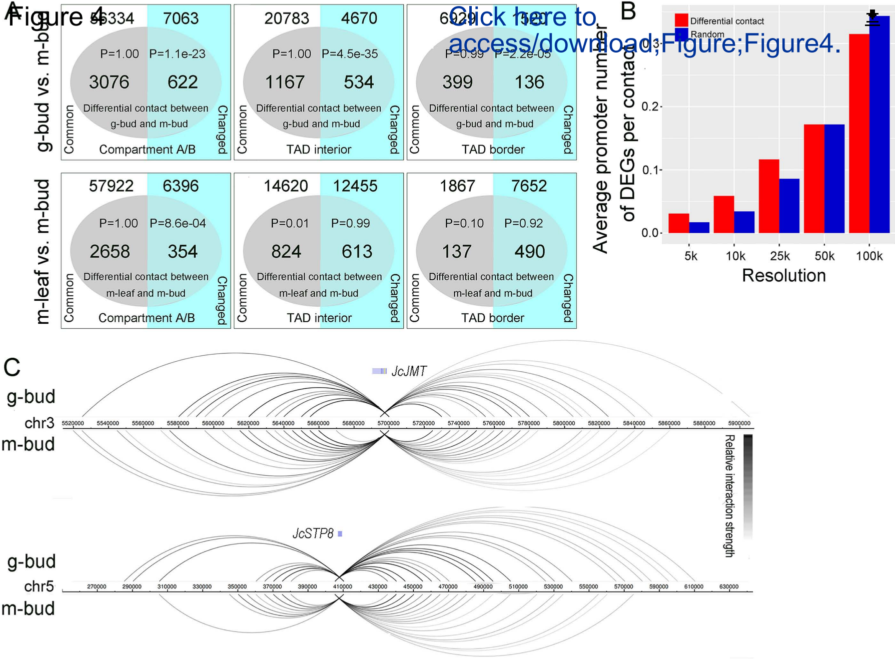

Figure 5

Click here to access/download;Figu

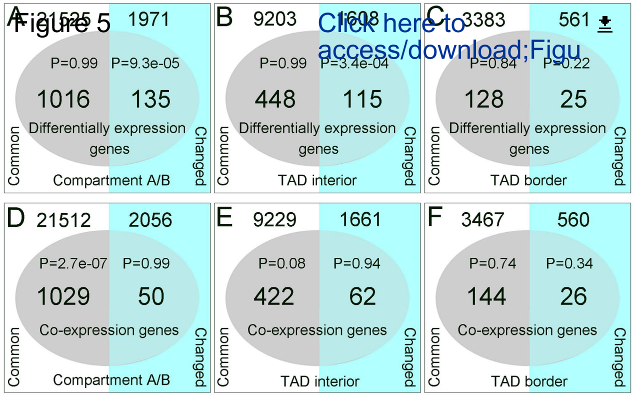

Additional Figure S1

Click here to access/download
Supplementary Material
Additional Figure S1.tif

Additional Figure S2

Click here to access/download
Supplementary Material
Additional Figure S2.tif

Additional Figure S3

Click here to access/download
Supplementary Material
Additional Figure S3.tif

Additional Figure S4

Click here to access/download
Supplementary Material
Additional Figure S4.tif

Additional Figure S5

Click here to access/download
Supplementary Material
Additional Figure S5.tif

Additional Figure S6

Click here to access/download
Supplementary Material
Additional Figure S6.tif

Additional Figure S7

Click here to access/download
Supplementary Material
Additional Figure S7.tif

Additional Figure S8

Click here to access/download
Supplementary Material
Additional Figure S8.tif

Additional Figure S9

Click here to access/download
Supplementary Material
Additional Figure S9.tif

Additional Figure S10

Click here to access/download
Supplementary Material
Additional Figure S10.tif

Additional Figure S11

Click here to access/download
Supplementary Material
Additional Figure S11.tif

Additional Figure S12

Click here to access/download

Supplementary Material

Additional Figure S12.tif

Additional Table S1

Click here to access/download
Supplementary Material
Additional Table S1.docx

Additional Table S2

Click here to access/download
Supplementary Material
Additional Table S2.xlsx

Additional Table S3

Click here to access/download
Supplementary Material
Additional Table S3.xls

Additional Table S4

Click here to access/download
Supplementary Material
Additional Table S4.xlsx

Additional Table S5

Click here to access/download
Supplementary Material
Additional Table S5.xlsx

Additional Table S6

Click here to access/download
Supplementary Material
Additional Table S6.xls

Additional Table S7

Click here to access/download
Supplementary Material
Additional Table S7.xlsx

Additional Table S8

Click here to access/download
Supplementary Material
Additional Table S8.xlsx

Additional Table S9

Click here to access/download
Supplementary Material
Additional Table S9.xlsx

Additional Table S10

Click here to access/download
Supplementary Material
Additional Table S10.xlsx

Additional Table S11

Click here to access/download
Supplementary Material
Additional Table S11.xlsx

Additional Table S12

Click here to access/download
Supplementary Material
Additional Table S12.xlsx

Additional Table S13

Click here to access/download
Supplementary Material
Additional Table S13.xlsx

 Click here to access/download;Personal Cover;Response to Reviewers_Chen_GIGA-D-19-00223.docx

GIGA-D-19-00223

Dr. Hongling Zhou

Editor, GigaScience

Dear Dr. Zhou,

Thank you for your decision letter of August 12 and for the reviewers' comments on our manuscript entitled "Chromatin architecture alterations are associated with sex differentiation in the woody plant *Jatropha curcas*, based on an improved genome assembly and comparative transcriptome analysis between monoecious and gynoecious inflorescence buds", by Chen et al. (GIGA-D-19-00223). Reviewers' comments and suggestions greatly helped us to improve the manuscript. We have addressed the reviewers' concerns in a revised version of our manuscript, which we are resubmitting for your consideration.

As suggested by the Reviewer 2, the title was revised as "*De novo* genome assembly and Hi-C analysis reveal the association between chromatin architecture alterations and sex differentiation in the woody plant *Jatropha curcas*" in the revised manuscript. All other revisions in the revised manuscript are highlighted in red text.

Our point-by-point responses to the comments of the editor and the reviewers are as follows:

Responses to the comments of the editor:

Please also meet our reproducibility guidelines, as we would ask you add identifiers (ORCID IDs for authors, RRIDs for software, NCBI taxon IDs for species, etc.) and would also recommend including protocols in protocols.io.

Response: Thanks for your suggestion. The identifiers were added in lines 21-30, 357, 367, 377-392, 418 and 432-441 in the revised manuscript.

Responses to the comments of the Reviewer 1:

1. One issue is that the FDR used is unclear. It is 0.05 in page 9 but 0.01 in page 15. From Additional Table S5 it seems like the threshold used was 0.05.

Response: Thanks for your comment. An FDR of $\leqslant$ 0.05 was used in this study. The error was corrected in line 434 on page 16 in the revised manuscript.

2. In addition, it would be useful to provide the RNAseq result of all genes so that interested parties can apply their own logFC and FDR thresholds when comparing studies.

Response: The RNA-seq result of all genes was included in the Additional Table S7 in the revised manuscript.

3. Finally a volcano plot that shows the effect of the threshold would also help visualizing the effect of choosing different thresholds.

Response: A volcano plot of transcriptome analysis was added as an Additional Figure S8.

4. Page 9: "These genes" - It is unclear if this refers to 12 or 8 genes. What are the identification criteria for the 12 and 8 genes? Do the remaining 241 genes not follow these criteria?

Response: “These genes” indicates the 12 genes, which may be involved in flower development or biosynthesis of phytohormones associated with sex differentiation in *Jatropha*. We have clarified this in lines 263-276 of the revised manuscript.

5. The version of the software used is missing from all text except the Methods section. Is this acceptable?

Response: **We** added the version information of the software in all text in the revised

manuscript.

6. Fig S6: Please make the new assembly to be collinear with Ha et al, which will make any comparisons easier. Ideally the raw files are as collinear as possible, but at least the figure should be so that all remaining inconsistencies are more likely to be true inversions.

Response: Genome-wide collinearity comparison has displayed in Figure 1B, which is generated with the entire genome sequences in the revised manuscript. The old Additional Figures S5 and S6 were merged into a new Figure 1E to show the collinear comparison of gene coding sequences of corresponding chromosomes between our assembly and the Ha et al. (2019) assembly, in which the differences between chromosomes are originated from respective genome assemblies.

Minor comments

7. Page 2, second last sentence "Among these DEGs" -> insert number of DEGs.

Response: We revised the relevant description in lines 45-49 on page 2 in the revised manuscript.

8. Page 5: "real-time (SMRT)" -> "PacBio", as in first paragraph of results

Response: We revised the "real-time (SMRT)" as "PacBio" in line 135 on page 5 in the revised manuscript.

9. Page 6: "The assembly includes" -> "The assembly is comprised of", or specify what else it includes.

Response: We revised the "The assembly includes" as "The assembly is comprised of" in line 149 on page 6 in the revised manuscript.

10. Page 6: delete ", in which most of the errors were from substitutions"

Response: We revised the relevant description in lines 166-168 on pages 6 and 7 in the revised manuscript.

11. Page 6: The BUSCO result text is unnecessarily long. It is sufficient to say it is best that other assemblies, and use the [] format used in Fig 6 to describe the result.

Response: We revised the description of BUSCO result in lines 172-174 on page 7 in the revised manuscript.

12. Page 8, first paragraph, second last sentence: "transcriptions" -> transcription

Response: We revised "transcriptions" as "transcription" in line 212 on page 8 in the revised manuscript.

13. Page 8: "((Additional Table S3)" -> "(Additional Table S3)"

Response: We removed the extra left bracket in line 221 on page 8 in the revised manuscript.

14. Page 10: remove the non-capitalised "is a sugar transport protein that" and "is a jasmonic acid carboxyle methyltransferase that" as the information just repeats the gene name.

Response: We revised the sentence in lines 282-284 on page 11 in the revised manuscript.

15. Page 13: "used to the train" -> "used to train"

Response: we revised "used to the train" as "used to train" in line 386 on page 14 in the revised manuscript.

16. Fig S3: "Scoffold" -> "Scaffold"

Response: We revised "Scoffold" as "Scaffold" in the new Figure 1D (old Additional Figure S3) in the revised manuscript.

Responses to the comments of the Reviewer 2:

1. Title is too long, May I suggest a title as" Chromatin architecture alterations are associated with sex differentiation in the woody plant Jatropha curcas".

<u>Response</u>: Thanks for your suggestion. We revised the title as "*De novo* genome assembly and Hi-C analysis reveal the association between chromatin architecture alterations and sex differentiation in the woody plant *Jatropha curcas*".

2. Introduction: Authors should include some more literatures on effect of exogenous hormones to Jatropha flowerin. Several works have been reported on a possible หำป sex determination genes.

<u>Response</u>: More literatures were included in lines 94-101 on page 4 in the introduction section of the revised manuscript.

3. Discussions: Page 12: There are a couple of literatures of effect on cytokinin to Jatropha flowering genes expression. Authors need to include these literatures if they want to speculate the important of cytokinin biosynthesis genes.

<u>Response</u>: More literatures were included in lines 343-344 on page 13 in the discussion section of the revised manuscript.

Responses to the comments of the Reviewer 3:

1. The current DEGs part is very general. Even though you have a previous publication on it, new DEGs result should be presented in detail other than simply some values of DEGs. In my view, the DEGs should be summarized on their functional categories (such as GO, Kegg, flower pathways, sexual determination pathways). Then, the readers could have chance to build a comprehensive picture of gene expression related to the biological process, and to the effects of chromatin organization on gene expression. If you include more gene expression (see my next comment), please provide in-depth description on gene expression and co-expression. That is not difficult.

<u>Response</u>: Thanks for your suggestion. We added GO and KEGG enrichment analysis of DEGs (Additional Figure S9, S10, and Additional Table S8) and co-expression genes (Additional Figure S12 and Additional Table S13) in lines 249-252 on pages 9 and 10, and lines 287-293 on page 11, respectively, in the result section of the revised manuscript.

2. Chromatin architecture alternation of sex differentiation is the core point of this article. Comparison between chromatin contact and gene expression units makes the most key part of the analyses present in the study. While, there are still lacking some interesting staff, such as comparison between gene co-expression profile and chromatin organization. The published gene expression reports (including the No. 64 cited in the reference list, and some others listed in the following lines) provide enough data for a gene co-expression study. Please collect more RNA-seq data, enrich the gene expression part, construct gene co-expression profile, compare the gene co-expression and chromatin contact, make in-depth investigation and description, and raise potential hypothesis. We would like to know if such genes contacted in chromatin level go together in gene expression for example in "m-bud", "gbud" and "m-leaf". Or which set of genes do that, which do not? This means the author make need to provide a gene co-expression study, and then for this kind of comparsion. Such kind of analyse is a rountine way to examine the connection between gene expression and chromatin organization. Please see the articles listed below.

<u>Response</u>: We performed co-expression analysis and found that the co-expressed genes related to the "Reproductive process" are irrelevant to chromatin structure changes, however, DEGs preferentially occur in the changed regions of compartment A/B and TADs (Figure 5), implying that chromatin organization is associated with gene transcription. The relevant description is added in lines 293-299 on page 11 in the result section of the revised manuscript.

3. Page 5, "suggesting that chromatin organization may be different between grasses and woody plants". If you only have cases from one grass (here rice) and one woody

plant (Jatropha), keep conservative and give up this tandency, as few people will agree with you.

Response: We revised the description in lines 210-217 on page 8 in the revised manuscript.

4. I see some results not well described in the results part but in the discussion part, like all you discussed in Page 11, especially the genes IDs, I did read them in results. Please enrich the results, do not be so general. You may put them into supplementary file, if you are worrying about the space.

Response: An additional Table S10 was included and the relevant description was added in lines 263-275 on page 10 in the revised manuscript.

5. Line number is lacking. And it is very hard for reviewers to provide their comments.

Response: Sorry for this mistake. Line number was added in the revised manuscript.

6. Please replace "new Jatropha assembly" in different Figures or Additional Figures with "our assembly" or "the present assembly".

Response: All "new *Jatropha* assembly" was replaced with "our *Jatropha* assembly" in all figures in the revised manuscript.

7. Additional Figures S3. I may want to see different assemblies were contrasted in one panel by overlying different items, but not in separated ones. And also transformation could applied to X or Y variables, so as to get good visitation.

Response: The old additional Figure S3 was replaced with a new Figure 1D in the revised manuscript.

8. Why the assembly from Kancharla et al. 2019 was never compared in analyses presented in Figure 2, Additional Figure S3 and other Figures? But in that in Additional Table S1?

Response: The quality of the assembly from Kancharla et al. 2019 is poor as shown

in Additional Table S1. In addition, the assembly from Kancharla et al. 2019 hasn't been used widely in *Jatropha* research as that of assemblies from Hirakawa et al. 2012 and Wu et al. 2015. Therefore, we didn't perform further comparative analysis.

9. Additional Figures S5 is a partial one. Please provide an intact one.

Response: The old Additional Figures S5 was replaced with a new Figure 1E, showing chromosomal differences between our *Jatropha* assembly and the assembly from Ha et al. 2019 in the revised manuscript.

10. Additional Figures S7. Please provide the contrast in one panel, not two or more.

Response: The old Additional Figure S7 was replaced with a new Figure 3C in the revised manuscript.

11. Figure 5, hard to see yellow box and cyan square, only black square could I see. Please refine this figure.

Response: The old Figure 5 was removed, and the contents were incorporated into the new Figures 2 and 3 in the revised manuscript.

12. Figure 6, hard to see red square, only dark squares I see. Please refine this figure.

Response: The old Figure 6 was removed, and the contents were incorporated into the new Figure 2 in the revised manuscript.

We thank the reviewers for the kind help.

Yours sincerely,

Long-Jian Niu, Ph.D., Southern University of Science and Technology, China

Chunhui Hou, Ph.D., Southern University of Science and Technology, China

Zeng-Fu Xu, Ph.D., Xishuangbanna Tropical Botanical Garden, China
