# Supplementary material for: De novo genome assembly and Hi-C analysis reveal an association between chromatin architecture alterations and sex differentiation in the woody plant Jatropha curcas
Source: Gigascience. 2020 Feb 12;9(2):giaa009. doi: 10.1093/gigascience/giaa009 (PMC7014976; doi:10.1093/gigascience/giaa009)
Supplement: giaa009_Supplemental_Figures_and_Tables [file giaa009_supplemental_figures_and_tables.zip › Additional Table S1.docx]

Additional Table S1 Statistics of our assembly and the published *Jatropha* assemblies

| Assembly | Hirakawa *et al*. 2012 | Wu *et al*. 2015 | Kancharla *et al*. 2019 | Ha *et al*. 2019 | our assembly in this study |
| --- | --- | --- | --- | --- | --- |
| All Scaffolds | 39,277 | 6,024 | 2959 | 812 | 1,196 |
| Scaffolds (>50Kb) | 602 | 795 |  | 397 | 231 |
| Total length | 297,661,187 | 318,527,106 | 265,767,376 | 339,501,388 | 379,059,919 |
| Chromosome number | - | - | - | 11 | 11 |
| Chromosome length | - | - | - | 204,206,989 | 337,277,379 |
| GC content (%) | 33.68 | 33.29 | 33.40 | 34.96 | 35.23 |
| N50 | 18,099 | 755,248 | 169,453 | 15,395,338 | 30,651,357 |
| N75 | 9,278 | 242,702 |  | 708,318 | 27,306,515 |
| L50 | 3,845 | 94 |  | 9 | 6 |
| L75 | 9,148 | 286 |  | 54 | 10 |
| N's per 100 kb | 406.53 | 17,652.20 | - | 239.16 | 116.01 |
