# Supplementary figures and images for: De novo genome assembly and Hi-C analysis reveal an association between chromatin architecture alterations and sex differentiation in the woody plant Jatropha curcas
Source: Gigascience. 2020 Feb 12;9(2):giaa009. doi: 10.1093/gigascience/giaa009 (PMC7014976; doi:10.1093/gigascience/giaa009)

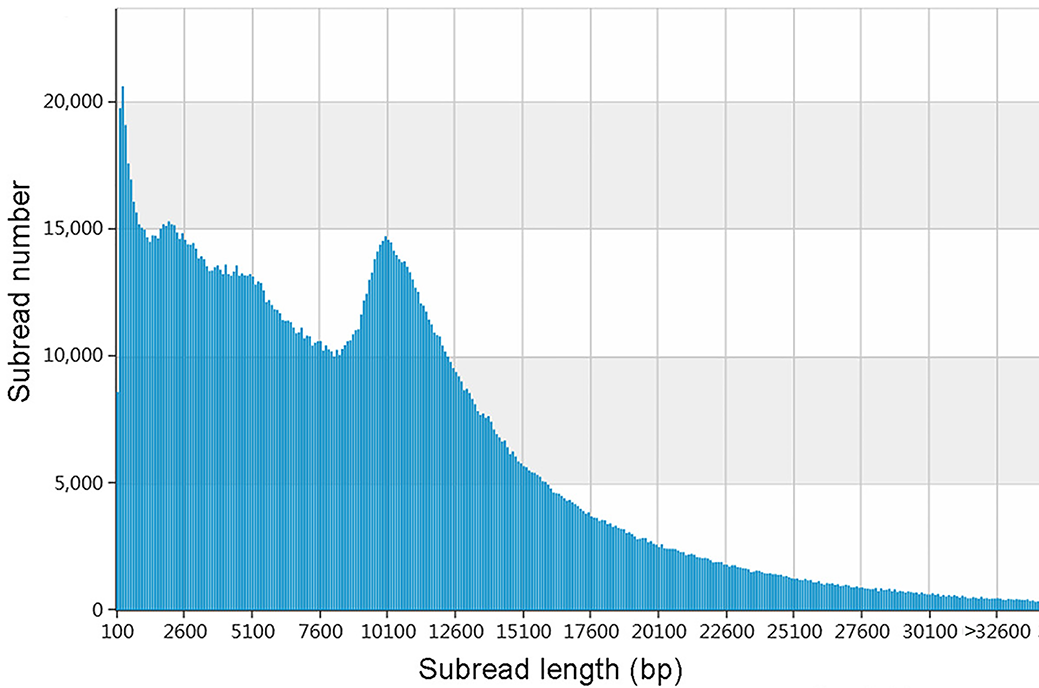

Supplement: giaa009_Supplemental_Figures_and_Tables [file giaa009_supplemental_figures_and_tables.zip › Additional Figure S1.tif]

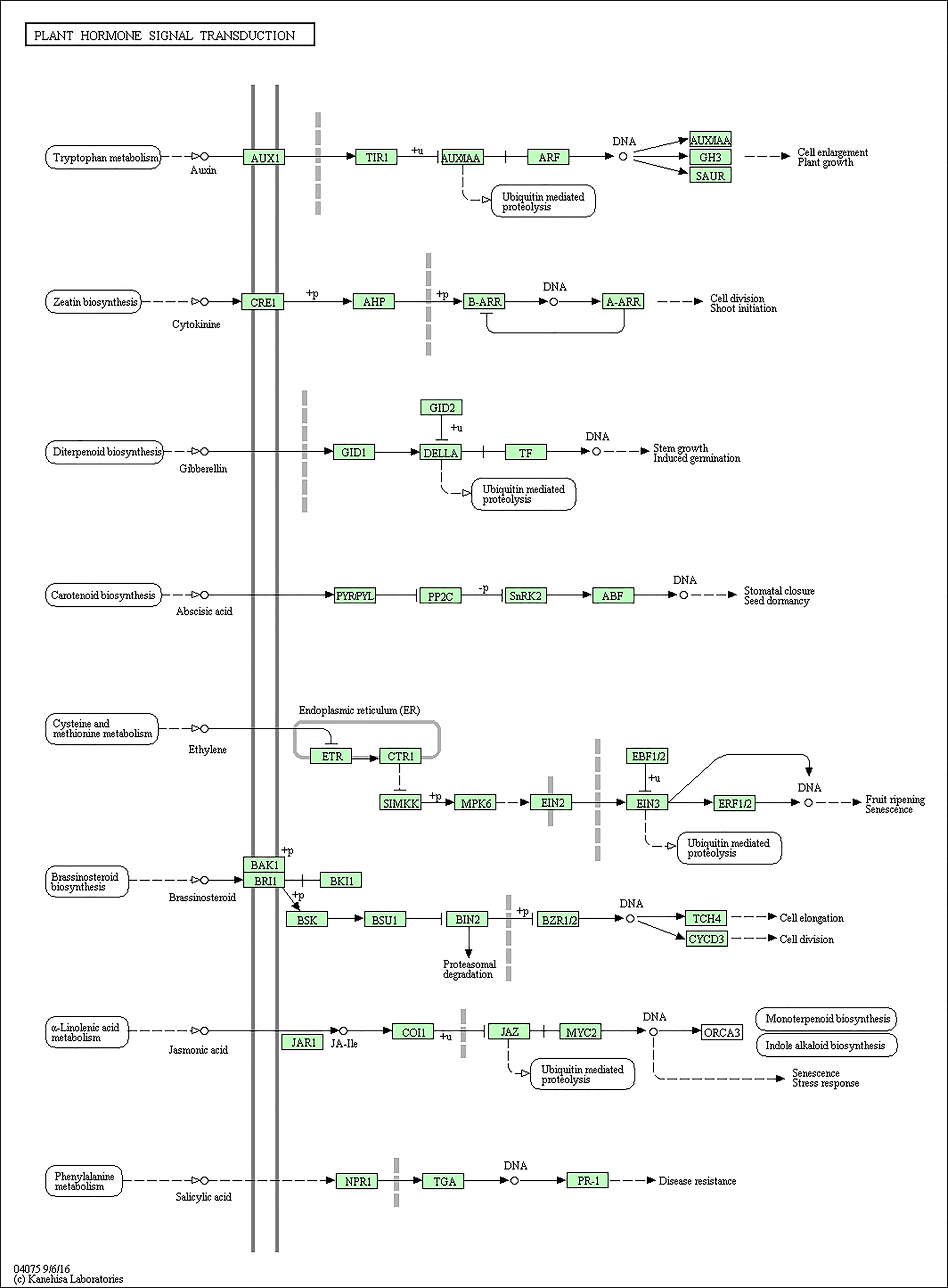

Supplement: giaa009_Supplemental_Figures_and_Tables [file giaa009_supplemental_figures_and_tables.zip › Additional Figure S10.tif]

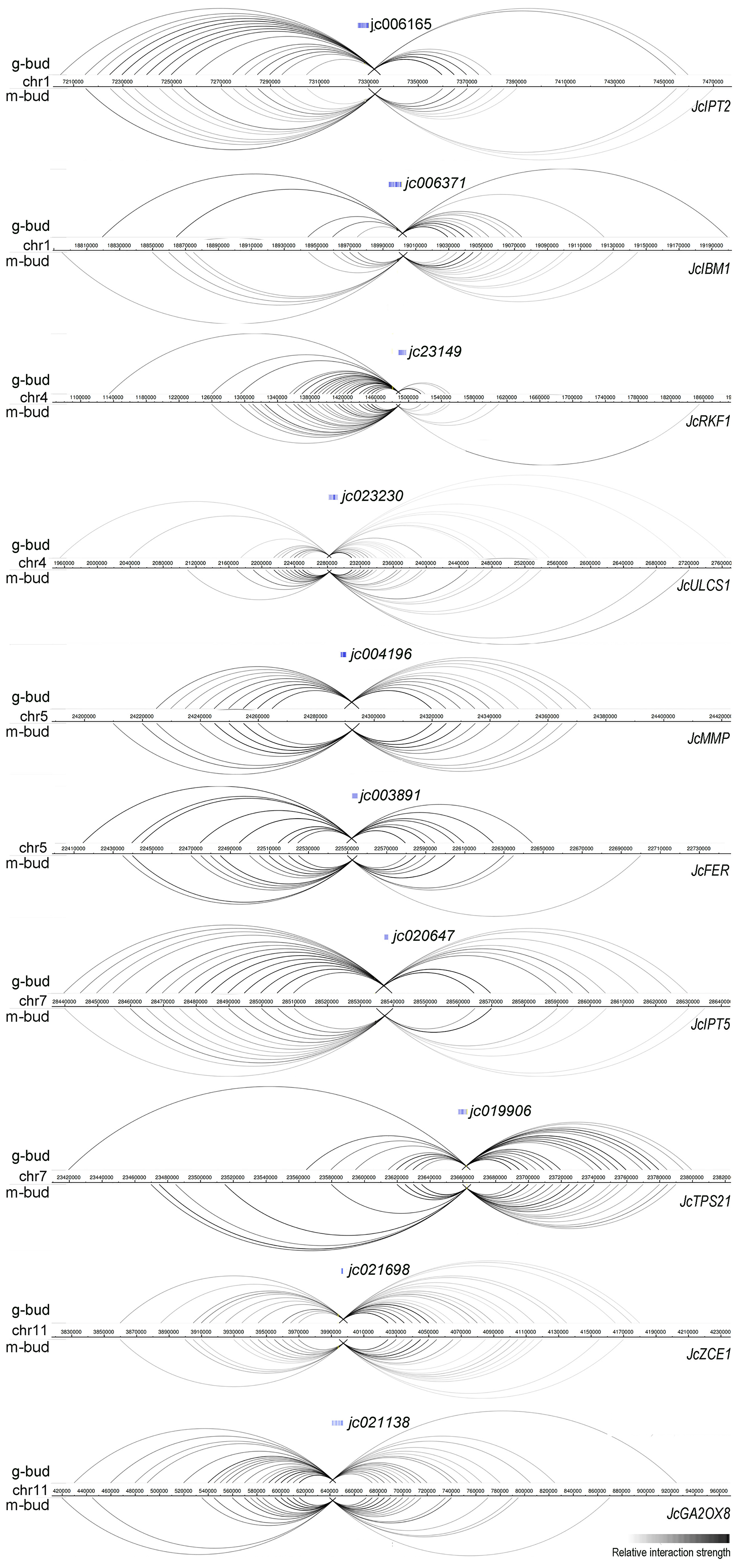

Supplement: giaa009_Supplemental_Figures_and_Tables [file giaa009_supplemental_figures_and_tables.zip › Additional Figure S11.tif]

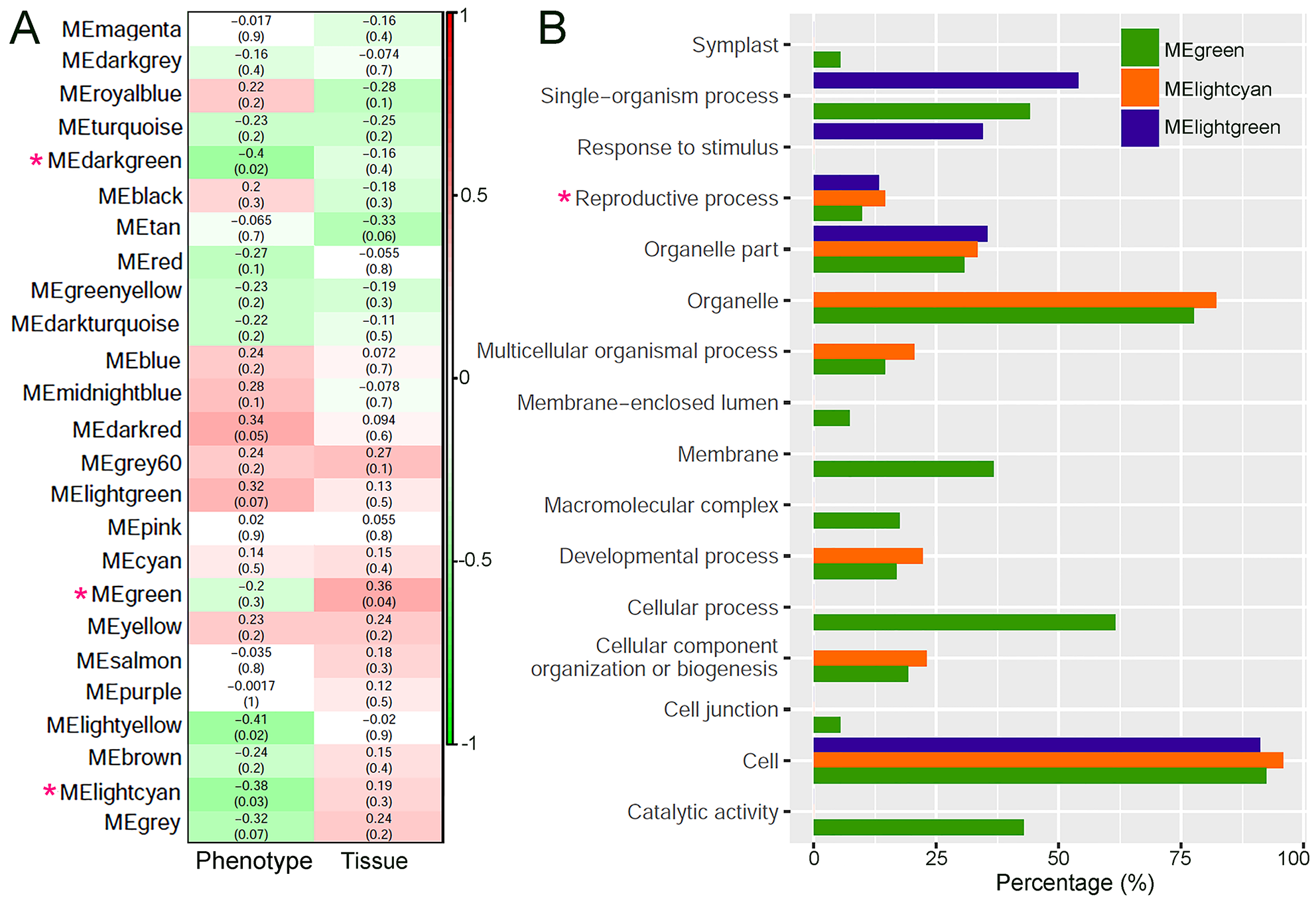

Supplement: giaa009_Supplemental_Figures_and_Tables [file giaa009_supplemental_figures_and_tables.zip › Additional Figure S12.tif]

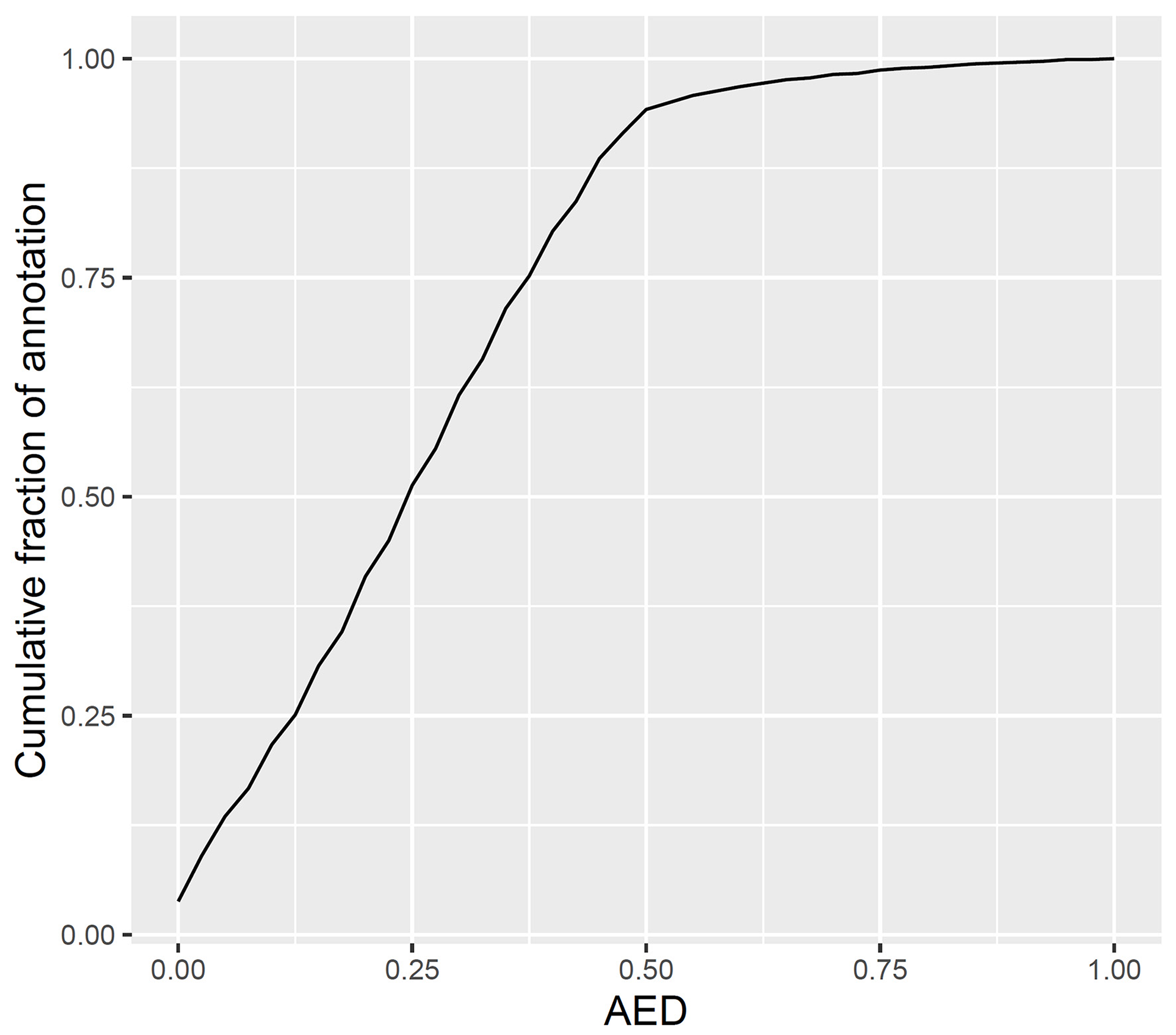

Supplement: giaa009_Supplemental_Figures_and_Tables [file giaa009_supplemental_figures_and_tables.zip › Additional Figure S2.tif]

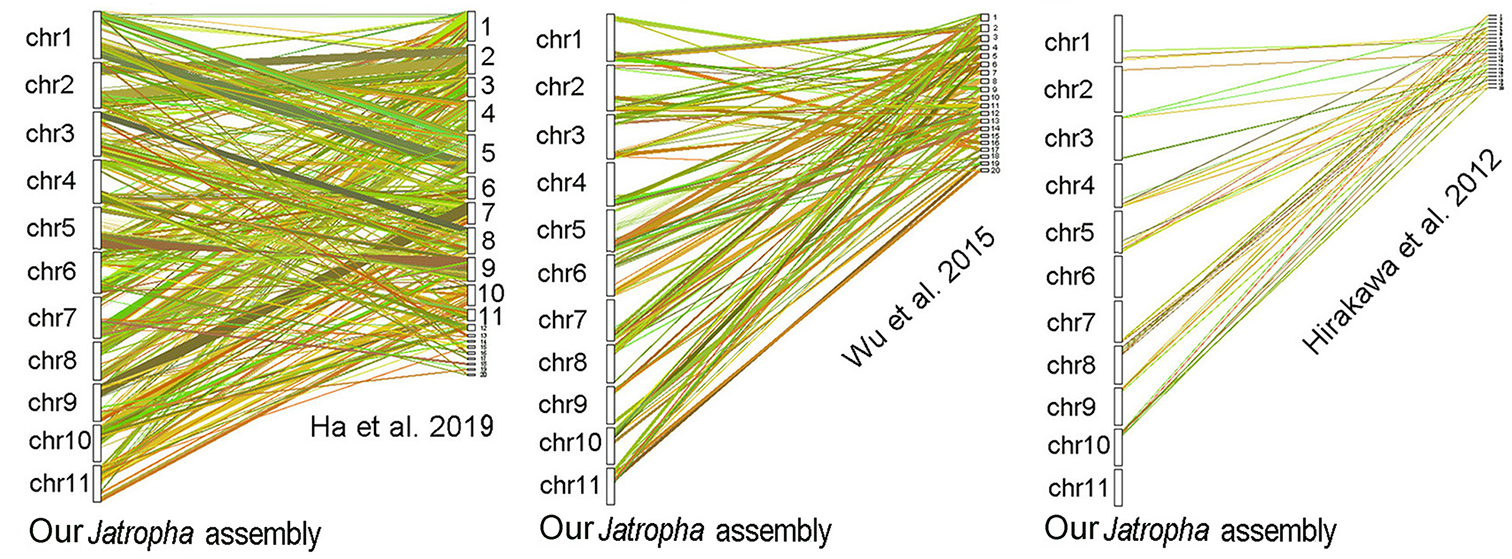

Supplement: giaa009_Supplemental_Figures_and_Tables [file giaa009_supplemental_figures_and_tables.zip › Additional Figure S3.tif]

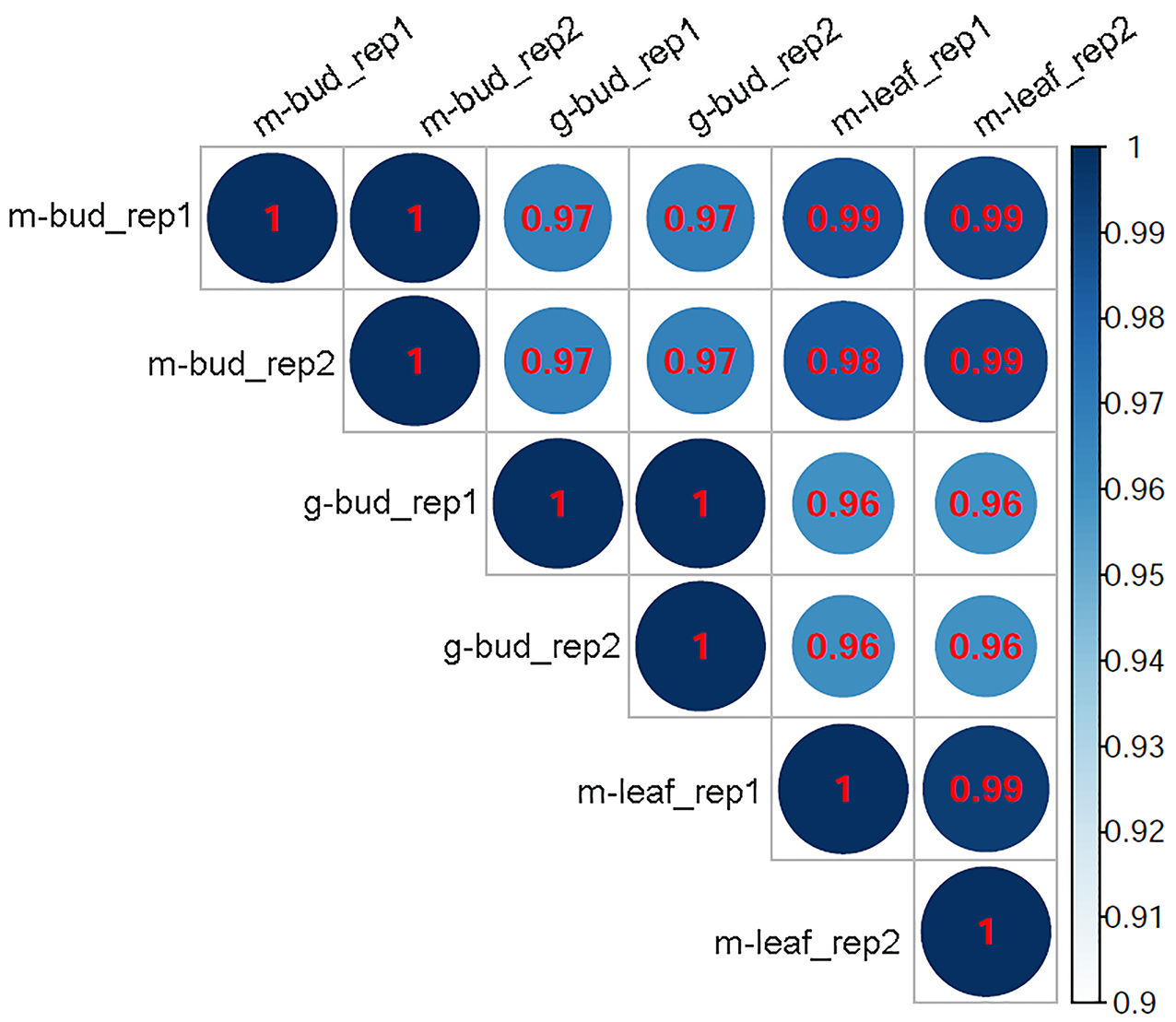

Supplement: giaa009_Supplemental_Figures_and_Tables [file giaa009_supplemental_figures_and_tables.zip › Additional Figure S4.tif]

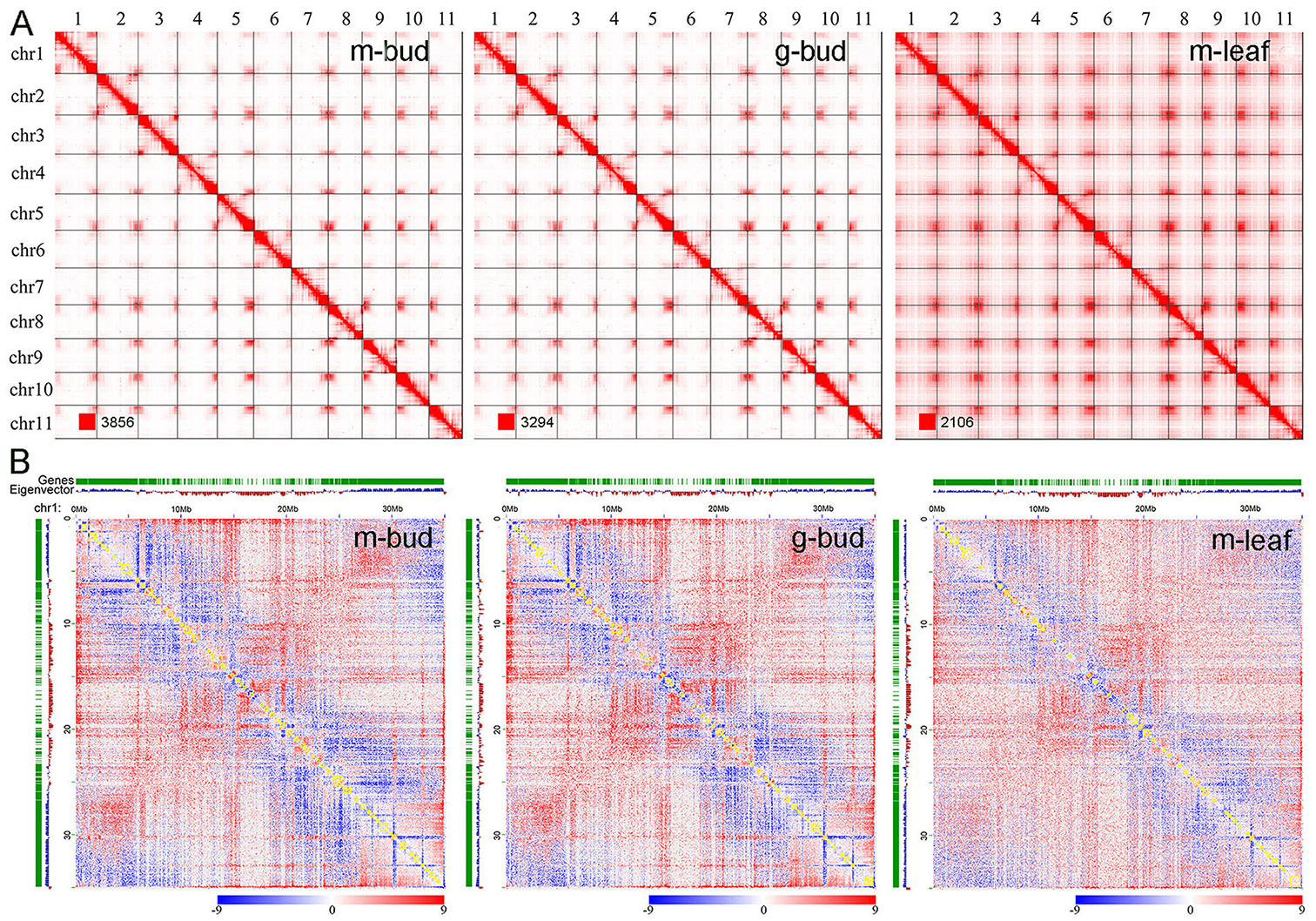

Supplement: giaa009_Supplemental_Figures_and_Tables [file giaa009_supplemental_figures_and_tables.zip › Additional Figure S5.tif]

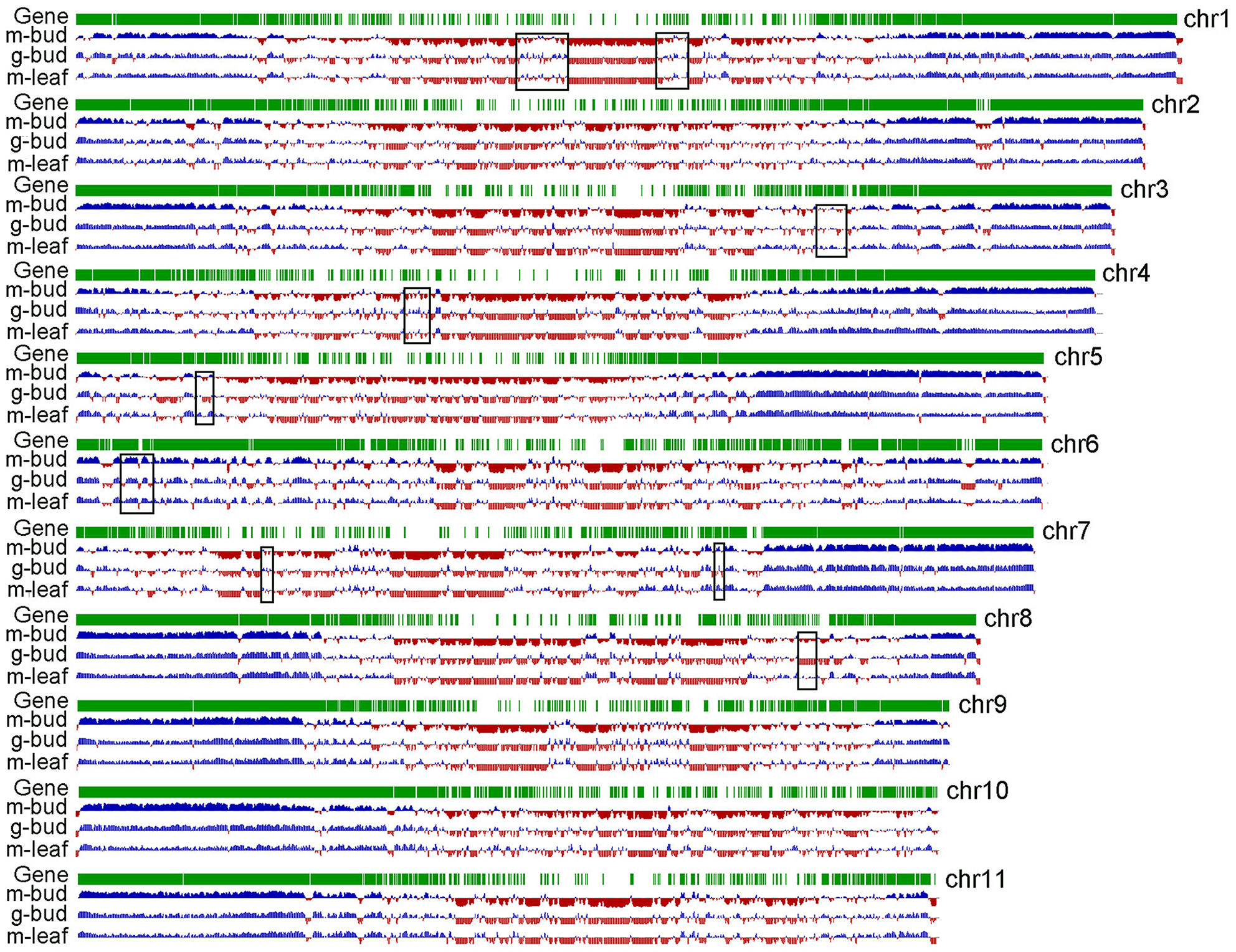

Supplement: giaa009_Supplemental_Figures_and_Tables [file giaa009_supplemental_figures_and_tables.zip › Additional Figure S6.tif]

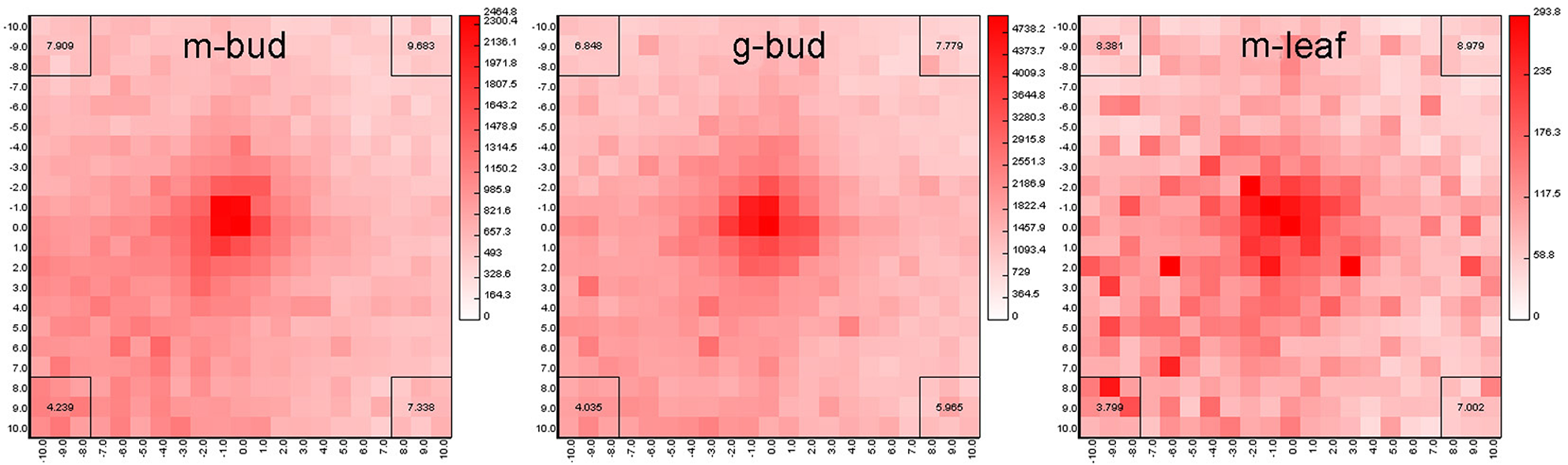

Supplement: giaa009_Supplemental_Figures_and_Tables [file giaa009_supplemental_figures_and_tables.zip › Additional Figure S7.tif]

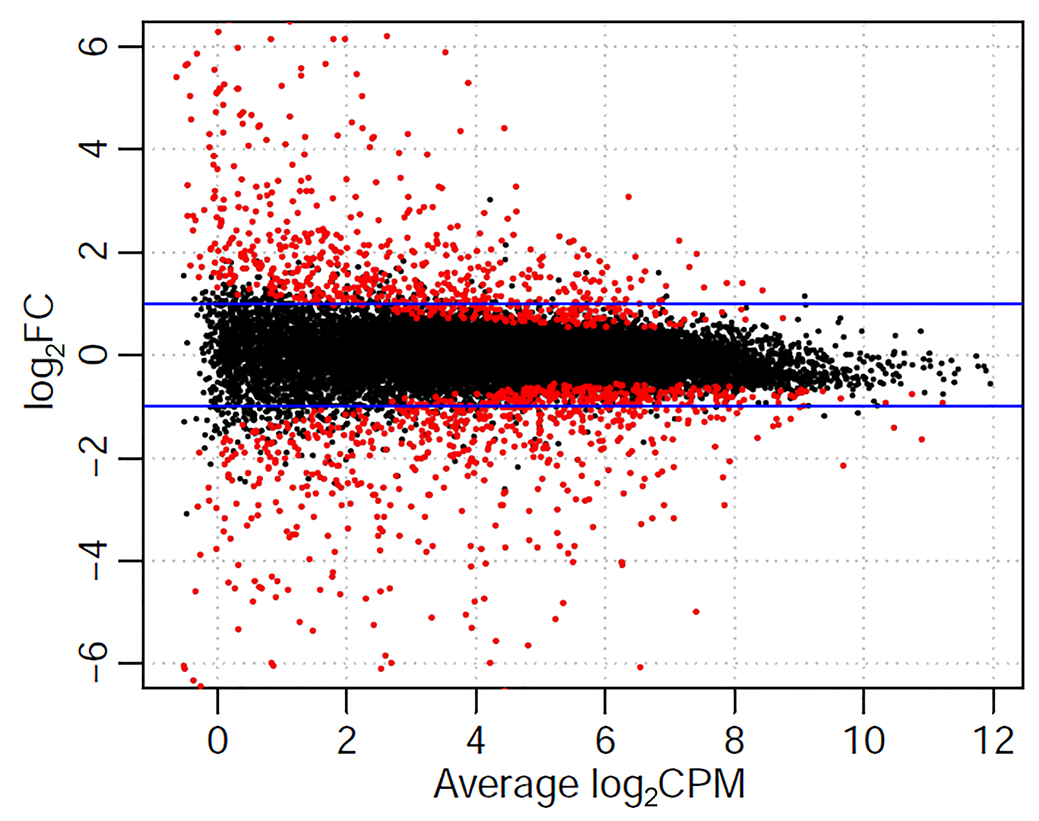

Supplement: giaa009_Supplemental_Figures_and_Tables [file giaa009_supplemental_figures_and_tables.zip › Additional Figure S8.tif]

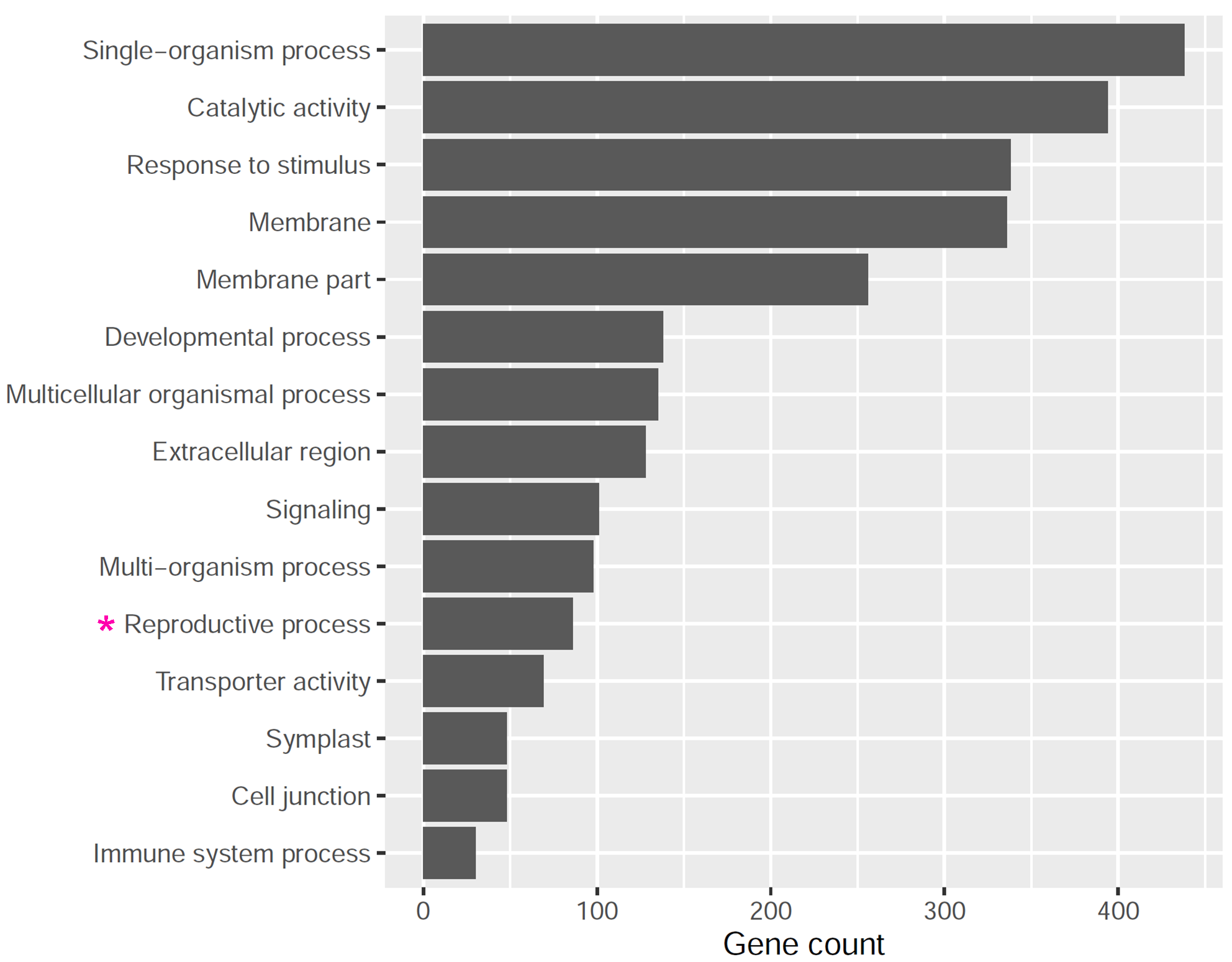

Supplement: giaa009_Supplemental_Figures_and_Tables [file giaa009_supplemental_figures_and_tables.zip › Additional Figure S9.tif]
