# Supplementary material for: De novo genome assembly and Hi-C analysis reveal an association between chromatin architecture alterations and sex differentiation in the woody plant Jatropha curcas
Source: Gigascience. 2020 Feb 12;9(2):giaa009. doi: 10.1093/gigascience/giaa009 (PMC7014976; doi:10.1093/gigascience/giaa009)
Supplement: giaa009_Response_to_Reviewer_Comments_Original_Submission [file giaa009_response_to_reviewer_comments_original_submission.pdf]

Author's Response To Reviewer Comments

Close

Our point-by-point responses to the comments of the editor and the reviewers are as follows:

Responses to the comments of the editor:
Please also meet our reproducibility guidelines, as we would ask you add identifiers (ORCID IDs for authors, RRIDs for software, NCBI taxon IDs for species, etc.) and would also recommend including protocols in protocols.io.
Response: Thanks for your suggestion. The identifiers were added in lines 21-30, 357, 367, 377-392, 418 and 432-441 in the revised manuscript.

Responses to the comments of the Reviewer 1:

1. One issue is that the FDR used is unclear. It is 0.05 in page 9 but 0.01 in page 15. From Additional Table S5 it seems like the threshold used was 0.05.
Response: Thanks for your comment. An FDR of ≤ 0.05 was used in this study. The error was corrected in line 434 on page 16 in the revised manuscript.

2. In addition, it would be useful to provide the RNAseq result of all genes so that interested parties can apply their own logFC and FDR thresholds when comparing studies.
Response: The RNA-seq result of all genes was included in the Additional Table S7 in the revised manuscript.

3. Finally a volcano plot that shows the effect of the threshold would also help visualizing the effect of choosing different thresholds.
Response: A volcano plot of transcriptome analysis was added as an Additional Figure S8.

4. Page 9: "These genes" - It is unclear if this refers to 12 or 8 genes. What are the identification criteria for the 12 and 8 genes? Do the remaining 241 genes not follow these criteria?
Response: “These genes” indicates the 12 genes, which may be involved in flower development or biosynthesis of phytohormones associated with sex differentiation in Jatropha. We have clarified this in lines 263-276 of the revised manuscript.

5. The version of the software used is missing from all text except the Methods section. Is this acceptable?
Response: We added the version information of the software in all text in the revised manuscript.

6. Fig S6: Please make the new assembly to be collinear with Ha et al, which will make any comparisons easier. Ideally the raw files are as collinear as possible, but at least the figure should be so that all remaining inconsistencies are more likely to be true inversions.
Response: Genome-wide collinearity comparison has displayed in Figure 1B, which is generated with the entire genome sequences in the revised manuscript. The old Additional Figures S5 and S6 were merged into a new Figure 1E to show the collinear comparison of gene coding sequences of corresponding chromosomes between our assembly and the Ha et al. (2019) assembly, in which the differences between chromosomes are originated from respective genome assemblies.

Minor comments
7. Page 2, second last sentence "Among these DEGs" -> insert number of DEGs.
Response: We revised the relevant description in lines 45-49 on page 2 in the revised manuscript.

8. Page 5: "real-time (SMRT)" -> "PacBio", as in first paragraph of results
Response: We revised the "real-time (SMRT)" as "PacBio" in line 135 on page 5 in the revised manuscript.

9. Page 6: "The assembly includes" -> "The assembly is comprised of", or specify what else it includes.

Response: We revised the "The assembly includes" as "The assembly is comprised of" in line 149 on page 6 in the revised manuscript.

10. Page 6: delete ", in which most of the errors were from substitutions"
Response: We revised the relevant description in lines 166-168 on pages 6 and 7 in the revised manuscript.

11. Page 6: The BUSCO result text is unnecessarily long. It is sufficient to say it is best that other assemblies, and use the [] format used in Fig 6 to describe the result.
Response: We revised the description of BUSCO result in lines 172-174 on page 7 in the revised manuscript.

12. Page 8, first paragraph, second last sentence: "transcriptions" -> transcription
Response: We revised "transcriptions" as "transcription" in line 212 on page 8 in the revised manuscript.

13. Page 8: "((Additional Table S3)" -> "(Additional Table S3)"
Response: We removed the extra left bracket in line 221 on page 8 in the revised manuscript.

14. Page 10: remove the non-capitalised "is a sugar transport protein that" and "is a jasmonic acid carboxyle methyltransferase that" as the information just repeats the gene name.
Response: We revised the sentence in lines 282-284 on page 11 in the revised manuscript.

15. Page 13: "used to the train" -> "used to train"
Response: we revised "used to the train" as "used to train" in line 386 on page 14 in the revised manuscript.

16. Fig S3: "Scoffold" -> "Scaffold"
Response: We revised "Scoffold" as "Scaffold" in the new Figure 1D (old Additional Figure S3) in the revised manuscript.

Responses to the comments of the Reviewer 2:

1. Title is too long, May I suggest a title as" Chromatin architecture alterations are associated with sex differentiation in the woody plant Jatropha curcas".
Response: Thanks for your suggestion. We revised the title as "De novo genome assembly and Hi-C analysis reveal the association between chromatin architecture alterations and sex differentiation in the woody plant Jatropha curcas".

2. Introduction: Authors should include some more literatures on effect of exogenous hormones to Jatropha flowerin. Several works have been reported on a possible หำป sex determination genes.
Response: More literatures were included in lines 94-101 on page 4 in the introduction section of the revised manuscript.

3. Discussions: Page 12: There are a couple of literatures of effect on cytokinin to Jatropha flowering genes expression. Authors need to include these literatures if they want to speculate the important of cytokinin biosynthesis genes.
Response: More literatures were included in lines 343-344 on page 13 in the discussion section of the revised manuscript.

Responses to the comments of the Reviewer 3:

1. The current DEGs part is very general. Even though you have a previous publication on it, new DEGs result should be presented in detail other than simply some values of DEGs. In my view, the DEGs should be summarized on their functional categories (such as GO, Kegg, flower pathways, sexual determination pathways). Then, the readers could have chance to build a comprehensive picture of gene expression related to the biological process, and to the effects of chromatin organization on gene expression. If you include more gene expression (see my next comment), please provide in-depth description on gene expression and co-expression. That is not difficult.
Response: Thanks for your suggestion. We added GO and KEGG enrichment analysis of DEGs (Additional Figure S9, S10, and Additional Table S8) and co-expression genes (Additional Figure S12 and Additional Table S13) in lines 249-252 on pages 9 and 10, and lines 287-293 on page 11, respectively, in the result

section of the revised manuscript.

2. Chromatin architecture alternation of sex differentiation is the core point of this article. Comparison between chromatin contact and gene expression units makes the most key part of the analyses present in the study. While, there are still lacking some interesting staff, such as comparison between gene co-expression profile and chromatin organization. The published gene expression reports (including the No. 64 cited in the reference list, and some others listed in the following lines) provide enough data for a gene co-expression study. Please collect more RNA-seq data, enrich the gene expression part, construct gene co-expression profile, compare the gene co-expression and chromatin contact, make in-depth investigation and description, and raise potential hypothesis. We would like to know if such genes contacted in chromatin level go together in gene expression for example in "m-bud", "gbud" and "m-leaf". Or which set of genes do that, which do not? This means the author make need to provide a gene co-expression study, and then for this kind of comparsion. Such kind of analyse is a rountine way to examine the connection between gene expression and chromatin organization. Please see the articles listed below.
Response: We performed co-expression analysis and found that the co-expressed genes related to the "Reproductive process" are irrelevant to chromatin structure changes, however, DEGs preferentially occur in the changed regions of compartment A/B and TADs (Figure 5), implying that chromatin organization is associated with gene transcription. The relevant description is added in lines 293-299 on page 11 in the result section of the revised manuscript.

3. Page 5, "suggesting that chromatin organization may be different between grasses and woody plants". If you only have cases from one grass (here rice) and one woody plant (Jatropha), keep conservative and give up this tandency, as few people will agree with you.
Response: We revised the description in lines 210-217 on page 8 in the revised manuscript.

4. I see some results not well described in the results part but in the discussion part, like all you discussed in Page 11, especially the genes IDs, I did read them in results. Please enrich the results, do not be so general. You may put them into supplementary file, if you are worrying about the space.
Response: An additional Table S10 was included and the relevant description was added in lines 263-275 on page 10 in the revised manuscript.

5. Line number is lacking. And it is very hard for reviewers to provide their comments.
Response: Sorry for this mistake. Line number was added in the revised manuscript.

6. Please replace "new Jatropha assembly" in different Figures or Additional Figures with "our assembly" or "the present assembly".
Response: All "new Jatropha assembly" was replaced with "our Jatropha assembly" in all figures in the revised manuscript.

7. Additional Figures S3. I may want to see different assemblies were contrasted in one panel by overlying different items, but not in separated ones. And also transformation could applied to X or Y variables, so as to get good visitation.
Response: The old additional Figure S3 was replaced with a new Figure 1D in the revised manuscript.

8. Why the assembly from Kancharla et al. 2019 was never compared in analyses presented in Figure 2, Additional Figure S3 and other Figures? But in that in Additional Table S1?
Response: The quality of the assembly from Kancharla et al. 2019 is poor as shown in Additional Table S1. In addition, the assembly from Kancharla et al. 2019 hasn't been used widely in Jatropha research as that of assemblies from Hirakawa et al. 2012 and Wu et al. 2015. Therefore, we didn't perform further comparative analysis.

9. Additional Figures S5 is a partial one. Please provide an intact one.
Response: The old Additional Figures S5 was replaced with a new Figure 1E, showing chromosomal differences between our Jatropha assembly and the assembly from Ha et al. 2019 in the revised manuscript.

10. Additional Figures S7. Please provide the contrast in one panel, not two or more.
Response: The old Additional Figure S7 was replaced with a new Figure 3C in the revised manuscript.

11. Figure 5, hard to see yellow box and cyan square, only black square could I see. Please refine this figure.

Response: The old Figure 5 was removed, and the contents were incorporated into the new Figures 2 and 3 in the revised manuscript.

12. Figure 6, hard to see red square, only dark squares I see. Please refine this figure.
Response: The old Figure 6 was removed, and the contents were incorporated into the new Figure 2 in the revised manuscript.

We thank the reviewers for the kind help.

Close
