# Supplementary material for: De novo genome assembly and Hi-C analysis reveal an association between chromatin architecture alterations and sex differentiation in the woody plant Jatropha curcas
Source: Gigascience. 2020 Feb 12;9(2):giaa009. doi: 10.1093/gigascience/giaa009 (PMC7014976; doi:10.1093/gigascience/giaa009)
Supplement: giaa009_Reviewer_1_Report_Original_Submission — Paris Veltsos -- 7/16/2019 Reviewed [file giaa009_reviewer_1_report_original_submission.pdf]

Reviewer Report

Title: De novo genome assembly and Hi-C analysis reveal the association between chromatin architecture alterations and sex differentiation in the woody plant Jatropha curcas

Version: Original Submission **Date:** 7/16/2019

Reviewer name: Paris Veltsos

Reviewer Comments to Author:

I enjoyed reading the manuscript by Chen et al which describes chromatin architecture alterations on DNA from inflorescence tissue, associated with sex determination in Jatropha curcas. The text is easy to follow and includes a detailed review of sex determination in plants.
One issue is that the FDR used is unclear. It is 0.05 in page 9 but 0.01 in page 15. From Additional Table S5 it seems like the threshold used was 0.05. In addition, it would be useful to provide the RNAseq result of all genes so that interested parties can apply their own logFC and FDR thresholds when comparing studies. Finally a volcano plot that shows the effect of the threshold would also help visualising the effect of choosing different thresholds.
Page 9: "These genes" - It is unclear if this refers to 12 or 8 genes. What are the identification criteria for the 12 and 8 genes? Do the remaining 241 genes not follow these criteria?
The version of the software used is missing from all text except the Methods section. Is this acceptable?
Fig S6: Please make the new assembly to be collinear with Ha et al, which will make any comparisons easier. Ideally the raw files are as collinear as possible, but at least the figure should be so that all remaining inconsistencies are more likely to be true inversions.
Minor comments
Page 2, second last sentence "Among these DEGs" -> insert number of DEGs.
Page 5: "real-time (SMRT)" -> "PacBio", as in first paragraph of results
Page 6: "The assembly includes" -> "The assembly is comprised of", or specify what else it includes.
Page 6: delete ", in which most of the errors were from substitutions"
Page 6: The BUSCO result text is unnecessarily long. It is sufficient to say it is best that other assemblies, and use the [] format used in Fig 6 to describe the result.
Page 8, first paragraph, second last sentence: "transcriptions" -> transcription
Page 8: "((Additional Table S3)" -> "(Additional Table S3)"
Page 10: remove the non-capitalised "is a sugar transport protein that" and "is a jasmonic acid carboxyle methyltransferase that" as the information just repeats the gene name.
Page 13: "used to the train" -> "used to train"
Fig S3: "Scoffold" -> "Scaffold"

Methods

Are the methods appropriate to the aims of the study, are they well described, and are necessary controls included? Choose an item.

Conclusions

Are the conclusions adequately supported by the data shown? Choose an item.

Reporting Standards

Does the manuscript adhere to the journal's guidelines on minimum standards of reporting? Choose an item.

Choose an item.

Statistics

Are you able to assess all statistics in the manuscript, including the appropriateness of statistical tests used? Choose an item.

Quality of Written English

Please indicate the quality of language in the manuscript: Choose an item.

Declaration of Competing Interests

Please complete a declaration of competing interests, considering the following questions:

- Have you in the past five years received reimbursements, fees, funding, or salary from an organisation that may in any way gain or lose financially from the publication of this manuscript, either now or in the future?
- Do you hold any stocks or shares in an organisation that may in any way gain or lose financially from the publication of this manuscript, either now or in the future?
- Do you hold or are you currently applying for any patents relating to the content of the manuscript?
- Have you received reimbursements, fees, funding, or salary from an organization that holds or has applied for patents relating to the content of the manuscript?
- Do you have any other financial competing interests?
- Do you have any non-financial competing interests in relation to this paper?

If you can answer no to all of the above, write 'I declare that I have no competing interests' below. If your reply is yes to any, please give details below.

I declare that I have no competing interests

I agree to the open peer review policy of the journal. I understand that my name will be included on my report to the authors and, if the manuscript is accepted for publication, my named report including any attachments I upload will be posted on the website along with the authors' responses. I agree for my

report to be made available under an Open Access Creative Commons CC-BY license (http://creativecommons.org/licenses/by/4.0/). I understand that any comments which I do not wish to be included in my named report can be included as confidential comments to the editors, which will not be published.

Choose an item.

To further support our reviewers, we have joined with Publons, where you can gain additional credit to further highlight your hard work (see: https://publons.com/journal/530/gigascience). On publication of this paper, your review will be automatically added to Publons, you can then choose whether or not to claim your Publons credit. I understand this statement.

Yes Choose an item.
