# Supplementary material for: De novo genome assembly and Hi-C analysis reveal an association between chromatin architecture alterations and sex differentiation in the woody plant Jatropha curcas
Source: Gigascience. 2020 Feb 12;9(2):giaa009. doi: 10.1093/gigascience/giaa009 (PMC7014976; doi:10.1093/gigascience/giaa009)
Supplement: giaa009_Reviewer_3_Report_Original_Submission — Jian-Feng Mao, Ph.D. -- 8/11/2019 Reviewed [file giaa009_reviewer_3_report_original_submission.pdf]

Reviewer Report

Title: De novo genome assembly and Hi-C analysis reveal the association between chromatin architecture alterations and sex differentiation in the woody plant Jatropha curcas

Version: Original Submission **Date:** 8/11/2019

Reviewer name: Jian-Feng Mao, Ph.D.

Reviewer Comments to Author:

Chen et al. contribute their study on chromosome-scale genome assembly and the connection of chromatin organization and sex differentiation of Jatropha curcas, a tree with significant values. The quality of the genome assembly is distinct. And the connection of chromatin organization, gene expression and sex differentiation is a very interesting topic.

My comments

1. The current DEGs part is very general. Even though you have a previous publication on it, new DEGs result should be presented in detail other than simply some values of DEGs. In my view, the DEGs should be summarized on their functional categories (such as GO, Kegg, flower pathways, sexual determination pathways). Then, the readers could have chance to build a comprehensive picture of gene expression related to the biological process, and to the effects of chromatin organization on gene expression. If you include more gene expression (see my next comment), please provide in-depth description on gene expression and co-expression. That is not difficult.

2. Chromatin architechture alternations of sex differentiation is the core point of this article. Comparison between chromatin contact and gene expression units make the most key part of the analyses present in the study. While, there are still lacking some interesting staff, such as comparison between gene co-expression profile and chromatin organization. The published gene expression reports (including the No. 64 cited in the reference list, and some others listed in the following lines) provide enough data for a gene co-expression study. Please collect more RNA-seq data, enrich the gene expression part, construct gene co-expression profile, compare the gene co-expression and chromatin contact, make in-depth investigation and description, and raise potential hypothesis. We would like to know if such genes contacted in chromatin level go together in gene expression for example in "m-bud", "g-bud" and "m-leaf". Or which set of genes do that, which do not? This means the author make need to provide a gene co-expression study, and then for this kind of comparsion. Such kind of analyse is a rountine way to examine the connection between gene expression and chromatin organization. Please see the articles listed below.

On RNA-seq data and gene expression studies on Jatropha curcas, please collect more by googling.

(1) Global gene expression analysis of the response of physic nut (Jatropha curcas L.) to medium- and long-term nitrogen deficiency
https://journals.plos.org/plosone/article?id=10.1371/journal.pone.0182700

(2) A gene co-expression network model identifies yield-related vicinity networks in Jatropha curcas shoot system
https://www.nature.com/articles/s41598-018-27493-z

On gene co-expression and chromatin organization, also google could give more
(1) Characterization of genome-wide enhancer-promoter interactions reveals co-expression of interacting genes and modes of higher order chromatin organization
https://www.nature.com/articles/cr201215
(2) Hi-C Chromatin Interaction Networks Predict Co-expression in the Mouse Cortex
https://journals.plos.org/ploscompbiol/article?id=10.1371/journal.pcbi.1004221
(3) Human transcriptional interactome of chromatin contribute to gene co-expression
https://bmcgenomics.biomedcentral.com/articles/10.1186/1471-2164-11-704
(4) Analysis of the relationship between coexpression domains and chromatin 3D organization
https://journals.plos.org/ploscompbiol/article?id=10.1371/journal.pcbi.1005708
3. Page 5, "suggesting that chromatin organization may be different between grasses and woody plants". If you only have cases from one grass (here rice) and one woody plant (Jatropha), keep conservative and give up this tandency, as few people will agree with you.
4. I see some results not well described in the results part but in the discussion part, like all you discussed in Page 11, especially the genes IDs, I did read them in results. Please enrich the results, do not be so general. You may put them into supplementary file, if you are worrying about the space.
5. Line number is lacking. And it is very hard for reviewers to provide their comments.
6. Please replace "new Jatropha assembly" in different Figures or Additional Figures with "our assembly" or "the present assembly".
7. Additional Figures S3. I may want to see different assemblies were contrasted in one panel by overlying different items, but not in separated ones. And also transformation could applied to X or Y variables, so as to get good visulation.
8. why the assembly from Kancharla et al. 2015 was never compared in analyses presented in Figure 2, Additional Figure S3 and other Figures? but in that in Additional Table S1?
9. Additional Figures S5 is a partial one. Please provide an intact one.
10. Additional Figures S7. Please provide the contrast in one panel, not two or more.
11. Figure 5, hard to see yellow box and cyan square, only black square could I see. Please refine this figure.
12. Figure 6, hard to see red square, only dark squares I see. Please refine this figure.

Methods

Are the methods appropriate to the aims of the study, are they well described, and are necessary controls included? Choose an item.

Conclusions

Are the conclusions adequately supported by the data shown? Choose an item.

Reporting Standards

Does the manuscript adhere to the journal's guidelines on [minimum standards of reporting?]() Choose an item.

Choose an item.

Statistics

Are you able to assess all statistics in the manuscript, including the appropriateness of statistical tests used? Choose an item.

Quality of Written English

Please indicate the quality of language in the manuscript: Choose an item.

Declaration of Competing Interests

Please complete a declaration of competing interests, considering the following questions:

- Have you in the past five years received reimbursements, fees, funding, or salary from an organisation that may in any way gain or lose financially from the publication of this manuscript, either now or in the future?
- Do you hold any stocks or shares in an organisation that may in any way gain or lose financially from the publication of this manuscript, either now or in the future?
- Do you hold or are you currently applying for any patents relating to the content of the manuscript?
- Have you received reimbursements, fees, funding, or salary from an organization that holds or has applied for patents relating to the content of the manuscript?
- Do you have any other financial competing interests?
- Do you have any non-financial competing interests in relation to this paper?

If you can answer no to all of the above, write 'I declare that I have no competing interests' below. If your reply is yes to any, please give details below.

I declare that I have no competing interests

I agree to the open peer review policy of the journal. I understand that my name will be included on my report to the authors and, if the manuscript is accepted for publication, my named report including any attachments I upload will be posted on the website along with the authors' responses. I agree for my report to be made available under an Open Access Creative Commons CC-BY license (http://creativecommons.org/licenses/by/4.0/). I understand that any comments which I do not wish to be included in my named report can be included as confidential comments to the editors, which will not be published.

Choose an item.

To further support our reviewers, we have joined with Publons, where you can gain additional credit to further highlight your hard work (see: https://publons.com/journal/530/gigascience). On publication of this paper, your review will be automatically added to Publons, you can then choose whether or not to claim your Publons credit. I understand this statement.

Yes Choose an item.
